# A high frequency of detection of koala retrovirus fragments in Victorian koalas suggests historic integration of KoRV

**DOI:** 10.1099/jgv.0.002097

**Published:** 2025-04-25

**Authors:** Louize Zheng, Alistair R. Legione

**Affiliations:** 1 Asia-Pacific Centre for Animal Health, Melbourne Veterinary School, Faculty of Science, The University of Melbourne, Melbourne, Australia; 2 School of Biosciences, Faculty of Science, The University of Melbourne, Melbourne, Australia

**Keywords:** koala, koala retrovirus, recombinant koala retrovirus (recKoRV), Victoria

## Abstract

Recombinant koala retrovirus (recKoRV) is a recently discovered variant of koala retrovirus (KoRV), which likely emerged due to recombination with another retrovirus (such as Phascolarctos endogenous retrovirus). KoRV spread and endogenization in Australia were thought to be ongoing in a north to south direction given the low prevalence of the virus in southern koala populations, based on molecular detection of the *pol* gene. However, recKoRV has highlighted that fragments of KoRV with the *pol* region missing are present within southern koalas. In this study, a new 5′-region-based KoRV PCR assay was developed, capable of detecting both intact KoRV and all known variants of recKoRV. Using this assay, 319 archived DNA samples from 287 Victorian koalas were retested to investigate KoRV endogenization. We found 98.3% (282/287) of these samples were positive for the KoRV-5′ fragment, the majority of which were KoRV-*pol* negative (222/287) on prior testing. Our findings demonstrate extensive KoRV integration into the Victorian koala populations, suggestive of a historic presence of KoRV in Victorian koalas. This finding makes biological sense relative to the translocation history of Victorian koalas, compared to the prior paradigm of low virus prevalence, and provides new epidemiological and practical management implications.

## Introduction

Koala retrovirus (KoRV) is a gammaretrovirus that infects koalas (*Phascolarctos cinereus*) and replicates by integrating its proviral DNA into the host genome [1]. While most retroviruses have inserted into mammalian genomes millions of years ago and are conserved in endogenous, replication-defective forms, KoRV has remained viable in both endogenous and exogenous forms [2,3]. Similar to other gammaretroviruses that infect cats (feline leukaemia virus) [4], birds (avian leukosis virus) [5] and apes (gibbon ape leukaemia virus) [6], there is evidence suggesting the clinical correlation of KoRV with leukaemia, neoplasia and chlamydia as secondary diseases [7,8]. This makes KoRV an important area of study for the conservation of koalas as well as for understanding the endogenization process of retroviruses.

In previous studies, it was found that KoRV can be classified into different subtypes depending on the *env* sequence, which encodes for the envelope glycoprotein [9]. KoRV-A is ubiquitously found in all koala populations in Queensland and New South Wales [7,10] in the endogenous form, which is transmitted vertically [2]. In comparison, it was previously detected in only 20–60% of populations in Victoria and South Australia [11,13]. Based on the epidemiological distribution, KoRV-A is hypothesized to have originally spread from north to south, with its endogenization process currently taking place in the same direction [3,12, 14]. Moreover, as identified by other studies, there are multiple subtypes [9,15], which have likely emerged from mutations of replication competent KoRV-A within infected koalas [ 14,16 ]. These KoRV subtypes show diversity in prevalence across populations [7,11, 14]. These include a more pathogenic subtype, KoRV-B, that is found more frequently in the northern populations, similarly to KoRV-A [7,11, 17]. Hence, such geographical patterns of KoRV epidemiology can indicate the susceptibility of a chosen koala population to secondary diseases [18].

**Fig. 1.**
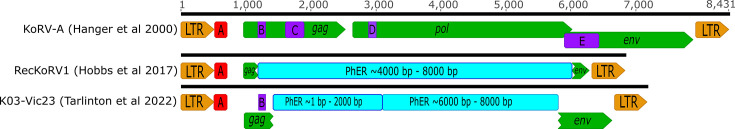
Genomic structures of KoRV [1] and two recKoRV variants (recKoRV1 [2] and southern recKoRV [20]). Red bars (**a**) represent the 5′ region amplified by the primers developed in this study. Purple bars (**B)–(**E) represent previously published target regions: (B) Gag_1 and (C) Gag 2 from [21], (D) KoRV *pol* diagnostic assay [8] and (E) KoRV *env* subtype testing [9]. PhER: Phascolarctos endogenous retroelement.

In recent studies, long-read DNA sequencing has led to the discovery of several disrupted forms of KoRV. These variants are called recombinant KoRV (recKoRV), wherein the mid-region of KoRV has recombined with the Phascolarctos endogenous retroelement (PhER) [2]. As a result, it has lost the entire *pro-pol* gene and some *gag* and some *env* regions that were critical for the virus detection in PCR-based diagnostics [8]. The initial work identified three variants of recKoRV in the koala genome (called recKoRV, recKoRV2 and recKoRV3), all with different breakpoints, and was followed by the discovery of additional variants and breakpoints in a zoological specimen (recKoRV4-18) [19] and in southern koalas (not given a numeric identifier, but referred to from here as ‘southern recKoRV’) [20]. This has raised the question of whether previously identified KoRV-negative koalas are in fact naïve to an endogenous form of this virus. The presence of a recKoRV variant in southern koalas could indicate KoRV has already entered their ancestral germlines, and the endogenization process is more advanced than previously believed. Additionally, it may suggest recKoRV is somehow providing protection against KoRV-A and KoRV-B infection in southern populations. Recently, a PCR designed to target the *gag* region of the KoRV genome found a substantial number of positive cases in previously tested KoRV*pol* negative animals in South Australia [21], however the position of those PCR primers would only allow identification of southern recKoRV and not identify recKoRV1 (Fig. 1). Therefore, to determine the prevalence of KoRV variants that lacked the *pol* target in Victorian koalas, we used 319 archived samples previously tested for the KoRV *pol* region using a newly developed PCR targeting an alternative intergenic region conserved in both intact KoRV and multiple recKoRV variants.

## Methods

### Sample collection

A total of 319 archived samples from 287 Victorian koalas were used in this research. These samples, collected between 2010 and 2015, were previously tested for the presence of KoRV provirus [11]. DNA was extracted using the Corbett Xtractor robot and Qiaxtractor VX extraction kit as per the manufacturer’s instructions and had been stored in 96-well extraction plates at −20 °C prior to this project. The sample types targeted were buffy coat and spleen samples, with incidental inclusion of whole blood, plasma and serum sample extracts if they were present in the original extraction plates. Each sample was associated with previous records of location, sex, age, *β*-actin genomic copy number per extraction and KoRV provirus copy number per extraction based on *pol*-targeted quantitative PCR (qPCR).

Additionally, initial testing utilized recently obtained diagnostic samples from koalas from Queensland and Victoria. These samples included skin, blood and spleen samples that were extracted using the Wizard^®^ Genomic DNA Purification Kit (Promega). Three samples from Victorian koalas that had previously been identified as containing recKoRV via long-read sequencing were also included in initial testing [20].

### Primer design

Two sets of primers (Table 1) were designed using primer3plus 2.3.7 [22] in Geneious Prime 2023.01 [23]. Both were developed to target a 5′ intergenic region found in all known variants of KoRV and recKoRV. For northern recKoRV variants, identified by Hobbs *et al*. [2], all KoRV LTR flanked sequences that were not complete KoRV and contained portions of PhER were extracted from the koala reference genome (GenBank assembly accession: GCF_002099425.1), including multiple recKoRV1 sequences, recKoRV2 and recKoRV3. For southern recKoRV, a reference was obtained by assembling biosample sequences of SAMN23247354, SAMN23247355, SAMN23247356 and SAMN23247357 [20] aligned against KoRV-A (NC039228) [1]. Briefly, the FASTQ reads generated in that study were assembled using Flye [24], and regions flanked by KoRV LTRs that were not complete KoRV and contained portions of PhER were extracted. All sequences were aligned using MAFFT [25] to determine conserved sites across multiple recKoRVs (FASTA sequences for all recKoRV sequences used are available in File S1, available in the online Supplementary Material). In our study, samples successfully amplified with the primers were defined to be ‘KoRV-5′ positives’.

**Table 1.** Primer designs for KoRV and KoRV-5′ detection in PCR

Primer set	Primer	Sequence	Length (bp)	G+C content (mol%)	Tm* (°C)	Hairpin Tm*/Self-dimer Tm*	Expected product size (bp)
KoRV5p_100	KoRV5p_100_F	5′-AGTAGCGGACAGACGTGT-3′	18	55.6	61.1	41.0 °C/na	131
	KoRV5p_100_R	5′-TGCAACTGTGAGATCAGAAG-3′	20	45	58.3	39.1 °C/na	
KoRV5p_200	KoRV5p_200_F	5′-CGTCCGGGATCTGAGATT-3′	18	55.6	58.9	na/7.3 °C	198
	KoRV5p_200_R	5′-ACACGTCTGTCCGCTACT-3′	18	55.6	61.1	na/na	

*Temperatures were calculated for the following conditions [30SantaLucia 1998]: 50 mM monovalent, 1.5 mM divalent, 250 nM oligo, and 0.2 mM dNTPs.

### Initial testing of primers

Conventional PCR was utilized with three diagnostic samples of unknown recKoRV status and three known recKoRV positive samples used in Tarlinton *et al*. [20] (all six were previously tested on KoRV *pol* PCR and contained three KoRV negatives and three KoRV positives). This was performed in a T100 Thermal Cycler (Bio-Rad). Each reaction mixture consisted of 5 µl template, 500 nM of each primer, 0.20 mM of each dNTP, 2.00 mM MgCl_2_, 1.5 units GoTaq Flexi DNA Polymerase (Promega) and 1×GoTaq Flexi buffer. The final volume was made up to 25 µl using nuclease-free water. The thermal profile of the PCR was as follows: initial denaturation at 95 °C (4 min), 40 cycles of denaturation at 95 °C (30 s), annealing at 55 °C (30 s) and extension at 72 °C (15 s). The final cycle was followed by a final extension at 72 °C for 5 min. Consequently, the products were visualized using a 1% w/v agarose gel electrophoresis, in conjunction with HyperLadder 100 bp (Bioline) to determine the presence of the target sequences. To confirm their specificities in target amplification, four PCR products (SAMN23247356 and SAMN23247357 amplified with each of the two primer sets) from the following section were sequenced using Sanger dideoxy sequencing at the Australian Genomic Research Facility and aligned against the original KoRV-A reference sequence (RefSeq Accession: NC039228).

### Plasmid control preparation

To create positive controls and standards for qPCR, a known recKoRV-positive sample (SAMN23247356) was amplified in the conventional PCR assay with primers KoRV5p_100 and KoRV5p_200. These PCR products were purified with the QIAquick Gel Extraction Kit (Qiagen) and then ligated into pGEM-T Easy plasmids (Promega) overnight. Ligated plasmids were transformed into NEB 5-alpha competent *E. coli* (New England BioLabs) using the manufacturer’s protocol and plated onto Luria–Bertani (LB) agar plates with ampicillin for antibiotic selection and IPTG and X-gal for blue/white selection. Selected colonies were cultured overnight at 37 °C in LB broth in the presence of ampicillin and shaking at 200 r.p.m. The plasmids were extracted from 5 ml of overnight culture using the Wizard^®^ Plus SV Miniprep DNA Purification System (Promega), and the concentration of purified plasmid was determined using a Qubit 3.0 Fluorometer (Invitrogen). Genomic copy numbers were calculated from this value, and for each plasmid, 10-fold dilutions from 10^8^ to 10^1^ copies per 5 µl were prepared, in nuclease-free water, with a QIAgility robot (Qiagen).

### PCR optimization

Positive plasmid controls (10^1^, 10^2^, 10^3^, 10^4^, 10^5^, 10^6^, 10^7^, 10^8^ copies per 5 µl) and a non-template control (nuclease-free water) were used to undertake optimization of annealing temperature (51–62 °C), primer set (KoRV5p_100, KoRV5p_200), MgCl_2_ (1.5 and 2.0 mM) and primer concentration (KoRV5p_100: 0.1, 0.2, 0.3, 0.4, 0.5 µM; KoRV5p_200: 0.25 and 0.5 µM). These were carried out with reagents in Table 2 and the basic thermal cycling conditions as described previously. Conventional PCR was used for temperature optimization and qPCR for the remaining tests. For qPCR, SYTO^™^ 9 Green Fluorescent Nucleic Acid Stain (ThermoFisher Scientific) was incorporated into the master mix at a concentration of 2.0 µM per reaction.

**Table 2.** Reagents and thermal setup in PCR optimization

	Primer		MgCl_2_ (mM)	Annealing temperature (°C)	Dilution range tested (copies/reaction)
	Set	Concn (µM)			
Annealing temperature	KoRV5p_100	0.50	2.00	53, 55, 57, 60, 62	10^1^, 10^2^, 10^3^, 10^4^
	KoRV5p_200		1.50	51, 53, 55, 58, 60	10^1^, 10^2^, 10^3^
MgCl_2_ concentration	KoRV5p_200	0.50	1.50	55	10^1^, 10^2^, 10^3^, 10^4^, 10^5^, 10^6^, 10^7^, 10^8^
			2.00		
Primer set	KoRV5p_100	0.50	1.50	55	10^1^, 10^2^, 10^3^, 10^4^, 10^5^, 10^6^, 10^7^, 10^8^
	KoRV5p_200				
Primer concentration	KoRV5p_100	0.1, 0.2, 0.3, 0.4, 0.5	2.00	55	10^1^, 10^2^, 10^3^, 10^4^, 10^5^, 10^6^, 10^7^
	KoRV5p_200	0.25, 0.50	1.50		

### Detection of KoRV-5′

The archived samples of DNA extracts were tested for endogenous KoRV-5′ using an AriaMx real-time PCR system (Agilent) with KoRV5p_200 primers under the optimized conditions (see results). A melt curve was obtained at a 0.5 °C resolution from 65 to 95 °C to confirm the target amplification. The copy numbers in the original samples were estimated against the standard curve, acquired from the positive controls of 10-fold dilutions (10^8^–10^2^ copies per reaction) in triplicate. A sample was reported as ‘KoRV-5′ positive’ if it met both of the following conditions: (1) the detected copy number was above 100 per reaction and (2) the melt curve had a dominant peak in the range of 84–86 °C. Potential positives with a melt curve peak between 83 and 87 °C, or with a copy number detected below the threshold, were assessed in gel electrophoresis to determine the KoRV-5′ positives.

### Statistical analyses

For our samples, qPCR absolute quantification was used to determine KoRV-5′ genomic target copy numbers per reaction and compared to previously obtained KoRV-*pol* and *β*-actin genomic copy numbers [11]. A crude estimate of a hypothesized recKoRV genomic copy number was obtained by subtracting the previous KoRV-*pol* copy numbers from our KoRV-5′ copy numbers. This value is reported as an estimated recKoRV copy number or ‘recKoRV’ positive. For the KoRV-5′ prevalence, a univariable regression model was used to investigate any statistical correlation of the results with the demographic (sex, age and region) and contextual (sample type and extraction plate) variables. Non-parametric statistical tests were performed with the software ggstatplot [26], as copy numbers were not normally distributed even after logarithmic conversion. *β*-actin genomic copy numbers were compared using a Kruskal–Wallis test, by sample type and by their results in *pol*- and 5′-PCR assays. Mann–Whitney U test was used to compare the KoRV-5′ and the estimated recKoRV genomic copies between KoRV-*pol* positive and negative samples. Spearman’s rank-order correlation was then performed to examine the correlation of recKoRV and KoRV-*pol* genomic copies.

## Results

### Primer specificities in the initial testing

**Fig. 2.**
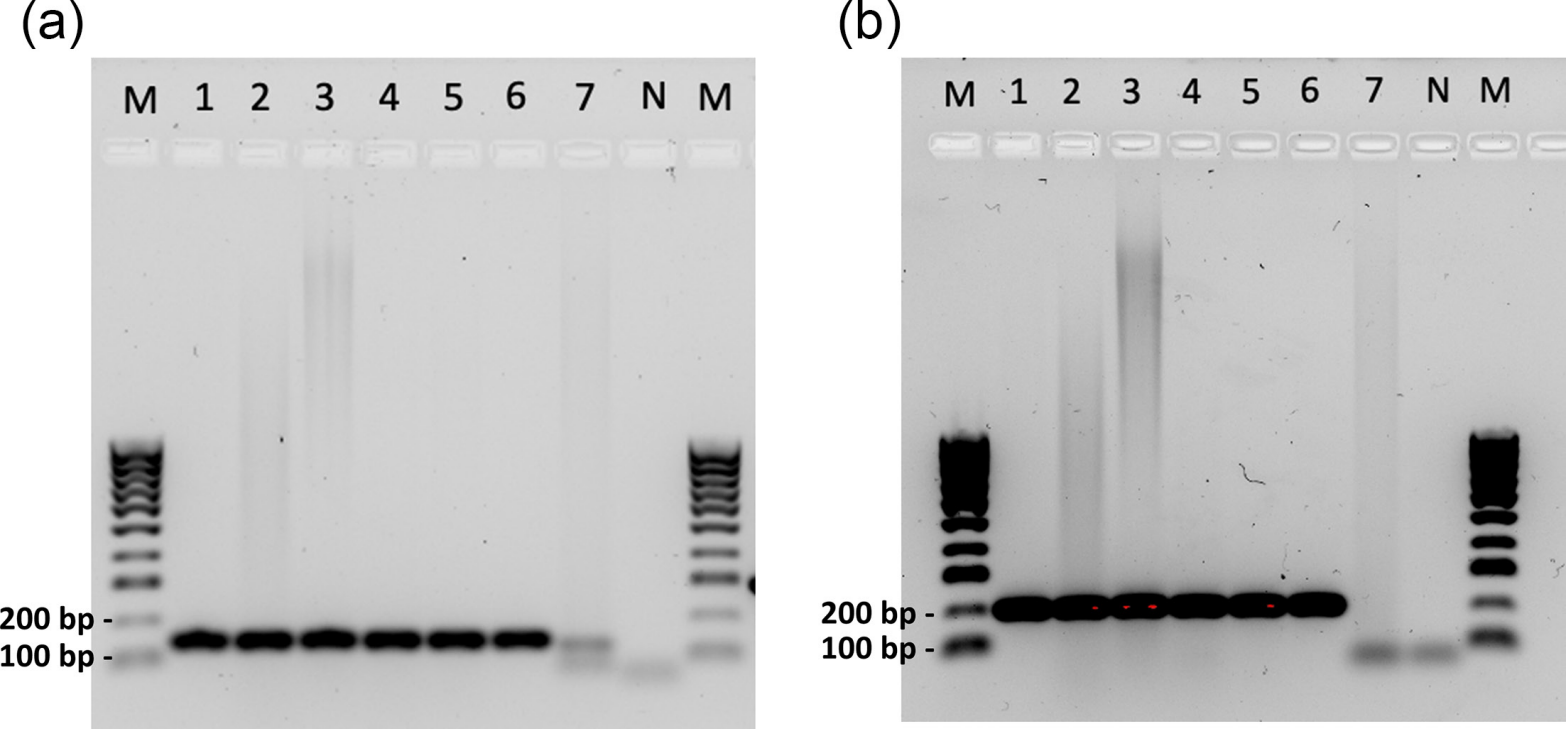
Gel images of the initial primer testing results for (**a**) KoRV5p_100 and (**b**) KoRV5p_200. M: HyperLadder 100 bp (Bioline). 1: diagnostic skin sample from Queensland (KoRV-*pol* positive). 2: diagnostic spleen sample from Victoria (KoRV-*pol* negative). 3: diagnostic blood sample from Victoria (KoRV-*pol* positive). 4: SAMN23247355 (KoRV-*pol* positive, known recKoRV positive). 5: SAMN23247356 (KoRV-*pol* negative, known recKoRV positive). 6: SAMN23247357 (KoRV-*pol* negative, known recKoRV positive). 7: non-host control. N: negative control.

All six koala samples tested positive for KoRV-5′ (Fig. 2). Two negative control samples (a non-host control using poultry cells and a nuclease-free water control) showed no target amplification. Sequenced PCR products of SAMN23247356 and SAMN23247357, for both primer sets, were both found to match our target region in recKoRV1 and all other variants available.

### Optimized PCR conditions

For the two primer sets, initial testing using 0.5 µM of primer 2 mM MgCl_2_ via qPCR provided the minimum detection limit of 10^3^ copies per reaction for KoRV5p_100 and 10^1^ copies per reaction for KoRV5p_200. Whilst KoRV5p_200 showed a single peak at target temperatures (84–86 °C) in melt curve analysis (MCA) down to dilutions of at least 10^2^ copies per reaction, KoRV5p_100 showed off-target MCA peaks in all dilutions tested. When using the primers in conventional PCR, the optimal annealing temperature was found to be 55 °C for both KoRV5p_100 and KoRV5p_200, based on the strength of the visualized bands. However, off-target products, likely dimers (<100 bp), were observed at all temperatures and for all dilutions. In qPCR, 1.50 mM MgCl_2_ showed a dominant target MCA peak for dilutions of at least 10^1^ copies per reaction, whilst at 2.00 mM MgCl_2_ a target MCA peak in the 10^1^ copies per reaction could not be detected. For KoRV5p_100, 0.2, 0.3 and 0.5 µM primer concentrations all had the minimum detection limit of 10^1^ copies per reaction in qPCR, although they created off-target MCA peaks in all dilutions. For KoRV5p_200, 0.5 µM had the same minimum detection limit, but off-target peaks were no longer present in 10^7^ and 10^8^ copy dilutions. Reducing the primer concentration to 0.25 µM resulted in KoRV5p_200 having a slightly worse minimum detection limit of 10^2^ copies per reaction (i.e. reduced sensitivity), but the detected dilutions (10^8^–10^2^ copies) did not have any major off-target peaks (increased specificity). This final condition also showed no dimers for dilutions down to 10^2^ copies per reaction when assessed on gel.

Based on the optimization results, all subsequent qPCRs were run using KoRV5p_200 primers, with 250 nM of each primer, 1.5 mM MgCl_2_, 200 nM of each dNTP, 2 µM of SYTO9, 1.5 units of GoTaq polymerase (Promega) and 1×GoTaq Flexi Buffer. All PCR reactions were made up to 20 µl with water followed by the addition of 5 µl of template. The cycling protocol was 95 °C for 4 min, followed by 40 cycles of 95 °C for 30 s, 55 °C for 30 s and 72 °C for 15 s. A final extension at 72 °C for 5 min was undertaken, followed by melt curve generation as previously described.

### KoRV-5′ prevalence

**Fig. 3.**
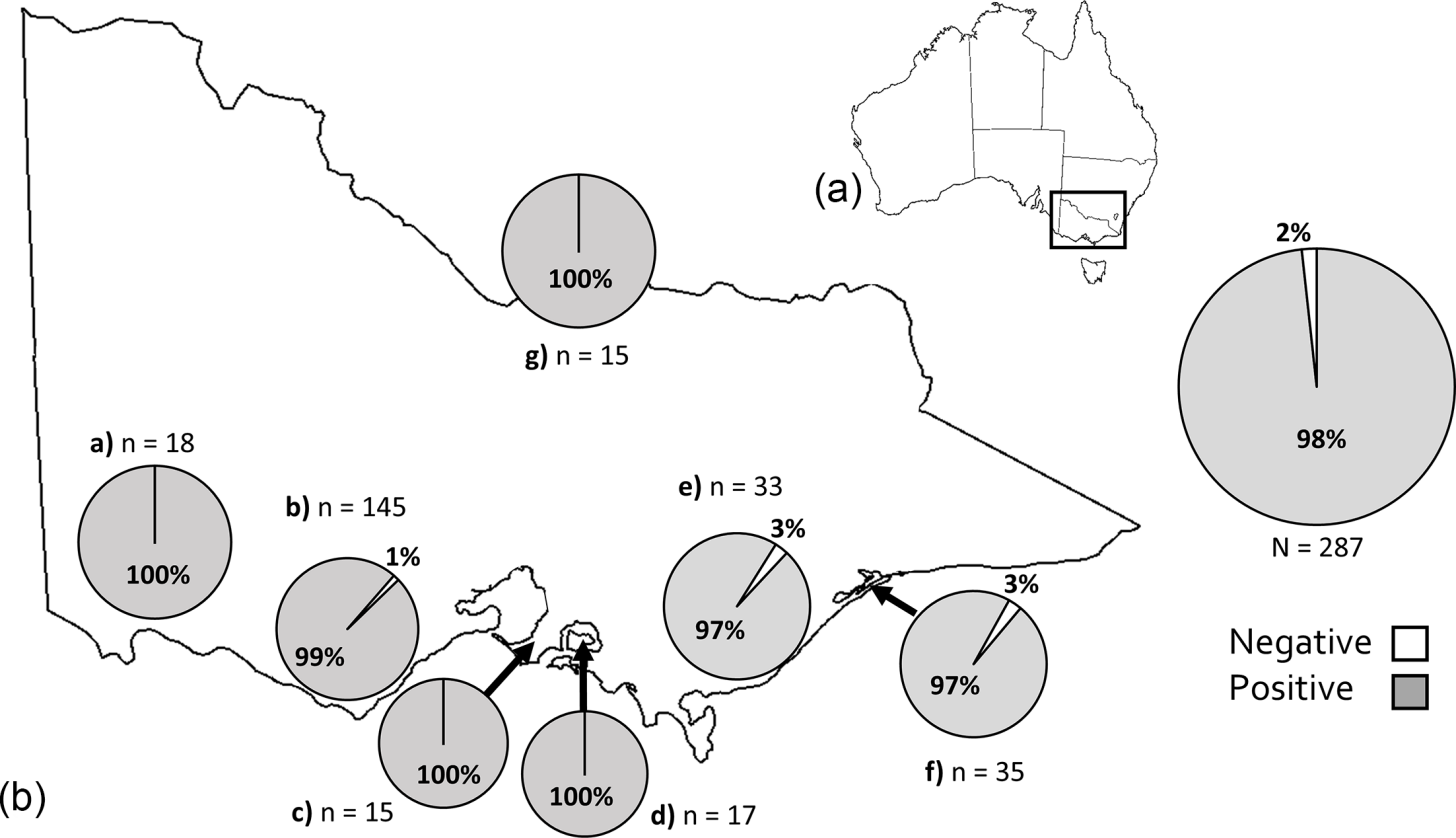
KoRV-5′ prevalence in Victorian koala populations. (**a**) Location of Victoria in Australia. (**b**) KoRV-5′ prevalence in individual koala populations which had more than ten animals tested: **a**) Far West, **b**) South Coast, **c**) Mornington Peninsula, **d**) French Island, **e**) Gippsland, **f**) Raymond Island and **g**) Far North.

The overall KoRV-5′ prevalence across Victorian koala samples was 98.3% [95% confidence interval (CI): 96–99%, 282/287], with each region (as previously described in Legione *et al*. [11]) having KoRV-5′ detection between 97 and 100% of samples tested (Fig. 3). The data are broken down by demographic and contextual variables in Table S1. No variables were significantly associated with KoRV-5′ detection.

**Fig. 4.**
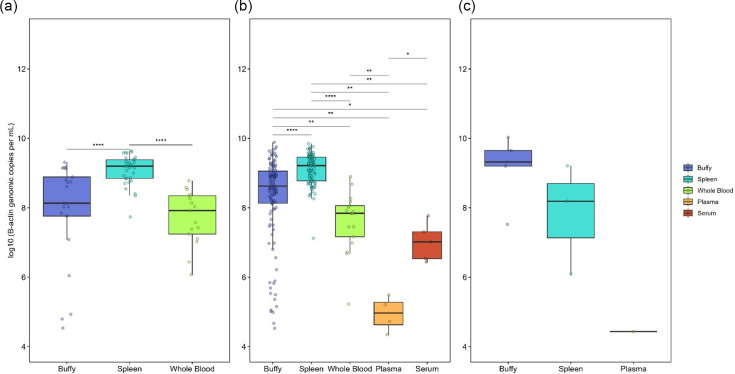
Comparison of *β*-actin genomic copy numbers in samples with different results of the KoRV-*pol* and KoRV-5′ assays. (**a**) KoRV *pol* positive, KoRV-5′ positive samples; (**b**) KoRV*-pol* negative, KoRV-5′ positive samples; (**c**) KoRV*-pol* negative, KoRV-5′ negative samples. (*: *P*≤0.05, **: *P*≤0.01, ***P≤0.001, ****P≤0.0001).

Within each sample type, the *β*-actin genomic copy numbers recorded in our prior study were found to be comparable in KoRV-5′ positive cases regardless of KoRV-*pol* status (Kruskal–Wallis test; buffy: *P*=0.148, spleen: *P*=0.76, whole blood: *P*=0.67). Between sample types, there was a significant difference between the *β*-actin genomic copy numbers (Fig. 4), and thus all further comparisons were based on a ratio of test genomic copies (KoRV-5′ or KoRV-*pol*) per *β*-actin copies detected. When normalizing the *β*-actin copies per cell (using 14 copies per cell, as per Blyton *et al*. [14]), the median copies of KoRV-5′ per cell in each population ranged from 0.355 (Gippsland) to 1.22 (Far West) (Table S2).

### Detection of two different KoRV genome fragments

Of the 287 koalas tested across Victoria, a majority were KoRV-*pol*-negative and KoRV-5′-positive (217/287, 75.6%, 95% CI: 70.2–80.5%). This was followed by animals positive for both KoRV fragments (65/287, 22.7%, 95% CI: 17.9–27.9%) and animals negative for both KoRV fragments (5/287, 1.7%, 95% CI: 0.57–4.0%). No cases were KoRV-*pol*-positive and KoRV-5′-negative.

**Fig. 5.**
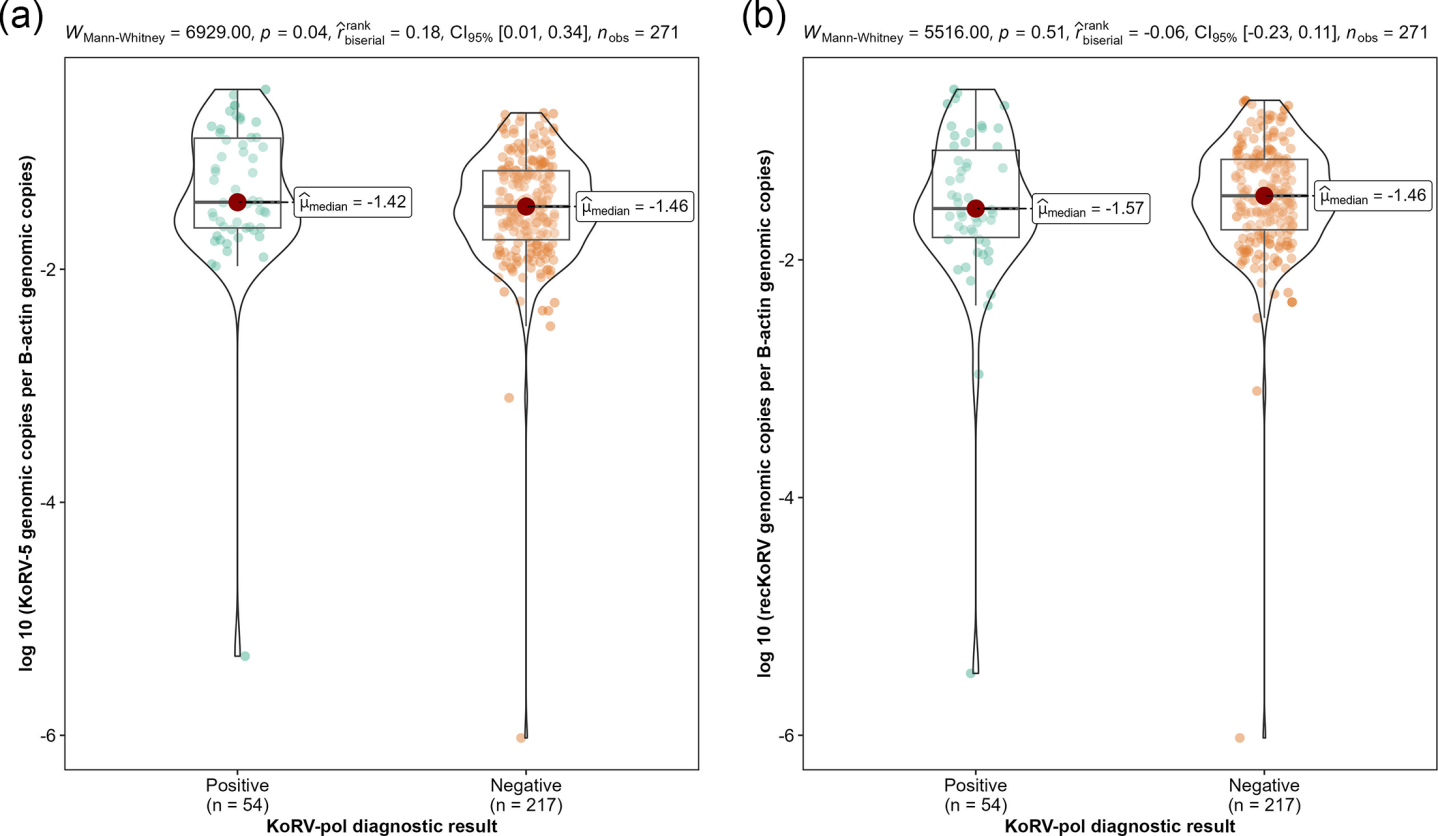
Comparison of the (**a**) KoRV-5′ and (**b**) estimated recKoRV genomic copy numbers by KoRV-*pol* status.

**Fig. 6.**
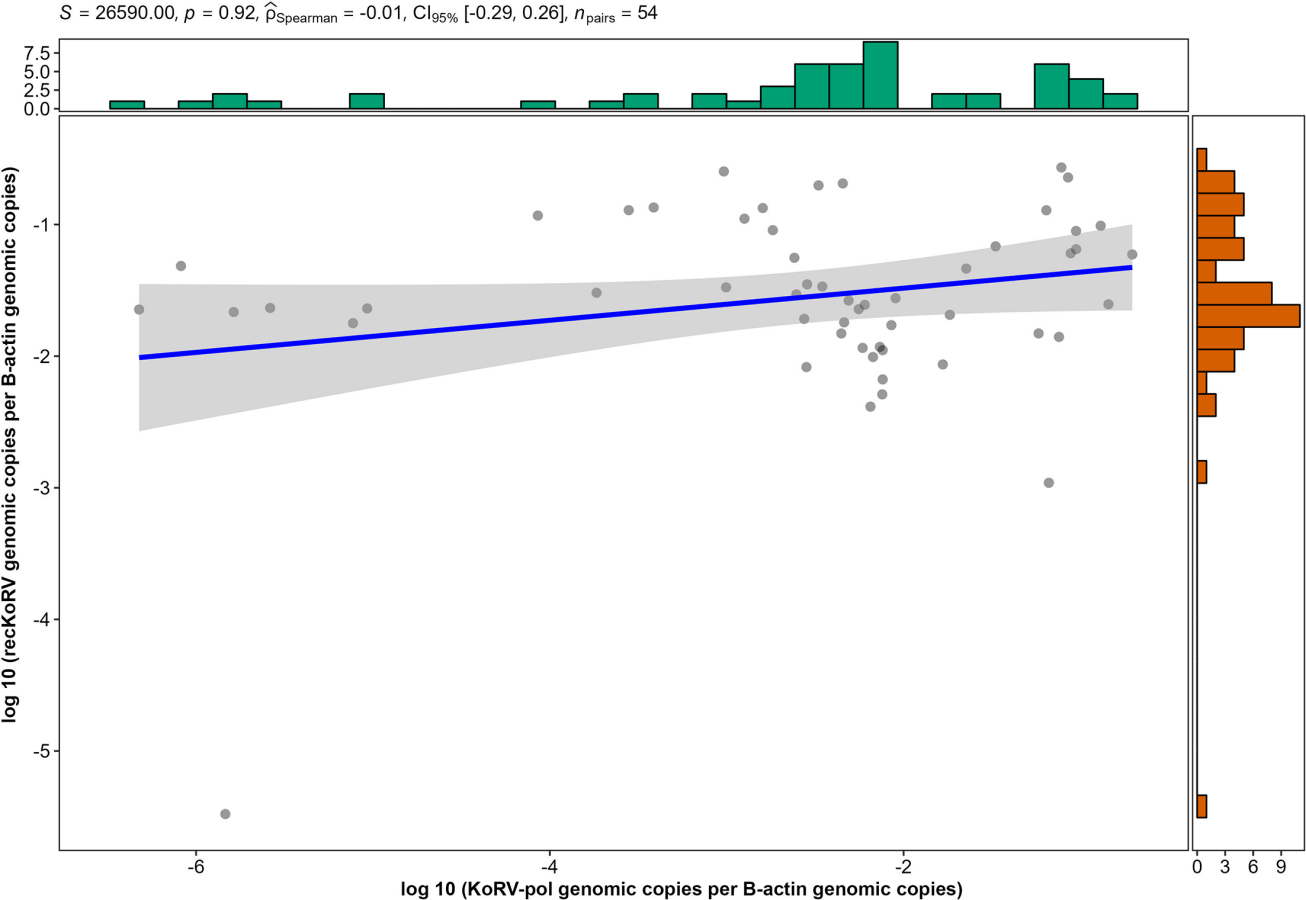
Correlation of the genomic copy numbers of estimated recKoRV and KoRV-*pol* in individual animals.

The median ratio of estimated recKoRV copies per *β*-actin, using an approximation previously outlined, was 0.034 (first quartile: 0.017, third quartile: 0.070). The median KoRV-5′ copy numbers per *β*-actin were statistically higher in KoRV positive animals compared to KoRV negative animals (Mann–Whitney U test, *P*=0.04); however, when removing KoRV-*pol* copies from the KoRV-5′ values and only looking at estimated recKoRV copy numbers, this significance was removed (Fig. 5). In KoRV-*pol* positive, suspected recKoRV positive animals, a majority (41/54, 75.9%) had more copies of suspected recKoRV than KoRV-*pol*, while the estimated recKoRV copy in each individual was at a comparable level regardless of the KoRV-*pol* abundance (Spearman=−0.01, *P*=0.92)(Fig. 6).

## Discussion

In this study, the KoRV-5′ fragment was detected in all Victorian koala populations. This is a significant finding that contrasts with the average 25% KoRV-*pol* prevalence reported previously using the same samples but an alternate target region [11]. While this assay was not able to distinguish between intact KoRV and recKoRV due to the target site used, the stark difference in the numbers provides strong support for a hypothesis that the prevalence of variants of recKoRV among Victorian koala populations is high. This was previously suspected from CRISPR-targeted sequencing of four southern koalas [20], three of which were from Victoria, as well as the detection of a *gag* region in *pol* negative South Australian koalas by Stephenson *et al*. [21]. By testing Victorian koala populations at a much larger scale, our study has provided strong supporting evidence that KoRV fragments, potentially recKoRV, in Victorian koalas are occurring at a higher frequency than previously thought. In particular, the high KoRV-5′ prevalence in French Island may explain why the KoRV prevalence had seemingly flattened at 25% in the small, closed population [11]. Given this is one of the oldest remaining populations in Victoria [27], it appears the KoRV endogenization process has begun at a much earlier time point in Victoria (before koalas were introduced to French Island in the 1890s [28]) compared to what was previously expected based on the apparent low KoRV prevalence of Victorian koalas [3,11, 12]. That KoRV-5′ was still not detected in every cell, based on comparison to *β*-actin copies, suggests that full endogenization of these KoRV fragments has not yet occurred.

Presumptively intact KoRV (determined by KoRV-5′ positive and KoRV-*pol* positive) was present in less than a quarter of the KoRV-5′ positive animals we assessed in this study. This result suggests that it is likely some variant of recKoRV is the dominant form of the virus in Victorian koala populations. A study in South Australia by Stephenson *et al*. [21] found similar results to our study, with a 99.5% detection rate of a 5′ *gag* region in koalas despite only obtaining a 41% positive result for KoRV-*pol*. That same *gag* region is present in the assembled contigs that we generated from the sequence confirmed recKoRV positive animals previously described [20]. Notably, the *gag* region targeted by the South Australian study would not have detected recKoRV1 due to the more truncated *gag* region, which is an advantage of our PCR, which targets a region present in all KoRV and recKoRV variants published to date. South Australian koalas have historical origins in Victoria, with many populations originally derived from translocations from French Island in Victoria (along with other Victorian populations) [27]. It is likely the koalas in both Victoria and South Australia have a similar endogenization pattern of southern recKoRV, but this needs to be further investigated with targeted sequencing approaches to identify both breakpoints within any recKoRV variants and their location within the koala genome.

At an individual level, where animals in Victorian populations had both the intact and broken forms of KoRV, the majority appeared to have more recKoRV than full-length KoRV genomic copies based on a comparison of qPCR results and making some assumptions that KoRV-5′ results greater than KoRV-*pol* results reflect this. If this bears out with further study, it would be in contrast to observations in northern koalas, where the recKoRV and KoRV integration sites were found in a 1:3 [19] or 1:4 [2] ratio. A higher KoRV disruption rate in Victorian populations would further emphasize the discrepancy in their true and apparent levels of KoRV endogenization. Nevertheless, the total KoRV genomic copy numbers (including intact KoRV and recKoRV) were previously found at a significantly higher level in northern koalas than southern koalas [12,14, 16]. This remains true even when the results for Victorian koalas are adjusted to include the estimated recKoRV copy numbers in our assay. It should be noted that because we utilized archived extractions, our samples may contain less DNA, due to degradation, than what the previously obtained *β*-actin numbers indicate [11]. The quantity of *β*-actin, a proxy for koala DNA abundance in extracts, differed between sample types, which was not unexpected, but highlights the need to continue considering the quantity of cellular DNA extracted when assessing retroviral provirus testing and appropriate sample selection. The few samples that tested negative for KoRV-5′ had previously been found to have similar quantities of *β*-actin to KoRV-5′ positive animals when comparing within sample types, giving some confidence that these results reflect true findings and were not negative for KoRV-5′ due to inhibited PCR or low copies of cellular DNA present. Whilst no KoRV-*pol* positive samples returned KoRV-5′-negative results, several samples had lower KoRV-5′ copies than KoRV-*pol* copies. It is most likely these samples are representative of minor degradation, as the only alternate hypothesis is that there is an undescribed integrated fragment that lacks the targeted 5′ region but contains an intact *pol* target region. Our results contrast with one study, which used PCR to detect recKoRV-1 using primers that targeted the 3′ end of the PhER region of recKoRV-1 and the 3′ end of KoRV present in recKoRV-1. In that study, recKoRV-1 was not detected in any koalas sampled from the Mornington Peninsula (*n*=5) or Gippsland (*n*=11) in Victoria [19]. The PCR primers used in that study (Ya_recKoRV1-F, Ya_recKoRV1-R, recKoRV-F1 and KoR27-R) were compared *in silico* to the assembled southern recKoRV fragments utilized in this study for primer design. We found that the southern recKoRV sequence lacked either the forward primer binding region present in recKoRV-1, which highlights the challenge of designing conventional PCR primers that span recombinant regions. These findings suggest strongly that further investigations into the full range of recKoRV variants are needed in koalas across multiple populations and that any subsequent Victorian or South Australian koalas thought to be negative for recKoRV should be further investigated using advanced methods such as CRISPR-based enrichment and long-read sequencing, which may provide the most robust evidence for the absence of integrated KoRV [20].

A recent study showed non-KoRV-A subtypes emerged only when individuals had a sufficient amount of endogenized KoRV-A (at least one copy per cell) [10,14]. If the presence of recKoRV in turn reduces the ability for KoRV to spread in a host, then this mechanism may inhibit significant KoRV-associated disease by hampering the opportunity for non-KoRV-A subtypes to emerge within the individuals. Indeed, an extremely low KoRV subtype diversity was detected previously in Victorian populations with no or few KoRV-B [10,11]. Given that KoRV-B is often more strongly associated with neoplasia [17] and chlamydia [7], it is possible that the theoretical recKoRV copy numbers in Victorian populations could reduce these risks. Nevertheless, neoplasia in koalas is most likely a result of altered host genome transcription by the effect of KoRV LTRs [2] as in similar retroviruses such as Murine leukaemia virus [29]. As recKoRV copies still possess these transcriptionally active LTRs, their presence itself might still be pathogenic. On that account, our assay, which can examine the combined amount of intact KoRV, recKoRV and other non-functional KoRV variants, may also hold significance as a diagnostic tool to indicate individual animal susceptibility to KoRV-induced neoplasia.

Our findings have significant implications for the management of koalas in Victorian populations. Current population management strategies and national risk assessments have included KoRV as an important disease, and the concept of remaining free of KoRV is considered a possibility. However, this research identifies that variants of recKoRV have a strong likelihood of being present in all koala populations, and future research should focus on the role of recKoRV variants in exogenous KoRV and disease presentation in koalas.

## Supplementary material

10.1099/jgv.0.002097 Supplementary Material 1.

## Abbreviations

CI: confidence interval
CRISPR: clustered regularly interspaced short palindromic repeats
KoRV: koala retrovirus
LB: Luria–Bertani
MCA: melt curve analysis
PhER: Phascolarctos endogenous retroelement
qPCR: quantitative PCR
recKoRV: recombinant KoRV

## References

[1] Hanger JJ, Bromham LD, McKee JJ, O’Brien TM, Robinson WF (2000). The nucleotide sequence of koala (*Phascolarctos cinereus*) retrovirus: a novel type C endogenous virus related to gibbon ape leukemia virus. J Virol.

[2] Hobbs M, King A, Salinas R, Chen Z, Tsangaras K (2017). Long-read genome sequence assembly provides insight into ongoing retroviral invasion of the koala germline. Sci Rep.

[3] Tarlinton RE, Meers J, Young PR (2006). Retroviral invasion of the koala genome. Nature.

[4] Rezanka LJ, Rojko JL, Neil JC (1992). Feline leukemia virus: pathogenesis of neoplastic disease. Cancer Invest.

[5] Neel BG, Hayward WS, Robinson HL, Fang J, Astrin SM (1981). Avian leukosis virus-induced tumors have common proviral integration sites and synthesize discrete new RNAs: oncogenesis by promoter insertion. Cell.

[6] Kawakami TG, Kollias GV, Holmberg C (1980). Oncogenicity of gibbon type-C myelogenous leukemia virus. Int J Cancer.

[7] Waugh CA, Hanger J, Loader J, King A, Hobbs M (2017). Infection with koala retrovirus subgroup B (KoRV-B), but not KoRV-A, is associated with chlamydial disease in free-ranging koalas (*Phascolarctos cinereus*). Sci Rep.

[8] Tarlinton R, Meers J, Hanger J, Young P (2005). Real-time reverse transcriptase PCR for the endogenous koala retrovirus reveals an association between plasma viral load and neoplastic disease in koalas. J Gen Virol.

[9] Chappell KJ, Brealey JC, Amarilla AA, Watterson D, Hulse L (2017). Phylogenetic diversity of Koala retrovirus within a wild Koala population. J Virol.

[10] Sarker N, Fabijan J, Seddon J, Tarlinton R, Owen H (2019). Genetic diversity of Koala retrovirus env gene subtypes: insights into northern and southern koala populations. J Gen Virol.

[11] Legione AR, Patterson JLS, Whiteley P, Firestone SM, Curnick M (2017). Koala retrovirus genotyping analyses reveal a low prevalence of KoRV-A in Victorian koalas and an association with clinical disease. J Med Microbiol.

[12] Simmons GS, Young PR, Hanger JJ, Jones K, Clarke D (2012). Prevalence of koala retrovirus in geographically diverse populations in Australia. Aust Vet J.

[13] Wedrowicz F, Saxton T, Mosse J, Wright W, Hogan FE (2016). A non-invasive tool for assessing pathogen prevalence in koala (*Phascolarctos cinereus*) populations: detection of *Chlamydia pecorum* and koala retrovirus (KoRV) DNA in genetic material sourced from scats. Conservation Genet Resour.

[14] Blyton MDJ, Young PR, Moore BD, Chappell KJ (2022). Geographic patterns of koala retrovirus genetic diversity, endogenization, and subtype distributions. Proc Natl Acad Sci USA.

[15] Shimode S, Nakagawa S, Yoshikawa R, Shojima T, Miyazawa T (2014). Heterogeneity of koala retrovirus isolates. FEBS Lett.

[16] Quigley BL, Wedrowicz F, Hogan F, Timms P (2021). Phylogenetic and geographical analysis of a retrovirus during the early stages of endogenous adaptation and exogenous spread in a new host. Mol Ecol.

[17] Quigley BL, Ong VA, Hanger J, Timms P (2018). Molecular dynamics and mode of transmission of koala retrovirus as it invades and spreads through a wild Queensland koala population. J Virol.

[18] Blyton MDJ, Pyne M, Young P, Chappell K (2022). Koala retrovirus load and non-A subtypes are associated with secondary disease among wild northern koalas. PLoS Pathog.

[19] Löber U, Hobbs M, Dayaram A, Tsangaras K, Jones K (2018). Degradation and remobilization of endogenous retroviruses by recombination during the earliest stages of a germ-line invasion. Proc Natl Acad Sci USA.

[20] Tarlinton RE, Legione AR, Sarker N, Fabijan J, Meers J (2022). Differential and defective transcription of koala retrovirus indicates the complexity of host and virus evolution. J Gen Virol.

[21] Stephenson T, Speight N, Low WY, Woolford L, Tearle R (2021). Molecular diagnosis of koala retrovirus (Korv) in south australian koalas (*Phascolarctos cinereus*). Animals.

[22] Untergasser A, Nijveen H, Rao X, Bisseling T, Geurts R (2007). Primer3Plus, an enhanced web interface to Primer3. Nucleic Acids Res.

[23] Kearse M, Moir R, Wilson A, Stones-Havas S, Cheung M (2012). Geneious basic: an integrated and extendable desktop software platform for the organization and analysis of sequence data. Bioinformatics.

[24] Kolmogorov M, Yuan J, Lin Y, Pevzner PA (2019). Assembly of long, error-prone reads using repeat graphs. Nat Biotechnol.

[25] Katoh K, Standley DM (2013). MAFFT multiple sequence alignment software version 7: improvements in performance and usability. Mol Biol Evol.

[26] Patil I (2021). Visualizations with statistical details: the “ggstatsplot” approach. J Open Source Software.

[27] Menkhorst P (2008). Royal Zoological Society of New South Wales Forum Proceedings - Too Close for Comfort.

[28] Lewis F (1934). The koala in victoria. Vic Nat.

[29] Hanecak R, Pattengale PK, Fan H (1991). Deletion of a GC-rich region flanking the enhancer element within the long terminal repeat sequences alters the disease specificity of Moloney murine leukemia virus. J Virol.

[30] SantaLucia J (1998). A unified view of polymer, dumbbell, and oligonucleotide DNA nearest-neighbor thermodynamics. Proc Natl Acad Sci USA.

