# Supplementary material for: A high frequency of detection of koala retrovirus fragments in Victorian koalas suggests historic integration of KoRV
Source: J Gen Virol. 2025 Apr 25;106(4):002097. doi: 10.1099/jgv.0.002097 (PMC12032406; doi:10.1099/jgv.0.002097)
Supplement: Supplementary Material 1. [file jgv-106-02097-s001.pdf]

Supplementary Table 1. Comparisons of KoRV-5' prevalence and associated statistical analysis results

Variable	KoRV-5' positive/n	Prevalence (%)	Odds ratio	95% CI	P value	KoRV-*pol* positive / KoRV-5' positive
Sex*						
Male	108/110	98.2	0.64	(0.08, 5.42)	0.66	24/108 (22.2%)
Female	168/170	98.8	1.00	-	-	39/168 (23.2%)
Not recorded	6/7	85.7	-	-	-	2/6 (33.3%)
Age*						
Young	54/54	100	-	-	-	15/54 (27.8%)
Mature	194/197	98.5	1.00	-	-	39/194 (20.1%)
Old	21/22	95.4	0.33	(0.04, 6.72)	0.339	6/21 (28.6%)
Not recorded	13/14	92.9	-	-	-	5/13 (38.5%)
Region * +						
Far West	18/18	100	-	-	-	6/18 (33.3%)
South Coast	143/145	98.6	1.00	-	-	23/143 (16.1%)
Far North	15/15	100	-	-	-	6/15 (40%)
Mornington Peninsula	15/15	100	-	-	-	4/15 (26.7%)
French Island	17/17	100	-	-	-	1/17 (5.9%)
Gippsland	32/33	97.0	0.45	(0.042, 9.80)	0.52	6/32 (18.8%)
Raymond Island	34/35	97.1	0.48	(0.044, 10.41)	0.55	18/34 (52.9%)
Others/ not recorded	8/9	88.9	-	-	-	1/8 (12.5%)
Extraction Plate^						
1	76/78	97.4	0.48	(0.022, 5.06)	0.55	20/76 (26.3%)
2	76/80	95.0	0.24	(0.012, 1.65)	0.20	14/76 (18.4%)
3	78/80	97.5	0.49	(0.022, 5.19)	0.56	9/78 (11.5%)
4	80/81	98.8	1.00	-	-	28/80 (35%)
Sample type^						
Buffy coat	142/147	96.6	1.00	-	-	21/142 (14.8%)
Spleen	125/128	97.7	1.47	(0.35, 7.27)	0.61	33/125 (26.4%)
Whole blood	34/34	100	-	-	-	17/34 (50%)
Plasma	4/5	80	0.14	(0.016, 3.02)	0.10	0/4 (0%)
Serum	5/5	100	-	-	-	0/5 (0%)

* Each individual animal represented once. ^ Individual animals may be represented by multiple sample types. + Regions listed from west to east geographically.

CI: confidence interval. –: not measure

Supplementary Table 2. Estimated copies of KoRV-5' per cell across different populations of Victorian koalas (based on 14 β-actin copies per cell in the diploid koala genome). Minimum values are reported to two significant digits. Total number of animals with more KoRV-5' than B-actin is provided, with percentage within each population provided.

Region	n	Median	Min	Max	Higher KoRV-5' copies (%)
South Coast	151	0.50	0.000013	5.43	35 (23%)
Far North	15	0.94	0.19	4.89	6 (40%)
Far West	28	1.22	0.0046	3.19	16 (57%)
French Island	20	0.62	0.045	4.29	9 (45%)
Gippsland	32	0.36	0.062	2.86	5 (16%)
Mornington Peninsula	17	0.32	0.13	0.68	0 (0%)
Raymond Island	39	0.89	0.000067	4.80	18 (46%)
Other/Unknown	8	0.45	0.14	1.09	2 (25%)
**Total**	310	0.52	0. 000013	5.43	91 (29%)

Supplementary data

FASTA format sequences of a subset of previously described recKoRV variants from Hobbs et al (2017) and Tarlinton et al (2022).

>RecKoRV1-06
TTGAAAGACCCCAATGTTCGGGTAGTCCTCCGACCTTGAGAAACCCTCCCAGGATCAACGC
GAACACAACTCCGGATGCAAATAGCAAGAGGTTTATTGAACTTACGAGTACTCGGGCGACT
CAGTCACCTAGAGGGACTGGCGCGCCGGGTGGATTCTTTGGTCTCATTTTATAGCAAAAAAG
CGCGGGTACAGAAGCGAGGAGCAAGATCATTGGTTAGTTTGAATAAAAACTCCGGTCACGA
GAGGGTTCTAGAAACTGCTGAGGGGCACCCTAGAAACTGTTATGAGCTCAGCTATTTCTGGG
AATTGTTTGCATAAGTCCGGGGTGGGGACTCAGGTGCATGACCACAGATATCCCGTTTGGC
ACTCCAAATCTTCCTTGTTTTTCTTGCTCTGACCTTTTACTGTGCCTGGCTGCACCTGTCCCTA
AACCTTGGGCCCTCCGGGTAGTTTCCATACTCCACGGAATGATTTCTGCCTCATGATTTCTG
CCTCCTTCATTCCCCCATTTTCTTTGAGGGTAGCTCTAATCATAGAGCAGAATTAAAGGTTATC
CTCGTTGTCTAGGGTCTGGTACTTGTGCCTGAGAACCAGAATCCTTACTGCACTAACCCTAT
CATTGATGAATTGCACTAACTTATTGATGACACAAGGCCCGAGGGTGAGTAACAGAAGGAGG
AGTAGCAGGGGACCAGCAAGGGCAGACAGTAAAGTAGTAAGCCAGGGGGAACGGTTGAA
CCATCCCTCGTACCAACTTAAATTCTTTTGGTGCTCTAACTGCCTCTTATCTAACCTTTCCTTG
AGTCTCCTGGGGCCACTCTCATCCCTATAGGCGAGTTGACGACCCAAGCTAATTTTAAATTG
TGGACTTTATTAGCTATTAGCGCGCGGCTGCCAGGGATATTCACATCCAAATCAAGCAGCC
CCCATCAGGGGTTGAGCTAGGGTTTATATAGGGAGTATACAGGACAGGGAGGGGGATGGAT
CTTTGAAGTCTGGCAAGAGGAAGAGGTTTTTTGGGGGGGGCTGGTCAAGGACACCCTTCTC
CCTCTGTGTAGATATGATAATGCTCTGCAAACTTTCTCACAATGGAGGCTTCCAGAGATATGA
TCTGAGGGCTGTGGCCTGAGCAGCCAGATAAGGGTCTTGCTAAGGAATTTGGAGTTTTCCAT
GGCCTAAAGGGTTCTACTACCCAAATGGTAAAATAATGTTCTGCATTACATTCCCCACTTCTTT
TGTGTACCACGTCAAACCCTTGACGTGGTTGGGGCTGCCTCATTATAGGGTGCAAAGAAGG
CAGACACCAGGGTCTGATAACGGGGACCTTTAACCAACAGTAACTTAACTAAATTAATACAG
GATCTAACAAAGTCAAGGAGCCGATTAACTAGACAAGGACCAAGAATTAGGGCTATAGTAAG
GAGGAGCAAAGACCCGGCTAAGGCAGAAATAAGAATGGTTAACCATGGGGAAGTCCAAAA
CAGGCTGTGGTACCAATTTTCGTTCCCTTCTCGTTCCCTCTGCCTGTCTGCAATATTTTTCCGT
GCCAGGGAAAGGCTCTCCTGAACTACACCTGAGTTATTGGCATAGAAACAGCAGGCCTCC
CCTAGGGCGGCACAAAGGCCACCTTGCCTGAGAAACAATAAGCCTAAACCCCTCTGGTTC
TGCAGAACCATCTCTGCCAAGGAGTTGACCTGTCTCTCCAGTTGGGATGTAGAGTGCTCCA
GATGACTGACTGAGGTCTGCATCGACCTGAGCACTAAGTTCCTGGTAACTGGCCTCTCCTC
GGACTAGGGCAGCGGTACCCACAGCAGTTGATCCCACTAACCCCAACCCCACTGAGGAT
AGGGATGAGGAGGGGAACGGCTCGCTTGCTTCAGTAGGACACACTGCCCTCATGACTGAA
GTACTCCCAACCCTCCAGGCCCTGAAGAAGGGAGACCCTGGGGATAATGAAGACTAATATA
CATAGAGGCTCAGATTCTTTTAGGAACACTGCAGCGGAGACACACTGTGTGATCCCATCCA
GACAGGCCCACCACGTGCCGGATGGCCCTGAATAAAAAGAGGAGGAGGATTGGGGAACA
ACCACAGTACTATTACAGACAGGAGAGTAAGGAGAAGTTTTCAGGGATGTGGTGGATGAGGT
AAGACAGGTACCTTTTCCTTGGACATCCCCCAGGGTCAACTTAGGTTGTTCCCACCGACAAT
TTTCCGAGTCTGGGCTATGGGAACCAGAACCATTAACTGCCACTCCCACATAGTATGGGGG
TTGTGCATCCATACACAGCCAGCAGCTAGGTCCTAGGTCAGGCCGGGTGGAGTTGACAAT
GCTATGGATGGCTCGGAGCAGTCTCATAATCAAGGGGTGAGGAGGATCTGCCTGAGGGGT
CCTACTAGGAGCCAGAGGAAAAGGGAAGGAGAACCAAGGAAAAGGGGGATGGGCAAGTA
GGTGGGGGCATGTGAAACAGGGAGGGGTAGTGGAGAATAGGGAAATGCAGGGAGGGTCA
AGGGAGATTGAATAGACAGTGGGAATAGATAATGGAGGGAACAGGAAGGCAGAAGTTTGAA

AGGCATCCGCACAAAATCAAGGTGGGGCTAGGACTTTCAAAAGGAGATAAAGGAAGGCCTT
TCTGGGGCCTATAGACAAAAGGGAGCCAGATCTGAATAAGATTAAAAGGTAAGGCTAAGGCT
TTCAAAGGGAGATGAGGGTCTGGGGCCTGTGAGTCAGATTCCCTTTGAGGCAAAGAGCGAA
GATCTTGGTTTGGGCCCCGCCCCCATCATCCCCCCCTCAAACAAATAGGAACCAAATTCTT
TTGGTGAAAAGGGGCGGAGGTCTCTGCTTCTGAAACTGCTTCCTGCTGGCAAGGGGCGTAG
AGCTGTCCCTGCCTCTCTGGGTCCCGTTCGAGGGGTCAAACTTCAAAACCTGGCTGATGCC
AGGTTCAGTGGACTAGAGACCTTGGGTTGGTATACAGTCATCTGGAAGTTGATGGCTCCGAC
CCTAGAAGAAAGGAATCTGGCAAGAATATTTAGTAAACATGGACCAAACAAACACAAGAGAA
GTATGATCAGTAGTGGCCTCTGTGTTTTCTTTCTGGAGTCCCTGCAAGAACTTGGTTAAGGAA
GAATTAGTGCCATCTGCCCTGAGTTCCTACTCCCCCCTTTTAGTTTTTGGCAAAGGAATCATC
CAAGGAAGAGGACTGCCGTAAGAGCCGGACCCATACTGCCAACCCCCACTAAATAGTAAA
CAAGAGCAAACAACAATATGCATACCTTACATTAATCCACAGGCCCCAATGCATACATAGAT
ATATGAAAGAATCCATTTTAACACAGTGCATATAGGATCTTAAATGTTTCAGTCATGCCATGTTT
CTCTGATGACATGGAGAAGATAAACCACAAGCCATTTCCCTTTTAACATTATTAATTGTAACAC
AACTTTAAACAAGTATGATATTACCCAAATAACAAAATAACATTCTCACAAACCCCTGACCTAA
TTGTAAAGAACTTTAACTCATATAGGAACACACACAGAACACAAGTCCCAGTTCTATAGTACC
CTAAGATGTCACATTGCCTAAGGAACATTTCCAAAATGAGCCCTAAAAAGCTTGCATGCATTT
CTACTCTTGTTCATAAAACCTCACTAAATTGGTGCAAAAGTTCTTAAATAACACAAATTCCAGA
GTAGTATTAAGGAAACTAAATTCCTAAGTATCCCAGAATCCCTCATAAGTCTTTAGTTAAAGGC
ACATAGAGATAGTTCAGTTTTCCCAGTCACACAGGACTAGGTTCAAACAAACAATACAACAAA
GTTTACTCATAATTTGCAAAATTGCATAATTACATTAAGTCAGGTACAGATTGAACCAATCAGA
GAGCTCACAATAGACTAGTTTGGAAAAGAGAGATTTACATGTAATCAGGGTAACAAAGAAAAC
CTTAGACAGGAATCATACAAATCAATACAATCAGTATTACCTATATTAACTAATATTAAGTTCCTT
GCTCATGTACAATCGGTTTAACTCATAGGCAGTTCAAAAAGGGAGCTCTGCTTCTCTGGCTTA
GTGTAAAAATCCAAAACTGTGATCTTCCTACCTAAACATGCTTAAAGCTTGGATTGCTATAATA
GATACATCTTGGAAGCCAATCATATCAGTCTAATCCTATTGCTTTTCTCAAAATTTGCTATCCTA
TTTAATTAGACATCTATCACATTACACGTTTGTCCTATTATTGTGTATAAACATTTCCTCCTTAGG
GGGATGTTATCCCTATATTATTATTATGTGGCCATGAATGTACTTGCATCCTATTACGGTAACA
CAGGGATGTTCCAGAGGGACAGGGTCATATATCATATCAGAAGGAAAGAACTTCCTTAGCTC
CATTTGATTCCAGGCATGAGTCATATCTGATAGGTAATCGCATAGTCACTGGATGTTAAAAGC
AGGGCCTGGGATTAATCAGGTGGGGATTTCCCGTGTCAGAAAGGAAATAGCACAATTGGGA
AATAGAGTCAGGAAGATCTTATCTAGGCTGTGTCCAAGGTTGTCCTCAAAAGTCTTCCCAGG
CACAGACTTCCACCTTTATCAGTTCTCAGCCTGCAGCACATGCCATTCTACTATTGGCTTTAT
GACAACTCAGACAAGTACCCTGGTAATCAAAGCTCAAAACAATAGTAAATGTCTGTCCCATA
AAATCACAAATCCTGGATAATAAGAATTTAATCTCACATTGGTAATAGTAAGAGACTGATCACA
GAAAAATACTACTGCTCTCATAATTCCTTTAAACAATGGGAACATCTTGAGGTGAGTATAATCA
ATTGAACCCCAATAAGGGCCAAAAACCTGAACTAATTCTTTTTTTTTTTTCAAGTGAACCAAAAA
CACCCCTTTCCAGGACCAGTTAGAACTGACCAACATTCTTTCTGTTTGTTATCACTGAGCCCA
AGATGTCTGGGACCAAACACTCTCCAGAACAATGGGACTTTTCTTATCTCGGTATCTTATGGA
CATAGACTATGCTATGTCATGTTAATGGTAAAAGAAACACAGATATCTTGGTCTATAATTTCAAT
GGACACTTTGACACCCTCCCGCTCTATTGAATTTACAACCCATTCAGTTAAGAAAATTTCCTTA
CCATGCCACATGTCAAACAAACATATGGAAGCTGCACTTTTTTTTTTTTTTTTTCAGGGCAGAT
GACCTTTCCTCCCCCCCATAGTTTCCAGGGATCCTGGCAAGTTTACAAAATAAGGGGAATTG
CCATGTGCCCCTCCTTTTCACACACAAGTAATCAATTAACAATCTTAGGTTTTGATATTAAAAC
AATAACCCCAAAACTTAGTTAACAATCTCACAAATTTTATAAATTATAACAAAACAATTTCAACT
GAAAATTCCCTTTCAATAGAGACAAAATTTGAAAATCTTATACTTCCTTGAATTACAATATTTAAA
AGGAAATGTTACAAAACAGGATCATATAAAATGCCCCAACAGCCAAATTGACTCCCTTTTTTTT

TTTTTGAAGACACAATTAATTTAACATTCCAGATTATAAAAGAAATTGCCCTTCCAGCAACATAA
CTAATACAATTGACCCAATAAAAAAATCTTTCTTCTTTCTAGACCCTTTCACCAGGTCCAACAG
TCTCCTGGAAGACAATCCTTTTGACTGCAGAAATAATAGAAGGATTAAAAGTCCCCTCCGGT
GGCCATCCCACTTCGAACGTGGGCCACTCGGAGGAACAGAAGGTTTGCCACTTTCCCTTTC
TTATCTCCACGGAAAGATTGTGAGCCCTTGTCTTCACGTCTTTCCAGTGATCTAGTGTAAGAG
AGAGAGGGGTCGACTCACCCTGTCCCATTCTGGGGGTCCCGAAGAGTGGAATTACGACCC
AGATAATGATTATTATTGAAATAAAAAGGAAAAAAATTATATGGCGGCGTAGACGTATATTAAAA
AGACAGGAAAAAGAGTCAGAAGGTAGGGTATGTCTCTCAAGACAGTGCAACTGTGAGATCA
GAAGAGAATTATCCCTTGCCGTGGGATCCACCAGGCGTCCCTGGTCCTCCCCTGATCCTG
GGACGTCTCCCAGGATTGCCGGGCGGTGAGCCCCCGAACACGTCTGTCCGCTACTCACG
CCTCGATCTTAGATCGATTAACCCGGAATAAACTGAAACCAAAAATGCGCGCACACCGAATA
GACAGTCAGAGTTTTCGGAGTCAGACACAGAGAGAACATAGAGCGATCGCTGGCCAGCTTA
CCTCCCGTCGGTGGTTCGGCGGTCCTTGGGTGGGAATCTCAGATCCCGGACGAGCCCCC
AAATGAAAGACCCCAATGTTCGGGTAGTCCTCCGACCTTGAGAAACCCTCCCAGGATCAAC
GCGAACACAACTCCGGATGCAAATAGCAAGAGGTTTATTGAACTTACGAGTACTCGGGCGA
CTCAGTCACCTAGAGGGACTGGCGCGCCGGGTGGATTCTTTGGTCTCATTTTATAGCAAAAA
AGCGCGGGTACAGAAGCGAGGAGCAAGATCATTGGTTAGTTTGAATAAAAACTCCGGTCAC
GAGAGGGTTCTAGAAACTGCTGAGGGGCACCCTAGAAACTGTTATGAGCTCAGCTATTTCTG
GGAATTGTTTGCATAAGTCCGGGGTGGGGACTCAGGTGCATGACCACAGATATCCCGTTTG
GCACTCCAAATCTTCCTTGTTTTTCTTGCTCTGACCTTTTACTGTGCCTGGCTGCACCTGTCC
CTAAACCTTGGGCCCTCCGGGTAGTTTCCATACTCCACGGAATGATTTCTGCCTCATGATTT
CTGCCTCCTTCAGT
>RecKoRV1-07
AATGAAGGAGGCAGAAATCATGAGGCAGAAATCATTCCGTGGAGTATGGAAACTACCCGGA
GGGCCCAAGGTTTAGGGACAGGTGCAGCCAGGCACAGTAAAAGGTCAGAGCAAGAAAAA
CAAGGAAGATTTGGAGTGCCAAACAGGATATCTGTGGTCATGCACCTGAGTCCCCACCCCG
GACTTATGCAAACAATTCCCAGAAATAGCTGAGCTCATAACAGTTTCTAGGGTGCCCCTCAG
CAGTTTCTAGAACCCTCTCGTGACCGGAGTTTTTATTCAAACTAACCAATGATCTTGCTCCTC
GCTTCTGTACCCGCGCTTTTTTGCTATAAAATGAGACCAAAGAATCCACCCGGCGCGCCAG
TCCCTCTAGGTGACTGAGTCGCCCGAGTACTCGTAAGTTCAATAAACCTCTTGCTATTTGCAT
CCGGAGTTGTGTTCGCGTTGATCCTGGGAGGGTTTCTCAAGGTCGGAGGACTACCCGAACA
TTGGGGTCTTTCATTTGGGGGCTCGTCCGGGATCTGAGATTCCCACCCAAGGACCGCCGA
ACCACCGACGGGAGGTAAGCTGGCCAGCGATCGCTCTATGTTCTCTCTGTGTCTGACTCCG
AAAACTCTGACTGTCTATTCGGTGTGCGCGCATTTTTGGTTTCAGTTTATTCCGGGTTAATCGA
TCTAAGATCGAGGCGTGAGTAGCGGACAGACGTGTTCGGGGGCTCACCGCCCGGCAATC
CTGGGAGACGTCCCAGGATCAGGGGAGGACCAGGGACGCCTGGTGGATCCCACGGCAA
GGGATAATTCTCTTCTGATCTCACAGTTGCACTGTCTTGAGAGACATACCCTACCTTCTGACT
CTTTTTCCTGTCTTTTTAATATACGTCTACGCCGCCATATAATTTTTTTCCTTTTTATTTCAATAATA
ATCATTATCTGGGTCGTAATTCCACTCTTCGGGACCCCCAGAATGGGACAGGGTGAGTCGA
CCCCTCTCTCTCTTACACTAGATCACTGGAAAGACGTGAAGACAAGGGCTCACAATCTTTCC
GTGGAGATAAGAAAGGGAAAGTGGCAAACCTTCTGTTCCTCCGAGTGGCCCACGTTCGAAG
TGGGATGGCCACCGGAGGGGACTTTTAATCCTTCTATTATTTCTGCAGTCAAAAGGATTGTCT
TCCAGGAGACTGTTGGACCTGGTGAAAGGGTCTAGAAAGAAGAAAGATTTTTTTATTGGGTCA
ATTGTATTAGTTATGTTGCTGGAAGGGCAATTTCTTTTATAATCTGGAATGTTAAATTAATTGTGT
CTTCAAAAAAAAAAAAGGGAGTCAATTTGGCTGTTGGGGCATTTTATATGATCCTGTTTTGTAA
CATTTCCTTTTAAATATTGTAATTCAAGGAAGTATAAGATTTTCAAATTTTGTCTCTATTGAAAGG
GAATTTTCAGTTGAAATTGTTTTGTTATAATTTATAAAATTTGTGAGATTGTTAACTAAGTTTTGGG

GTTATTGTTTTAATATCAAAACCTAAGATTGTTAATTGATTACTTGTGTGTGAAAAGGAGGGGCA
CATGGCAATTCCCCTTATTTTGTAAACTTGCCAGGATCCCTGGAAACTATGGGGGGGGAGGA
AAGGTCATCTGCCCTGAAAAAAAAAAAAAGTGCAGCTTCCATATGTTTGTTTGACATGTGGCAT
GGTAAGGAAATTTTCTTAACTGAATGGGTTGTAAATTCAATAGAGCGGGAGGGTGTCAAAGTG
TCCATTGAAATTATAGACCAAGATATCTGTGTTTCTTTTACCATTAACATGACATAGCATAGTCT
ATGTCCATAAGATACCGAGATAAGAAAAGTCCCATTGTTCTGGAGAGTGTTTGGTCCCAGAC
ATCTTGGGCTCAGTGATAACAAACAGAAAGAATGTTGGTCAGTTCTAACTGGTCCTGGAAAG
GGGTGTTTTTGGTTCACTTGAAAAAAAAAAAAAGAATTAGTTCAGGTTTTTGGCCCTTATTGGGGT
TCAATTGATTATACTCACCTCAAGATGTTCCCATTGTTTAAAGGAATTATGAGAGCAGTAGTATT
TTTCTGTGATCAGTCTCTTACTATTACCAATGTGAGATTAAATTCTTATTATCCAGGATTTGTGAT
TTTATGGGACAGACATTTACTATTGTTTTGAGCTTTGATTACCAGGGTACTTGTCTGAGTTGTCA
TAAAGCCAATAGTAGAATGGCATGTGCTGCAGGCTGAGAAACTGATAAAGGTGGAAGTCTGT
GCCTGGGAAGACTTTTGAGGACAACCTTGGACACAGCCTAGATAGATCTTCCTGACTCTATT
TCCCAATTGTGCTATTTCCTTTCTGACACGGGAAATCCCCACCTGATTAATCCCAGGCCCTG
CTTTTAACATCCAGTGACTATGCGATTACCTATCAGATATGACTCATGCCTGGAATCAAATGG
AGCTAAGGAAGTTCTTTCCTTCTGATATGATATATGACCCTGTCCCTCTGGAACATCCCTGTG
TTACCGTAATAGGATGCAAGTACATTCATGGCCACATAATAATAATATAGGGATAACATCCCC
CTAAGGAGGAAATGTTTAACACAATAATAGGACAAAGTGTAATGTGATAGATGTCTAATTAAAT
AGGATAGCAAATTTTGAGAAAAGCAATAGGATTAGACTGATATGATTGGCTTCCAAGATGTATC
TATCATAGCAATCCAAGCTTTAAGCATGTTTAGGTAGGAAGATCACAGTTTTGGATTTTTACACT
AAGCCAGAGAAGCAGAGCTCCCTTTTTGAACTGCCTATGAATTAAACCGATTGTACATGAGC
AAGGAACTTAATATTAGTTAATATAGGTAATACTGATTGTATTGATTTGTATGATTCCTGTCTAAG
GTTTTCTTTGTTACCCTGATTACATGTAAATCTCTCTTTTCCAAACTAGTCTATTGTGAGCTCTCT
GATTGGTTTAATCTGTACCTGACTTAATGTAATTATGCAATTTTGCAAATTATGAGTAAACTTTGT
TGTATTGTTTGTTTGAACCTAGTCCTGTGTGACTGGGAAAACTGAACTATCTCTATGTGCCTTTA
ACTAAAGACTTATGAGGGATTCTGGAATACTTAGGAATTTAGTTTCCTTAATACTACTCTGGAAT
TTGTGTTATTTAAGAACTTTTGCACCAATTTAGTGAGGTTTTATGAACAAGAGTAGAAATGCATG
CAAGCTTTTTAGGGCTCATTTTGGAAATGTTCCTTAGGCAATGTGACATCTTAGGGTACTATAG
AACTGGGACTTGTGTTCTGTGTGTGTTCCTATATGAGTTAAGTTCTTTACAATTAGGTCAGGGG
TTTGTGAGAATGTTATTTTGTTATTTGGGTAATATCATACTTGTTTAAAGTTGTGTTACAATTAATAA
TGTTAAAAGGGAAATGGCTTGTGGTTTATCTTCTCCATGTCATCAGAGAAACATGGCATGACT
GAAACATTTAAGATCCTATATGCACTGTGTTAAAATGGATTCTTTCATATATCTATGTATGCATTG
GGGCCTGTGGATTAATGTAAGGTATGCATATTGTTGTTTGCTCTTGTTTACTATTTAGTGGGGG
GTTGGCAGTATGGGTCCGGCTCTTACGGCAGTCCTCTTCCTTGGATGATTCCTTTGCCAAAA
ACTAAAAGGGGGAGTAGGAACTCAGGGCAGATGGCACTAATTCTTCCTTAACCAAGTTCTGC
AGGGACTCCAGAAAGAAAACACAGAGGCCACTACTGATCATACTTCTCTTGTGTTTGTTTGGT
CCATGTTTACTAAATATTCTTGCCAGATTCCTTTCTTCTAGGGTCGGAGCCATCAACTTCCAGA
TGACTGTATACCAACCCAAGGTCTCTAGTCCACTGAACCTGGCATCAGCCAGGTTTTGAAGT
TTGACCCCTCGAACGGGACCCAGAGAGGCAGGGACAGCTCTACGCCCCTTGCCAGCAG
GAAGCAGTTTCAGAAGCAGAGACCTCCGCCCCTTTTCACCAAAAGAATTTGGTTCCTATTTGT
TTGAGGGGGGATGATGGGGGCGGGGCCCAAACCAAGATCTTCGCTCTTTGCCTCAAAGGG
AATCTGACTCACAGGCCCCAGACCCTCATCTCCCTTTGAAAGCCTTAGCCTTACCTTTTAAT
CTTATTCAGATCTGGCTCCCTTTGTCTATAGGCCCCAGAAAGGCCTTCCTTTATCTCCTTTTGA
AAGTCCTAGCCCCACCTTGATTTTGTGCGATGCCTTTCAAACTTCTGCCTTCCTGTTCCCTCC
ATTATCTATTCCCACTGTCTATTCAATCTCCCTTGACCCCCCTGCATTTCCCTATTCTCCACTA
CCCCTCCCTGTTTCACATGCCCCCACCTACTTGCCCATCCCCCTTTTCCTTGGTTCTCCTTC
CCTTTTCCTCTGGCTCCTAGTAGGACCCCTCAGGCAGATCCTCCTCACCCCTCGATTATGA

GACTGCTCTGAGCCATCCATAGCATTGTCAACTCCACCTGGCCTGACCTAGGACCTAGCTG
CTGGCTGTGTATGGATGCACAACCCCCATACTATGTGGGAGTGGCAGTTAATGGTTCTGTTC
CCATAGCCCAGACTCGGAAAATTGTCGGTGGGAACAACCTAAGTTGACCCTGGGGGATGT
CCAAGGAAAAGGTACCTGTCTTACCTCATCCACCACATCCCTGAAAACTTCTCCTTACTCTC
CTGTCTGTAATAGTACTGTGGTTGTTCCCCAATCCTCCTCCTCTTTTTATTCAGGGCCATCCG
GCACTTGGTGGGCCTGTCTGGATGGGATCACACAGTGTGTCTCCACTGCAGTGTTCCTAAA
AGAATCTGAGCCTCTATGTATATTAGTCTCATTATCCCCAGGGTCTCCCTTCTTCAGGGCCTG
GAGGGTTGGGAGTACTTCAGTCATGAGGGCAGTGTGTCCTACTGAAGCAAGCGAGCCGTTC
CCCTCCTCATCCCTATCCTCAGTGGGGTTGGGGTTAGTGGGATCAACTGCTGTGGGTACCG
CTGCCCTAGTCCGAGGAGAGGCCAGTTACCAGGAACTTAGTGCTCAGGTCGATGCAGACC
TCAGTCAGTCATCTGGAGCACTCTACATCCCAACTGGAGAGACAGGTCAACTCCTTGGCAG
AGATGGTTCTGCAGAACCAGAGGGGTTTAGGCTTATTGTTTCTCAGGCAAGGTGGCCTTTGT
GCCGCCCTAGGGGAGGCCTGCTGTTTCTATGCCAATAACTCAGGTGTAGTTCAGGAGAGC
CTTTCCCTGGCACGGAAAAATATTGCAGACAGGCAGAGGGAACGAGAAGGGAACGAAAATT
GGTACCACAGCCTGTTTTGGACTTCCCCATGGTTAACCATTCTTATTTCTGCCTTAGCCGGGT
CTTTGCTCCTCCTTACTATAGCCCTAATTCTTGGTCCTTGTCTAGTTAATCGGCTCCTTGACTTT
GTTAGATCCTGTATTAATTTAGTTAAGTTACTGTTGGTTAAAGGTCCCCGTTATCAGACCCTGG
TGTCTGCCTTCTTTGCACCCTATAATGAGGCAGCCCCAACCACGTCAAGGGTTTGACGTGG
TACACAAAAGAAGTGGGGAATGTAATGCAGAACATTATTTTACCATTTGGGTAGTAGAACCCT
TTAGGCCATGGAAAACTCCAAATTCCTTAGCAAGACCCTTATCTGGCTGCTCAGGCCACAG
CCCTCAGATCATATCTCTGGAAGCCTCCATTGTGAGAAAGTTTGCAGAGCATTATCATATCTA
CACAGAGGGAGAAGGGTGTCCTTGACCAGCCCCCCCAAAAAAACCTCTTCCTCTTGCCA
GACTTCAAAGATCCATCCCCCTCCCTGTCCTGTATACTCCCTATATAAACCCTAGCTCAACC
CCTGATGGGGGCTGCTTGATTTGGATGTGAATATCCCTGGCAGCCGCGCGCTAATAGCTAA
TAAAGTCCACAATTTAAAATTAGCTTGGGTCGTCAACTCGCCTATAGGGATGAGAGTGGCCC
CAGGAGACTCAAGGAAAGGTTAGATAAGAGGCAGTTAGAGCACCAAAAGAATTTAAGTTGGT
ACGAGGGATGGTTCAACCGTTCCCCCTGGCTTACTACTTTACTGTCTGCCCTTGCTGGTCCC
CTGCTACTCCTCCTTCTGTTACTCACCCTCGGGCCTTGTGTCATCAATAAGTTAGTGCAATTC
ATCAATGATAGGGTTAGTGCAGTAAGGATTCTGGTTCTCAGGCACAAGTACCAGACCCTAGA
CAACGAGGATAACCTTTAATTCTGCTCTATGATTAGAGCTACCCTCAAAGAAAATGGGGGAAT
GAAGGAGGCAGAAATCATGAGGCAGAAATCATTCCGTGGAGTATGGAAACTACCCGGAGG
GCCCAAGGTTTAGGGACAGGTGCAGCCAGGCACAGTAAAAGGTCAGAGCAAGAAAAACAA
GGAAGATTTGGAGTGCCAAACAGAATATCTGTGGTCATGCACCTGAGTCCCACCCCGGACT
TATGCAAACAATTCCAGAAATAGCTGAGCTCATAACAGTTTCTAGGGTGCCCCTCAGCAGTTT
CTAGAACCCTCTCGTGACCGGAGTTTTTTTCAAACTAACCAATGATCTTGCTCCTCGCTTCTG
TACCCGCGCTTTTTTTGCTATAAAATGAGACCAAGAGAATCCACCCGGCGCGCCAGTCCCT
CTAGGTGACTGAGTCGCCCGAGTACTCGTAAGTTCAATAAACCTCTTGCTATTTGCATCCGG
AGTTGTGTTCGCGTTGATCCTGGGAGGGTTTCTCAAGGTCGGAGGACTACCCGAACATTGG
GGTCTTTCAC
>RecKoRV1-08-indels
CATGAAGGAGGCAGAAATCATGAGGCAGAAATCATTCCGTGGAGTATGGAAACTACCCGGA
GGGCCCAAGGTTTAGGGACAGGTGCAGCCAGGCACAGTAAAAGGTCAGAGCAAGAAAAA
CAAGGAAGATTTGGAGTGCCAAACGGGATATCTGTGGTCATGCACCTGAGTCCCCACCCC
GGACTTATGCAAACAATTCCCAGAAATAGCTGAGCTCATAACAGTTTCTAGGGTGCCCCTCA
GCAGTTTCTAGAACCCTCTCGTGACCGGAGTTTTTATTCAAACTAACCAATGATCTTGCTCCT
CGCTTCTGTACCCGCGCTTTTTTGCTATAAAATGAGACCAAAGAATCCACCCGGCGCGCCA
GTCCCTCTAGGTGACTGAGTCGCCCGAGTACTCGTAAGTTCAATAAACCTCTTGCTATTTGC

ATCCGGAGTTGTGTTCGCGTTGATCCTGGGAGGGTTTCTCAAGGTCGGAGGACTACCCGAA
CATTGGGGTCTTTCATTTGGGGGCTCGTCCGGGATCTGAGATTCCCACCCAAGGACCGCC
GAACCACCGACGGGAGGTAAGCTGGCCAGCGATCGCTCTATGTTCTCTCTGTGTCTGACTC
CGAAAACTCTGACTGTCTATTCGGTGTGCGCGCATTTTTGGTTTCAGTTTATTCCGGGTTAATC
GATCTAAGATCGAGGCCGTGAGTAGCGGACAGACGTGTTCGGGGGCTCACCGCCCGGCA
ATCCTGGGAGACGTCCCAGGATCAGGGGAGGACCAGGGACGCCTGGTGGATCCCACGG
CAAGGGATAATTCTCTTCTGATCTCACAGTTGCACTGTCTTGAGAGACATACCCTACCTTCTG
ACTCTTTTTCCTGTCTTTTTAATATACGTCTACGCCGCCATATAATTTTTTTCCTTTTATTTCAATA
ATAATCATTATCTGGGTCGTAATTCCACTCTTCGGGACCCCCAGAATGGGACAGGTGAGTCG
ACCCCTCTCTCTCTTACACTAGATCACTGGAAAGACGTGAAGACAAGGGCTCACAATCTTTC
CGTGGAGATAAGAAAGGGAAAGTGGCAAACCTTCTGTTCCTCCGAGTGGCCCACGTTCGAA
GTGGGATGGCCACCGGAGGGGACTTTTAATCCTTCTATTATTTCTGCAGTCAAAAGGATTGTC
TTCAGGAGACTGTTGGACCTGGTGAAAGGGTCTAGAAAGAAGAAAGATTTTTTTATTGGGTCA
ATTGTATTAGTTATGTTGCTGGAAGGGCAATTTCTTTTATAATCTAGAATGTTAAATTAATTGTGTC
TTCAAAAAAAAAAAAGGAGTCAATTTGGCTGTTGGGGCATTTTATATGATCCTGTTTTGTAACATT
TCCTTTTAAATATTGTAATTCAAGGAAGTATAATATTTTCAAATTTTGTCTCTATTGAAAGGGAATT
TTCAGTTGAAATTGTTTTGTTATAATTTATAAAATTTGTGAGATTGTTAACTAAGTTTTGGGGTTATT
GTTTTAATATCAAAACCTAAGATTGTTGATTACTTGTGTGTGAAAAGGAGGGGCACATGGCAAT
TCCCCTTATTTTGTAAACTTACCAGGATCCCTGGAAACTATGGGGGGGGGGAAAGGTCATCT
GCCCTAAAAAAAAAAAAAGTGCAGCTTCCATATGTTTGTTTGACATGTGGCATGGGAAGGGAAT
TTTCTTAATTGAATGGGTTGTAATTCAATAGAGCGGGAGGGTGTCAAAGTGTCCGTTGAAATTA
TAGACCAAGATATCTGTGTTTCTTTTACCATTAACATGACATAGCATAGTCTATGTCCATAAGAT
ACCGAGATAAGAAAAGTCCCATTGTTCTGGAGAGTGTTTGGTCCCAGACATCTTGGGCTCAG
TGATAACAAACAGAAAGAATGTTGGTCAGTTCTATCTGGTCCTGGAAAGGGGTGTTTTTGGTT
CACTTGAAAAAAAAAAAGAATTAGTTCAGGTTTTTGGCCCTTATTGGGGTTCAATTGATTATACTC
ACCTCAAGATGTTCCCATTGTTTAAAGTAATTATGAGAGCAGTAGTATTTTTCTGTGATCAGTCT
CTTACTATTACCAAGTGAGATTAAATTCTTATTATCCAGGATTTGTGATTTTATGGGACAGACATT
TACTATTGTTTTGAGCTTTGATTACCAGGGTACTTGTCTGAGTTGCCATAAAGCCAATAGTAGA
ATGGCATGTGCTGCAGGCTGATAACTGATAAAGGTGGAAGTCTGTTCCTGGGAAGACTTTTG
AGGACAACCTTGGACACAGCCTAGATAGGATCTTCTTGACTCTATTTCCCAATTGTGCTATTT
CCTTTTTGAATCAAATGGAGCTAAGGAAGTTCTTTCCTTCTGATATGATATATGACCCTGTCCC
TCTGGAACATCCCTGTGTTACCGTAATAGGATGCAAGTACATTCATGGCCACATAATAATAAT
ATAGGGATAACATCCCCCTAAGGAGGAAATGTTTACACACAATAATAGACAAAGCTGTAATGT
GATAGATGTCTAATTAAATAGGATAGCAAATTTTGAGAAAAGCAATAGGATTAGACTGATATGAT
TGGCTTCCAAGATGTATCTATCATGGCATACAAGCTTTAAGCATGTTTAGGTAGGAAGATCAC
AGTTTTGGATTTTTACACTAAGCCAGAGAAGCAGAGCTCCCTTTTTGAACTGCCTATGAATTAA
ACCGATTGTACATGAGCAAGGAACTTAATGTTAGTTAATATAGGTAATACTGATTGTATTGATTT
GTATGATTCCTGTCTAAGGTTTTCTTTGTTACCCTGATTACATGTAAATCTCTCTTTTCCAAACTA
ATCTATTGTGAGCTCTCTGATTGGTTCAATCTGTACCTGACTTAATGTAATTATGCAATTTTGCG
AATTATGAGTAAACTTTGTTGTATTGTTTGTTTGAACCTAGTCCTGTGTGACTGGGAAAACTGAA
CTATCTCTATGTGCCTTTAACTAAAGACTTATGAGGGATTCTGGAATACTTAGGAATTTAGTTTC
CTTAATACTACTCTGGAATTTGTGTTATTTAAGAACTTTTGTACCAATTTAGTGAGGTTTTATGAA
CAAGAGTAGAAATGCATGCAAGCTTTTTAGGGCTCATTTTGGAAATGTTCCTTAGGCAATGTG
ACATCTTAGGGTACTATAGAACTGGGACTTGTGTTCTGTGTGTGTTCCTATATGAGTTAAGGTT
CTTTACAATTAGGTCAGGGGTTTGTGAGAATGTTATTTTGTTATTTGGGTAATATCATACTTGTTT
AAAGTTGTGTTACAATTAATAATGTTAAAAGGGAAATGGCTTGTGGTTTATCTTCTCCATGTCAT
CAGAGAAACATGGCATGACTGAAACATTTAAGATCCTATATGCACTGTGTTAAAATGGATTCTT

TCATATATCTATGTATGTATTGGGGCCTGTGGATTAATGTAAGGTATGCATATTGTTGTTTGCTC
TTGTTTACTATTTAGTGGGGGGTTGGCAGTATGGGTCCGGCTCTTATGGCAGTCCTCTTCCTT
GGATGATTCCTTTGCCAAAAACTAAAAGGGGGGAGTAGGAACTCAGGGCAGATGGCACTAA
TTCTTCCTTAACCAAGTTCTGCAGGGACTCCAGAAAGAAAACACAGAGGCCACTACTGATCA
TACTTCTCTTGTGTTTGTTTGGTCCATGTTTACTAAATATTCTTGCCAGATTCCTTTCTTCTAGGG
TCGGAGCCATCAACTTCCAGATGACTGTATACCAACCCAAGGTCTCTAGTCCACTGAACCT
GGCATCAGCCAGGTTTTGAAGTTTGACCCCTCGAACGGGACCCAGAGAGGCAGGGACAG
CTCTACGCCCCTTGCCAGCAGGAAGCAGTTTCAGAAGCAGAGACCTCCGCCCCTTTTCAC
CAAAAGAATTTGGTTCCTATTTGTTTGAGGGGGGGATGATGGGGGCGGGGCCCAAACCAAG
ATCTTCGCTCTTTGCCTCAAAGGGAATCTGACTCACAGGCCCCAGACCCTCATCTCCCTTTG
AAAGCCTTAGCCTTACCTTTTAATCTTATTCAGATCTGGCTCCCTTTTGTCTATAGGCCCCAGA
AAGGCCTTCCTTTATCTCCTTTTGAAAGTCCTAGCCCCACCTTGATTTTGTGCAGATGCCTTTC
AAACTTCTGCCTTCCTGTTCCCTCCATTATCTATTCCCACTGTCTATTCAATCTCCCTTGACCC
CCCTGCATTTCCCTATTTTCCACTACCCCTCCCTGTTTCACATGCCCCCACCTACTTGCCCA
TCCCCCCTTTCCTTGGTTCTCCTTCCCTTTTCCTCTGGCTCCTAGTAGGACCCCTCAGGCA
GATCCTCCTCACCCCTCGATTATGAGACTGCTCCGAGCCATCCATAGCGTTGTCAACTCCA
CCCGGCCTGACCTAGGACCTAGCTGCTGGCTGTGTATGGATGCACAACCCCCACACTATG
TGGGAGTGGCAGTTAATGGTTCTGGTTCCCATAGCCCAGACTCGGAAAATTGTCGGTGGGA
ACAACCTAAGTTGACCGTGGGGGATGTCCAAGGAAAAGGTACCTGTCTTACCTCATCCACC
ACATCCCTGAAAACTTCTCCTTACTCTCCTGTCTGTAATAGTACTGTGGTTGTTCCCCAATCCT
CCTCCTCTTTTTATTCAGGGCCATCCGGCACTTGGTGGGCCTGTCTGGATGGGATCACACA
GTGTGTCTCCGCTGCAGTGTTCCTAAAAGAATCTGAGCCTCTATGTATATTAGTCTTCATTATC
CCCAGGGTCTCCCTTCTTCAGGGCCTGGAGGGTTGGGAGTACTTCAGTCATGAGGGCAGT
GTGTCCTACTGAAGCAAGCGAGCCGTTCCCCTCCTCATCCCTATCCTCAGTGGGGTTGGG
GTTAGTGGGATCAACTGCTGTGGGTACCGCTGCCCTAGTCCGAGGAGAGGCCAGTTACCA
GGAACTTAGTGCTCAGGTCGATGCAGACCTCAGTCAGTCATCTGGAGCACTCTATATCCCA
ACTGGAGAGACAGGTCAACTCCTTGGCAGAGATGGTTCTGCAGAACCAGAGGGGTTTAGG
CTTATTGTTTCTCAGGCAAGGTGGCCTTTGTGCCGCCCTAGGGGAGGCCTGCTGTTTCTATG
CCAATAACTCAGGTGTAGTTCAGGAGAGCCTTTCCCTGGCACGGAAAAATATTGCAGACAG
GCAGAGGGAACGAGAAGGGAACGAAAATTGGTACCACAGCCTGTTTGGACTTCCCCATGG
TTAACCATTCTTATTTCTGCCTTAGCCGGGTCTTTGCTCCTCCTTACTATAGCCCTAATTCTTG
GTCCTTGTCTAGTTAATCGGCTCCTTGACTTTGTTAGATCCTGTATTAATTTAGTTAAGTTACTGT
TGGTTAAAGGTCCCCGTTATCAGACCCTGGTGTCTGCCTTCTTTGCACCCTATAATGAGGCA
GCCCCAACCACGTCAAGGGTTTGACGTGGTACACAAAAGAAGTGGGGAATGTAATGCAGAA
CATTATTTTACCATTTGGGTAGTAGAACCCTTTAGGCCATGGAAAACTCCAAATTCCTTAGCAA
GACCCTTATCTGGCTGCTCAGGCCACAGCCCTCAGATCATATCTCTGGAAGCCTCCATTGT
GAGAAAGTTTGCAGAGCATTATCATATCTACACAGAGGGAGAAGGGTGTCCTTGACCAGCC
CCCCCCAAAAAAACCTCTTCCTCTTGCCAGACTTCAAAGATCCATCCCCCTCCCTGTCCTG
TATACTCCCTATATAAACCCTAGCTCAACCCCTGATGGGGGCTGCTTGATTTGGATGTGAATA
TCCCTGGCAGCCGCGCGCTAATAGCTAATAAAGTCCACAATTTAAAATTAGCTTGGGTCGTC
AACTCGCCTATAGGGATGAGAGTGGCCCCAGGAGACTCAAGGAAAGGTTAGATAAGAGGC
AGTTAGAGCACCAAAAGAATTTAAGTTGGTACGAGGGATGGTTCAACCGTTCCCCCTGGCTT
ACTACTTTACTGTCTGCCCTTGCTGGTCCCCTGCTACTCCTCCTTCTGTTACTCACCCTCGG
GCCTTGTGTCATCAATAAGTTAGTGCAATTCATCAATGATAGGGTTAGTGCAGTAAGGATTCTG
GTTCTCAGGCACAAGTACCAGACCCTAGACAACGAGGATAACCTTTAATTCTGCTCTATGATT
AGAGCTACCCTCAAAGAAAATGGGGGAATGAAGGAGGCAGAAATCATGAGGCAGAAATCAT
TCCGTGGAGTATGGAAACTACCCGGAGGGCCCAAGGTTTAGGGACAGGTGCAGCCAGGC

ACAGTAAAAGGTCAGAGCAAGAAAAACAAGGAAGATTTGGAGTGCCAAACGGGATATCTGT
GGTCATGCACCTGAGTCCCCACCCCGGACTTATGCAAACAATTCCCAGAAATAGCTGAGCT
CATAACAGTTTCTAGGGTGCCCCTCAGCAGTTTCTAGAACCCTCTCGTGACCGGAGTTTTTAT
TCAAACTAACCAATGATCTTGCTCCTCGCTTCTGTACCCGCGCTTTTTTGCTATAAAATGAGA
CCAAAGAATCCACCCGGCGCGCCAGTCCCTCTAGGTGACTGAGTCGCCCGAGTACTCGT
AAGTTCAATAAACCTCTTGCTATTTGCATCCGGAGTTGTGTTCGCGTTGATCCTGGGAGGGTT
TCTCAAGGTCGGAGGACTACCCGAACATTGGGGTCTTTCAG
>RecKoRV1-09
AGTGAAGGAGGCAGAAATCATGAGGCAGAAATCATTCCGTGGAAACTACCCGGAGGGCCC
AAGGTTTAGGGACAGGTGCAGCCAGGCACAGTAAAAGGTCAGAGCAAGAAAAACAAGGAA
GATTTGGAGTGCCAAACGGGATATCTGTGGTCATGCACCTGAGTCCCCACCCCGGACTTAT
GCAAACAATTCCCAGAAATAGCTGAGCTCATAACAGTTTCTAGGGTGCCCCTCAGCAGTTTC
TAGAACCCTCTCGTGACCGGAGTTTTTATTCAAACTAACCAATGATCTTGCTCCTCGCTTCTG
TACCCGCGCTTTTTTGCTATAAAATGAGACCAAAGAATCCACCCGGCGCGCCAGTCCCTCT
AGGTGACTGAGTCGCCCGAGTACTCGTAAGTTCAATAAACCTCTTGCTATTTGCATCCGGAG
TTGTGTTCGCGTTGATCCTGGGAGGGTTTCTCAAGGTCGGAGGACTACCCGAACATTGGGG
TCTTTCATTTGGGGGCTCGTCCGGGATCTGAGATTCCCACCCAAGGACCGCCGAACCACC
GACGGGAGGTAAGCTGGCCAGCGATCGCTCTATGTTCTCTCTGTGTCTGACTCCGAAAACT
CTGACTGTCTATTCGGTGTGCGCGCATTTTTGGTTTCAGTTTATTCCGGGTTAATCGATCTAAG
ATCGAGGCGTGAGTAGCGGACAGACGTGTTCGGGGGCTCACCGCCCGGCAATCCTGGGA
GACGTCCCAGGATCAGGGGAGGACCAGGGACGCCTGGTGGATCCCACGGCAAGGGATA
ATTCTCTTCTGATCTCACAGTTGCACTGTCTTGAGAGACATACCCTACCTTCTGACTCTTTTTC
CTGTCTTTTTAATATACGTCTACGCCGCCATATAATTTTTTTCCTTTTTATTTCAATAATAATCATTA
TCTGGGTCGTAATTCCACTCTTCGGGACCCCCAGAATGGGACAGGGTGAGTCGACCCCTC
TCTCTCTTACACTAGATCACTGGAAAGACGTGAAGACAAGGGCTCACAATCTTTCCGTGGAG
ATAAGAAAGGGAAAGTGGCAAACCTTCTGTTCCTCCGAGTGGCCCACGTTCGAAGTGGGAT
GGCCACCGGAGGGGACTTTTAATCCTTCTATTATTTCTGCAGTCAAAAGGATTGTCTTCCAGG
AGACTGTTGGACCTGGTGAAAGGGTCTAGAAAGAAGAAAGATTTTTTTATTGGGTCAATTGTAT
TAGTTATGTTGCTGGAAGGGCAATTTCTTTTATAATCTGGAATGTTAAATTAATTGTGTCTTCAAA
AAAAAAAAAGGGAGTCAATTTGGCTGTTGGGGCATTTTATATGATCCTGTTTTGTAACATTTCCT
TTTAAATATTGTAATTCAAGGAAGTATAAGATTTTCAAATTTTGTCTCTATTGAAAGGGAATTTTCA
GTTGAAATTGTTTTGTTATAATTTATAAAATTTGTGAGATTGTTAACTAAGTTTTGGGGTTATTGTTT
TAATATCAAAACCTAAGATTGTTAATTGATTACTTGTGTGTGAAAAGGAGGGGCACATGGCAAT
TCCCCTTATTTTGTAAACTTGCCAGGATCCCTGGAAACTATGGGGGGGGAGGAAAGGTCATC
TGCCCTGAAAAAAAAAAAAAAGTGCAGCTTCCATATGTTTGTTTGACATGTGGCATGGTAAGG
AAATTTTCTTAACTGAATGGGTTGTAAATTCAATAGAGCGGGAGGGTGTCAAAGTGTCCATTGA
AATTATAGACCAAGATATCTGTGTTTCTTTTACCATTAACATGACATAGCATAGTCTATGTCCAT
AAGATACCGAGATAAGAAAAGTCCCATTGTTCTGGAGAGTGTTTGGTCCCAGACATCTTGGG
CTCAGTGATAACAAACAGAAAGAATGTTGGTCAGTTCTAACTGGTCCTGGAAAGGGGTGTTTT
TGGTTCACTTGAAAAAAAAAAAAAGAATTAGTTCAGGTTTTTGGCCCTTATTGGGGTTCAATTGAT
TATACTCACCTCAAGATGTTCCCATTGTTTAAAGGAATTATGAGAGCAGTAGTATTTTTCTGTGA
TCAGTCTCTTACTATTACCAATGTGAGATTAAATTCTTATTATCCAGGATTTGTGATTTTATGGGA
CAGACATTTACTATTGTTTTGAGCTTTGATTACCAGGGTACTTGTCTGAGTTGTCATAAAGCCA
ATAGTAGAATGGCATGTGCTGCAGGCTGAGAACTGATAAAGGTGGAAGTCTGTGCCTGGGA
AGACTTTTGAGGACAACCTTGGACACAGCCTAGATAAGATCTTCCTGACTCTATTTCCCAATT
GTGCTATTTCCTTTCTGACACGGGAAATCCCCACCTGATTAATCCCAGGCCCTGCTTTTAACA
TCCAGTGACTATGCGATTACCTATCAGATATGACTCATGCCTGGAATCAAATGGAGCTAAGG

AAGTTCTTTCCTTCTGATATGATATATGACCCTGTCCCTCTGGAACATCCCTGTGTTACCGTAA
TAGGATGCAAGTACATTCATGGCCACATAATAATAATATAGGGATAACATCCCCCTAAGGAG
GAAATGTTTATACACAATAATAGGACAAACGTGTAATGTGATAGATGTCTAATTAAATAGGATAG
CAAATTTTGAGAAAAGCAATAGGATTAGACTGATATGATTGGCTTCCAAGATGTATCTATTATAG
CAATCCAAGCTTTAAGCATGTTTAGGTAGGAAGATCACAGTTTTGGATTTTTACACTAAGCCAG
AGAAGCAGAGCTCCCTTTTTGAACTGCCTATGAGTTAAACCGATTGTACATGAGCAAGGAAC
TTAATATTAGTTAATATAGGTAATACTGATTGTATTGATTTGTATGATTCCTGTCTAAGGTTTTCTTT
GTTACCCTGATTACATGTAAATCTCTCTTTTCCAAACTAGTCTATTGTGAGCTCTCTGATTGGTT
CAATCTGTACCTGACTTAATGTAATTATGCAATTTTGCAAATTATGAGTAAACTTTGTTGTATTGTT
TGTTTGAACCTAGTCCTGTGTGACTGGGAAAACTGAACTATCTCTATGTGCCTTTAACTAAAGA
CTTATGAGGGATTCTGGGATACTTAGGAATTTAGTTTCCTTAATACTACTCTGGAATTTGTGTTAT
TTAAGAACTTTTGCACCAATTTAGTGAGGTTTTATGAACAAGAGTAGAAATGCATGCAAGCTTTT
TAGGGCTCATTTTGGAAATGTTCCTTAGGCAATGTGACATCTTAGGGTACTATAGAACTGGGA
CTTGTGTTCTGTGTGTGTTCCTATATGAGTTAAAGTTCTTTACAATTAGGTCAGGGGTTTGTGAG
AATGTTATTTTGTTATTTGGGTAATATCATACTTGTTTAAAGTTGTGTTACAATTAATAATGTTAAAA
GGGAAATGGCTTGTGGTTTATCTTCTCCATGTCATCAGAGAAACATGGCATGACTGAAACATT
TAAGATCCTATATGCACTGTGTTAAAATGGATTCTTTCATATATCTATGTATGCATTGGGGCCTG
TGGATTAATGTAAGGTATGCATATTGTTGTTTGCTCTTGTTTACTATTTAGTGGGGGGTTGGCAG
TATGGGTCCGGCTCTTACGGCAGTCCTCTTCCTTGGATGATTCCTTTGCCAAAAACTAAAAG
GGGGGAGTAGGAACTCAGGGCAGATGGCACTAATTCTTCCTTAACCAAGTTCTTGCAGGGA
CTCCAGAAAGAAAACACAGAGGCCACTACTGATCATACTTCTCTTGTGTTTGTTTGGTCCATG
TTTACTAAATATTCTTGCCAGATTCCTTTCTTCTAGGGTCGGAGCCATCAACTTCCAGATGACT
GTATACCAACCCAAGGTCTCTAGTCCACTGAACCTGGCATCAGCCAGGTTTTGAAGTTTGAC
CCCTCGAACGGGACCCAGAGAGGCAGGGACAGCTCTACGCCCCTTGCCAGCAGGAAGC
AGTTTCAGAAGCAGAGACCTCCGCCCCTTTTCACCAAAAGAATTTGGTTCCTATTTGTTTGAG
GGGGGGATGATGGGGGCGGGGCCCAAACCAAGATCTTCGCTCTTTGCCTCAAAGGGAATC
TGACTCACAGGCCCCAGACCCTCATCTCCCTTTGAAAGCCTTAGCCTTACCTTTTAATCTTAT
TCAGATCTGGCTCCCTTTTGTCTATAGGCCCCAGAAAGGCCTTCCTTTATCTCCTTTTGAAAG
TCCTAGCCCCACCTTGATTTTGTGCGGATGCCTTTCAAACTTCTGCCTTCCTGTTCCCTCCAT
TATCTATTCCCACTGTCTATTCAATCTCCCTTGACCCTCCCTGCATTTCCCTATTCTCCACTAC
CCCTCCCTGTTTCACATGCCCCCACCTACTTGCCCATCCCCCCTTTTCCTTGGTTCTCCTTC
CCTTTTCCTCTGGCTCCTAGTAGGACCCCTCAGGCAGATCCTCCTCACCCCTTGATTATGAG
ACTGCTCCGAGCCATCCATAGCATTGTCAACTCCACCCGGCCTGACCTAGGACCTAGCTG
CTGGCTGTGTATGGATGCACAACCCCCATACTATGTGGGAGTGGCAGTTAATGGTTCTGGTT
CCCATAGCCCAGACTCGGAAAATTGTCGGTGGGAACAACCTAAGTTGACCCTGGGGGATG
TCCAAGGAAAAGGTACCTGTCTTACCTCATCCACCACATCCCTGAAAACTTCTCCTTACTCT
CCTGTCTGTAATAGTACTGTGGTTGTTCCCCAATCCTCCTCCTCTTTTTATTCAGGGCCATCC
GGCACGTGGTGGGCCTGTCTGGATGGGATCACACAGTGTGTCTCCGCTGCAGTGTTCCTAA
AAGAATCTGAGCCTCTATGTATATTAGTCTTCATTATCCCCAGGGTCTCCCTTCTTCAGGGCC
TGGAGGGTTGGGAGTACTTCAGTCATGAGGGCAGTGTGTCCTACTGAAGCAAGCGAGCCGT
TCCCCTCCTCATCCCTATCCTCAGTGGGGTTGGGGTTAGTGGGATCAACTGCTGTGGGTAC
CGCTGCCCTAGTCCGAGGAGAGGCCAGTTACCAGGAACTTAGTGCTCAGGTCGATGCAGA
CCTCAGTCAGTCATCTGGAGCACTCTACATCCCAACTGGAGAGACAGGTCAACTCCTTGGC
AGAGATGGTTCTGCAGAACCAGAGGGGTTTAGGCTTATTGTTTCTCAGGCAAGGTGGCCTTT
GTGCCGCCCTAGGGGAGGCCTGCTGTTTCTATGCCAATAACTCAGGTGTAGTTCAGGAGAG
CCTTTCCCTGGCACGGAAAAATATTGCAGACAGGCAGAGGGAACGAGAAGGGAACGAAAA
TTGGTACCACAGCCTGTTTTGGACTTCCCCATGGTTAACCATTCTTATTTCTGCCTTAGCCGG

GTCTTTGCTCCTCCTTACTATAGCCCTAATTCTTGGTCCTTGTCTAGTTAATCGGCTCCTTGAC
TTTGTTAGATCCTGTATTAATTTAGTTAAGTTACTGTTGGTTAAAGGTCCCCGTTATCAGACCCT
GGTGTCTGCCTTCTTTGCACCCTATAATGAGGCAGCCCCAACCACGTCAAGGGTTTGACGT
GGTACACAAAAGAAGTGGGGAATGTAATGCAGAACATTATTTTACCATTTGGGTAGTAGAACC
CTTTAGGCCATGGAAAACTCCAAATTCCTTAGCAAGACCCTTATCTGGCTGCTCAGGCCACA
GCCCTCAGATCATATCTCTGGAAGCCTCCATTGTGAGAAAGTTTGCAGAGCATTATCATATCT
ACACAGAGGGAGAAGGGTGTCCTTGACCAGCCCCCCCAAAAAACCTCTTCCTCTTGCCAG
ACTTCAAAGATCCATCCCCCTCCCTGTCCTGTATACTCCCTATATAAACCCTAGCTCAACCC
CTGATGGGGGCTGCTTGATTTGGATGTGAATATCCCTGGCAGCCGCGCGCTAATAGCTAATA
AAGTCCACAATTTAAAATTAGCTTGGGTCGTCAACTCGCCTATAGGGATGAGAGTGGCCCCA
GGAGACTCAAGGAAAGGTTAGATAAGAGGCAGTTAGAGCACCAAAAGAATTTAAGTTGGTAC
GAGGGATGGTTCAACCGTTCCCCCTGGCTTACTACTTTACTGTCTGCCCTTGCTGGTCCCCT
GCTACTCCTCCTTCTGTTACTCACCCTCGGGCCTTGTGTCATCAATAAGTTAGTGCAATTCAT
CAATGATAGGGTTAGTGCAGTAAGGATTCTGGTTCTCAGGCACAAGTACCAGACCCTAGACA
ACGAGGATAACCTTTAATTCTGCTCTATGATTAGAGCTACCCTCAAAGAAAATGGGGGAATGA
AGGAGGCAGAAATCATGAGGCAGAAATCATTCCGTGGAAACTACCCGGAGGGCCCAAGGT
TTAGGGACAGGTGCAGCCAGGCACAGTAAAGGTCAGAGCAAGAAAAACAAGGAAGATTTG
GAGTGCCAAACGGGATATCTGTGGTCATGCACCTGAGTCCCCACCCCGGACTTATGCAAA
CAATTCCCAGAAATAGCTGAGCTCATAACAGTTTCTAGGGTGCCCCTCAGCAGTTTCTAGAA
CCCTCTCGTGACCGGAGTTTTTATTCAAACTAACCAATGATCTTGCTCCTCGCTTCTGTACCC
GCGCTTTTTTGCTATAAAATGAGACCAAAGAATCCACCCGGCGCGCCAGTCCCTCTAGGTG
ACTGAGTCGCCCGAGTACTCGTAAGTTCAATAAACCTCTTGCTATTTGCATCCGGAGTTGTGT
TCGCGTTGATCCTGGGAGGGTTTCTCAAGGTCGGAGGACTACCCGAACATTGGGGTCTTTC
AG
>RecKoRV1-10
ATTGAAGGAGGCAGAAATCATGAGGCAGAAATCATTCCGTGGAGTATGGAAACTACCCGGA
GGGCCCAAGGTTTAGGGACAGGTGCAGCCAGGCACAGTAAAGGTCAGAGCAAGAAAAA
CAAGGAAGATTTGGAGTGCCAAACGGGATATCTGTGGTCATGCACCTGAGTCCCCACCCC
GGACTTATGCAAACAATTCCCAGAAATAGCTGAGCTCATAACAGTTTCTAGGGTGCCCCTCA
GCAGTTTCTAGAACCCTCTCGTGACCGGAGTTTTTATTCAAACTAACCAATGATCTTGCTCCT
CGCTTCTGTACCCGCGCTTTTTTGCTATAAAATGAGACCAAAGAATCCACCCGGCGCGCCA
GTCCCTCTAGGTGACTGAGTCGCCCGAGTACTCGTAAGTTCAATAAACCTCTTGCTATTTGC
ATCCGGAGTTGTGTTCGCGTTGATCCTGGGAGGGTTTCTCAAGGTCGGAGGACTACCCGAA
CATTGGGGTCTTTCATTTGGGGGCTCGTCCGGGATCTGAGATTCCCACCCAAGGACCGCC
GAACCACCGACGGGAGGTAAGCTGGCCAGCGATCGCTCTATGTTCTCTCTGTGTCTGACTC
CGAAAACTCTGACTGTCTATTCGGTGTGCGCGCATTTTTGGTTTCAGTTTATTCCGGGTTAATC
GATCTAAGATCGAGGCGTGAGTAGCGGACAGACGTGTTCGGGGGCTCACCGCCCGGCAA
TCCTGGGAGACGTCCCAGGATCAGGGGAGGACCAGGGACGCCTGGTGGATCCCACGGC
AAGGGATAATTCTCTTCTGATCTCACAGTTGCACTGTCTTGAGAGACATACCCTACCTTCTGA
CTCTTTTTCCTGTCTTTTTAATATACGTCTACGCCGCCATATAATTTTTTTCCTTTTTATTTCAATAA
TAATCATTATCTGGGTCGTAATTCCACTCTTCGGGACCCCCAGAATGGGACAGGGTGAGTC
GACCCCTCTCTCTCTTACACTAGATCACTGGAAAGACGTGAAGACAAGGGCTCACAATCTTT
CCGTGGAGATAAGAAAGGGAAAGTGGCAAACCTTCTGTTCCTCCGAGTGGCCCACGTTCG
AAGTGGGATGGCCACCGGAGGGGACTTTTAATCCTTCTATTATTTCTGCAGTCAAAAGGATTG
TCTTCCAGGAGACTGTTGGACCTGGTGAAAGGGTCTAGAAAGAAGAAAGATTTTTTTATTGGG
TCAATTGTATTAGTTATGTTGCTGGAAGGGCAATTTCTTTTATAATCTGGAATGTTAAATTAATTGT
GTCTTCAAAAAAAAAAAAGGGAGTCAATTTGGCTGTTGGGGCATTTTATATGATCCTGTTTTGTA

ACATTTCCTTTTAAATATTGTAATTCAAGGAAGTATAAGATTTTCAAATTTTGTCTCTATTGAAAGG
GAATTTTCAGTTGAAATTGTTTTGTTATAATTTATAAAATTTGTGAGATTGTTAAACTAAGTTTGGTT
ATTTTTAATATCAAAACCTAAGATTGTTAATTGATTACTTGTGTGTGAAAAGGAGGGGCACATGG
CAATTCCCCTTATTTTGTAAACTTGCCAGGATCCCTGGAAACTATGGGGGGGGGGGAAGGT
CATCTGCCCTGAAAAAAAAAAAAGTGCAGCTTCCATATGTTTGTTTGACATGTGGCATGGTAAG
GAAATTTTCTTAACTGAATGGGTTGTAAATTCAATAGAGCGGGAGGGTGTCAAAGTGTCCATT
GAAATTATAGACCAAGATATCTGTGTTTCTTTTACCATTAACATGACATAGCATAGTCTATGTCC
ATAAGATACCGAGATAAGAAAAGTCCCATTGTTCTGGAGAGTGTTTGGTCCCAGACATCTTGG
GCTCAGTGATAACAAACAGAAAGAATGTTGGTCAGTTCTAACTGGTCCTGGAAAGGGGTGTT
TTTGGTTCACTTGAAAAAAAAAAAAGAATTAGTTCAGGTTTTTGGCCCTTATTGGGGTTCAATTGA
TTATACTCACCTCAAGATGTTCCCATTGTTTAAAGGAATTATGAGAGCAGTAGTATTTTTCTGTG
ATCAGTCTCTTACTATTACCAATGTGAGATTAAATTCTTATTATCCAGGATTTGTGATTTTATGGG
ACAGACATTTACTATTGTTTTGAGCTTTGATTACCAGGGTACTTGTCTGAGTTGTCATAAAGCC
AATAGTAGAATGGCATGTGCTGCAGGCTGAGAACTGATAAAGGTGGAAGTCTGTGCCTGGG
AAGACTTTTGAGGACAACCTTGGACACAGCCTAGATAAGATCTTCCTGACTCTATTTCCCAAT
TGTGCTATTTCCTTTCTGACACGGGAAATCCCCACCTGATTAATCCCAGGCCCTGCTTTTAAC
ATCCAGTGACTATGCGATTACCTATCAGATATGACTCATGCCTGGAATCAAATGGAGCTAAG
GAAGTTCTTTCCTTCTGATATGATATATGACCCTGTCCCTCTGGAACATCCCTGTGTTACCGTA
ATAGGATGCAAGTACATTCATGGCCACATAATAATAATATAGGGATAACATCCCCCTAAGGAG
GAAATGTTTATACACAATAATAGGACAAACGTGTAATGTGATAGATGTCTAATTAAATAGGATAG
CAAATTTTGAGAAAAGCAATAGGATTAGACTGATATGATTGGCTTCCAAGATGTATCTATTATAG
CAATCCAAGCTTTAAGCATGTTTAGGTAGGAAGATCACAGTTTTGGATTTTTACACTAAGCCAG
AGAAGCAGAGCTCCCTTTTTGAACTGCCTATGAATTAAACCGATTGTACATGAGCAAGGAACT
TAATATTAGTTAATATAGGTAATACTGATTGTATTGATTTGTATGATTCCTGTCTAAGGTTTTCTTT
GTTACCCTGATTACATGTAAATCTCTCTTTTCCAAACTAGTCTATTGTGAGCTCTCTGATTGGTT
CAATCTGTACCTGACTTAATGTAATTATGCAATTTTGCAAATTATGAGTAAACTTTGTTGTATTGTT
TGTTTGAACCTAGTCCTGTGTGACTGGGAAAACTGAACTATCTCTATGTGCCTTTAACTAAAGA
CTTATGAGGGATTCTGGATACTTAGGAATTTAGTTTCCTTAATACTACTCTGGAATTTGTGTTATT
TAAGAACTTTTGCACCAATTTAGTGAGGTTTTATGAACAAGAGTAGAAATGCATGCAAGCTTTTT
AGGGCTCATTTTGGAAATGTTCCTTAGGCAATGTGACATCTTAGGGTACTATAGAACTGGGAC
TTGTGTTCTGTGTGTGTTCCTATATGAGTTAAAGTTCTTTACAATTAGGTCAGGGGTTTGTGAGA
ATGTTATTTTGTTATTTGGGTAATATCATACTTGTTTAAAGTTGTGTTACAATTAATAATGTTAAAAG
GGAAATGGCTTGTGGTTTATCTTCTCCATGTCATCAGAGAAACATGGCATGACTGAAACATTT
AAGATCCTATATGCACTGTGTTAAAATGGATTCTTTCATATATCTATGTATGCATTGGGGCCTGT
GGATTAATGTAAGGTATGCATATTGTTGTTTGCTCTTGTTTACTATTTAGTGGGGGTTGGCAGTA
TGGGTCCGGCTCTTACGGCAGTCCTCTTCCTTGGATGATTCCTTTGCCAAAAACTAAAAGGG
GGGAGTAGGAACTCAGGGCAGATGGCACTAATTCTTCCTTAACCAAGTTCTGCAGGGACTC
CAGAAAGAAAACACAGAGGCCACTACTGATCATACTTCTCTTGTGTTTGTTTGGTCCATGTTTA
CTAAATATTCTTGCCAGATTCCTTTCTTCTAGGGTCGGAGCCATCAACTTCCAGATGACTGTAT
ACCAACCCAAGGTCTCTAGTCCACTGAACCTGGCATCAGCCAGGTTTTGAAGTTTGACCCC
TCGAACGGGACCCAGAGAGGCAGGGACAGCTCTACGCCCCTTGCCAGCAGGAAGCAGTT
TCAGAAGCAGAGACCTCCGCCCCTTTTCACCAAAAGAATTTGGTTCCTATTTGTTTGAGGGG
GGTGATGGGGGCGGGGCCCAAACCAAGATCTTCGCTCTTTGCCTCAAAGGGAATCTGACT
CACAGGCCCCAGACCCTCATCTCCCTTTGAAAGCCTTAGCCTTACCTTTTAATCTTATTCAGA
TCTGGCTCCCTTTTGTCTATAGGCCCCAGAAAGGCCTTCCTTTATCTCCTTTTGAAAGTCCTA
GCCCCACCTTGATTTTGTGCGATGCCTTTCAAACTTCTGCCTTCCTGTTCCCTCCATTATCTAT
TCCCACTGTCTATTCAATCTCCCTTGACCCTCCCTGCATTTCCCTATTCTCCACTACCCCTCC

CTGTTTCACATGCCCCCACCTACTTGCCCATCCCCCTTTTCCTTGGTTCTCCTTCCCTTTTCC
TCTGGCTCCTAGTAGGACCCCTCAGGCAGATCCTCCTCACCCCTTGATTATGAGACTGCTC
CGAGCCATCCATAGCATTGTCAACTCCACCCGGCCTGACCTAGGACCTAGCTGCTGGCTG
TGTATGGATGCACAACCCCCATACTATGTGGGAGTGGCAGTTAATGGTTCTGTTCCCATAGC
CCAGACTCGGAAAATTGTCGGTGGGAACAACCTAAGTTGACCCTGGGGGATGTCCAAGGA
AAAGGTACCTGTCTTACCTCATCCACCACATCCCTGAAAACTTCTCCTTACTCTCCTGTCTGT
AATAGTACTGTGGTTGTTCCCCAATCCTCCTCCTCTTTTTATTCAGGGCCATCCGGCACTGGT
GGGCCTGTCTGGATGGGATCACACAGTGTGTCTCCGCTGCAGTGTTCCTAAAAGAATCTGA
GCCTCTATGTATATTAGTCTCATTATCCCCAGGGTCTCCCTTCTTCAGGGCCTGGAGGGTTG
GGAGTACTTCAGTCATGAGGGCAGTGTGTCCTACTGAAGCAAGCGAGCCGTTCCCCTCCTC
ATCCCTATCCTCAGTGGGGTTGGGGTTAGTGGGATCAACTGCTGTGGGTACCGCTGCCCTA
GTCCGAGGAGAGGCCAGTTACCAGGAACTTAGTGCTCAGGTCGATGCAGACCTCAGTCAG
TCATCTGGAGCACTCTACATCCCAACTGGAGAGACAGGTCAACTCCTTGGCAGAGATGGTT
CTGCAGAACCAGAGGGGTTTAGGCTTATTGTTTCTCAGGCAAGGTGGCCTTTGTGCCGCCC
TAGGGGAGGCCTGCTGTTTCTATGCCAATAACTCAGGTGTAGTTCAGGAGAGCCTTTCCCTG
GCACGGAAAAATATTGCAGACAGGCAGAGGGAACGAGAAGGGAACGAAAATTGGTACCAC
AGCCTGTTTTGGACTTCCCCATGGTTAACCATTCTTATTTCTGCCTTAGCCGGGTCTTTGCTC
CTCCTTACTATAGCCCTAATTCTTGGTCCTTGTCTAGTTAATCGGCTCCTTGACTTTGTTAGATC
CTGTATTAATTTAGTTAAGTTACTGTTGGTTAAAGGTCCCCGTTATCAGACCCTGGTGTCTGCC
TTCTTTGCACCCTATAATGAGGCAGCCCCAACCACGTCAAGGGTTTGACGTGGTACACAAAA
GAAGTGGGGAATGTAATGCAGAACATTATTTTACCATTTGGGTAGTAGAACCCTTTAGGCCAT
GGAAAACTCCAAATTCCTTAGCAAGACCCTTATCTGGCTGCTCAGGCCACAGCCCTCAGAT
CATATCTCTGGAAGCCTCCATTGTGAGAAAGTTTGCAGAGCATTATCATATCTACACAGAGGG
AGAAGGGTGTCCTTGACCAGCCCCCCCCAAAAAAACCTCTTCCTCTTGCCAGACTTCAAAG
ATCCATCCCCCTCCCTGTCCTGTATACTCCCTATATAAACCCTAGCTCAACCCCTGATGGG
GGCTGCTTGATTTGGATGTGAATATCCCTGGCAGCCGCGCGCTAATAGCTAATAAAGTCCAC
AATTTAAAATTAGCTTGGGTCGTCAACTCGCCTATAGGGATGAGAGTGGCCCCAGGAGACTC
AAGGAAAGGTTAGATAAGAGGCAGTTAGAGCACCAAAAGAATTTAAGTTGGTACGAGGGATG
GTTCAACCGTTCCCCCTGGCTTACTACTTTACTGTCTGCCCTTGCTGGTCCCCTGCTACTCC
TCCTTCTGTTACTCACCCTCGGGCCTTGTGTCATCAATAAGTTAGTGCAATTCATCAATGATAG
GGTTAGTGCAGTAAGGATTCTGGTTCTCAGGCACAAGTACCAGACCCTAGACAACGAGGAT
AACCTTTAATTCTGCTCTATGATTAGAGCTACCCTCAAAGAAAATGGGGGAATGAAGGAGGC
AGAAATCATGAGGCAGAAATCATTCCGTGGAGTATGGAAACTACCCGGAGGGCCCAAGGTT
TAGGGACAGGTGCAGCCAGGCACAGTAAAAGGTCAGAGCAAGAAAAACAAGGAAGATTTG
GAGTGCCAAACGGGATATCTGTGGTCATGCACCTGAGTCCCCACCCCGGACTTATGCAAA
CAATTCCCAGAAATAGCTGAGCTCATAACAGTTTCTAGGGTGCCCCTCAGCAGTTTCTAGAA
CCCTCTCGTGACCGGAGTTTTTATTCAAACTAACCAATGATCTTGCTCCTCGCTTCTGTACCC
GCGCTTTTTTGCTATAAAATGAGACCAAAGAATCCACCCGGCGCGCCAGTCCCTCTAGGTG
ACTGAGTCGCCCGAGTACTCGTAAGTTCAATAAACCTCTTGCTATTTGCATCCGGAGTTGTGT
TCGCGTTGATCCTGGGAGGGTTTCTCAAGGTCGGAGGACTACCCGAACATTGGGGTCTTTC
AG
>RecKoRV1-11
TTTGAAGGAGGCAGAAATCATGAGGCAGAAATCATTCCGTGGAGTATGGAAACTACCCGGA
GGGCCCAAGGTTTAGGGACAGGTGCAGCCAGGCACAGTAAAAGGTCAGAGCAAGAAAAA
CAAGGAAGATTTGGAGTGCCAAACGGGATATCTGTGGTCATGCACCTGAGTCCCCACCCC
GGACTTATGCAAACAATTCCCAGAAATAGCTGAGCTCATAACAGTTTCTAGGGTGCCCCTCA
GCAGTTTCTAGAACCCTCTCGTGACCGGAGTTTTTATTCAAACTAACCAATGATCTTGCTCCT

CGCTTCTGTACCCGCGCTTTTTTGCTATAAAATGAGACCAAAGAATCCACCCGGCGCGCCA
GTCCCTCTAGGTGACTGAGTCGCCCGAGTACTCGTAAGTTCAATAAACCTCTTGCTATTTGC
ATCCGGAGTTGTGTTCGCGTTGATCCTGGGAGGGTTTCTCAAGGTCGGAGGACTACCCGAA
CATTGGGGTCTTTCATTTGGGGGCTCGTCCGGGATCTGAGATTCCCACCCAAGGACCGCC
GAACCACCGACGGGAGGTAAGCTGGCCAGCGATCGCTCTATGTTCTCTCTGTGTCTGACTC
CGAAAACTCTGACTGTCTATTCGGTGTGCGCGCATTTTTGGTTTCAGTTTATTCCGGGTTAATC
GATCTAAGATCGAGGCGTGAGTAGCGGACAGACGTGTTCGGGGGCTCACCGCCCGGCAA
TCCTGGGAGACGTCCCAGGATCAGGGGAGGACCAGGGACGCCTGGTGGATCCCACGGC
AAGGGATAATTCTCTTCTGATCTCACAGTTGCACTGTCTTGAGAGACATACCCTACCTTCTGA
CTCTTTTTCCTGTCTTTTTAATATACGTCTACGCCGCCATATAATTTTTTTCCTTTTTATTTCAATAA
TAATCATTATCTGGGTCGTAATTCCACTCTTCGGGACCCCCAGAATGGGACAGGGTGAGTC
GACCCCTCTCTCTTACACTAGATCACTGGAAAGACGTGAAGACAAGGGCTCACAATCTTTCC
GTGGAGATAAGAAAGGGAAAGTGGCAAACCTTCTGTTCCTCCGAGTGGCCCACGTTCGAAG
TGGGATGGCCACCGGAGGGGACTTTTAATCCTTCTATTATTTCTGCAGTCAAAAGGATTGTCT
TCCAGGAGACTGTTGGACCTGGTGAAAGGGTCTAGAAAGAAGAAAGATTTTTTTATTGGGTCA
ATTGTATTAGTTATGTTGCTGGAAGGGCAATTTCTTTTATAATCTGGAATGTTAAATTAATTGTGT
CTTCAAAAAAAAAAAAGGGAGTCAATTTGGCTGTTGGGGCATTTTATATGATCCTGTTTTGTAA
CATTTCCTTTTAAATATTGTAATTCAAGGAAGTATAAGATTTTCAAATTTTGTCTCTATTGAAAGG
GAATTTTCAGTTGAAATTGTTTTGTTATAATTTATAAAATTTGTGAGATTGTTAACTAAGTTTTGGG
GTTATTGTTTTAATATCAAAACCTAAGATTGTTAATTGATTACTTGTGTGTGAAAAGGAGGGGCA
CATGGCAATTCCCCTTATTTTGTAAACTTGCCAGGATCCCTGGAAACTATGGGGGGGGGGAA
AGGTCATCTGCCCTGAAAAAAAAAAAAGTGCAGCTTCCATATGTTTGTTTGACATGTGGCATGG
TAAGGAAATTTTCTTAACTGAATGGGTTGTAAATTCAATAGAGCGGGAGGGTGTCAAAGTGTC
CATTGAAATTATAGACCAAGATATCTGTGTTTCTTTTACCATTAACATGACATAGCATAGTCTAT
GTCCATAAGATACCGAGATAAGAAAAGTCCCATTGTTCTGGAGAGTGTTTGGTCCCAGACAT
CTTGGGCTCAGTGATAACAAACAGAAAGAATGTTGGTCAGTTCTAACTGGTCCTGGAAAGGG
GTGTTTTTGGTTCACTTGAAAAAAAAAAAAAGAATTAGTTCAGGTTTTTGGCCCTTATTGGGGTTC
AATTGATTATACTCACCTCAAGATGTTCCCATTGTTTAAAGGAATTATGAGAGCAGTAGTATTTT
TCTGTGATCAGTCTCTTACTATTACCAATGTGAGATTAAATTCTTATTATCCAGGATTTGTGATTT
TATGGGACAGACATTTACTATTGTTTTGAGCTTTGATTACCAGGGTACTTGTCTGAGTTGTCATA
AAGCCAATAGTAGAATGGCATGTGCTGCAGGCTGAGAACTGATAAAGGTGGAAGTCTGTGC
CTGGGAAGACTTTTGAGGACAACCTTGGACACAGCCTAGATAGATCTTCCTGACTCTATTTC
CCAATTGTGCTATTTCCTTTCTGACACGGGAAATCCCCACCTGATTAATCCCAGGCCCTGCT
TTTAACATCCAGTGACTATGCGATTACCTATCAGATATGACTCATGCCTGGAATCAAATGGAG
CTAAGGAAGTTCTTTCCTTCTGATATGATATATGACCCTGTCCCTCTGGAACATCCCTGTGTTA
CCGTAATAGGATGCAAGTACATTCATGGCCACATAATAATAATATAGGGATAACATCCCCCTA
AGGAGGAAATGTTTATACACAATAATAGGACAAACGTGTAATGTGATAGATGTCTAATTAAATA
GGATAGCAAATTTTGAGAAAAGCAATAGGATTAGACTGATATGATTGGCTTCCAAGATGTATCT
ATTATAGCAATCCAAGCTTTAAGCATGTTTAGGTAGGAAGATCACAGTTTTGGATTTTTACACTA
AGCCAGAGAAGCAGAGCTCCCTTTTTGAACTGCCTATGATTAAACCGATTGTACATGAGCAA
GGAACTTAATATTAGTTAATATAGGTAATACTGATTGTATTGATTTGTATGATTCCTGTCTAAGGT
TTTCTTTGTTACCCTGATTACATGTAAATCTCTCTTTTCCAAACTAGTCTATTGTGAGCTCTCTGA
TTGGTTCAATCTGTACCTGACTTAATGTAATTATGCAATTTTGCAAATTATGAGTAAACTTTGTTG
TATTGTTTGTTTGAACCTAGTCCTGTGTGACTGGGAAAACTGAACTATCTCTATGTGCCTTTAA
CTAAAGACTTATGAGGGATTCTGGATACTTAGGAATTTAGTTTCCTTAATACTACTCTGGAATTT
GTGTTATTTAAGAACTTTTGCACCAATTTAGTGAGGTTTTATGAACAAGAGTAGAAATGCATGCA
AGCTTTTTAGGGCTCATTTTGGAAATGTTCCTTAGGCAATGTGACATCTTAGGGTACTATAGAA

CTGGGACTTGTGTTCTGTGTGTGTTCCTATATGAGTTAAAGTTCTTTACAATTAGGTCAGGGGTT
TGTGAGAATGTTATTTTGTTATTTGGGTAATATCATACTTGTTTAAAGTTGTGTTACAATTAATAAT
GTTAAAAGGGAAATGGCTTGTGGTTTATCTTCTCCATGTCATCAGAGAAACATGGCATGACTG
AAACATTTAAGATCCTATATGCACTGTGTTAAAATGGATTCTTTCATATATCTATGTATGCATTGG
GGCCTGTGGATTAATGTAAGGTATGCATATTGTTGTTTGCTCTTGTTTACTATTTAGTGGGGGGT
TGGCAGTATGGGTCCGGCTCTTACGGCAGTCCTCTTCCTTGGATGATTCCTTTGCCAAAAAC
TAAAAGGGGGAGTAGGAACTCAGGGCGGATGGCACTAATTCTTCCTTAACCAAGTTCTGCA
GGGACTCCAGAAAGAAAACACAGAGGCCACTACTGATCATACTTCTCTTGTGTTTGTTTGGTC
CATGTTTACTAAATATTCTTGCCAGATTCCTTTCTTCTAGGGTCGGAGCCATCAACTTCCAGAT
GACTGTATACCAACCCAAGGTCTCTAGTCCACTGAACCTGGCATCAGCCAGGTTTTGAAGTT
TGACCCCTCGAACGGGACCCAGAGAGGCAGGGACAGCTCTACGCCCCTTGCCAGCAGG
AAGCAGTTTCAGAAGCAGAGACCTCCGCCCCTTTTCACCAAAAGAATTTGGTTCCTATTTGTT
TGAGGGGGGATGATGGGGGCGGGGCCCAAACCAAGATCTTCGCTCTTTGCCTCAAAGGGA
ATCTGACTCACAGGCCCCAGACCCTCATCTCCCTTTGAAAGCCTTAGCCTTACCTTTTAATCT
TATTCAGATCTGGCTCCCTTTTGTCTATAGGCCCCAGAAAGGCCTTCCTTTATCTCCTTTTGAA
AGTCCTAGCCCCACCTTGATTTTGTGGATGCCTTTCAAACTTCTGCCTTCCTGTTCCCTCCAT
TATCTATTCCCACTGTCTATTCAATCTCCCTTGACCCCCCTGCATTTCCCTATTCTCCACTAC
CCCTCCCTGTTTCACATGCCCCCACCTACTTGCCCATCCCCCCTTTTCCTTGGTTCTCCTTC
CCTTTTCCTCTGGCTCCTAGTAGGACCCCTCAGGCAGATCCTCCTCACCCCTGATTATGAG
ACTGCTCCGAGCCATCCATAGCATTGTCAACTCCACCCGGCCTGACCTAGGACCTAGCTG
CTGGCTGTGTATGGATGCACAACCCCCATACTATGTGGGAGTGGCAGTTAATGGTTCTGTTC
CCATAGCCCAGACTCAGAGAATTGTCGGTGGGAACAACCTAAGTTGACCCTGGGGGATGT
CCAAGGAAAAGGTACCTGTCTTACCTCATCCACCACATCCCTGAAAACTTCTCCTTACTCTC
CTGTCTGTAATAGTACTGTGGTTGTTCCCCAATCCTCCTCCTCTTTTTATTCAGGGCCATCCG
GCACTTGGTGGGCCTGTCTGGATGGGATCACACAGTGTGTCTCCGCTGCAGTGTTCCTAAA
AGAATCTGAGCCTCTATGTATATTAGTCTCCATTATCCCCAGGGTCTCCCTTCTTCAGGGCCT
GGAGGGTTGGGAGTACTTCAGTCATGAGGGCAGTGTGTCCTACTGAAGCAAGCGAGCCGTT
CCCCTCCTCATCCCTATCCTCAGTGGGGTTGGGGTTAGTGGGATCAACTGCTGTGGGTACC
GCTGCCCTAGTCCGAGGAGAGGCCAGTTACCAGGAACTTAGTGCTCAGGTCGATGCAGAC
CTCAGTCAGTCATCTGGAGCACTCTACATCCCAACTGGAGAGACAGGTCAACTCCTTGGCA
GAGATGGTTCTGCAGAACCAGAGGGGTTTAGGCTTATTGTTTCTCAGGCAAGGTGGCCTTTG
TGCCGCCCTAGGGGAGGCCTGCTGTTTCTATGCCAATAACTCAGGTGTAGTTCAGGAGAGC
CTTTCCCTGGCACGGAAAAATATTGCAGACAGGCAGAGGGAACGAGAAGGGAACGAAAATT
GGTACCACAGCCTGTTTGGACTTCCCCATGGTTAACCATTCTTATTTCTGCCTTAGCCGGGT
CTTTGCTCCTCCTTACTATAGCCCTAATTCTTGGTCCTTGTCTAGTTAATCGGCTCCTTGACTTT
GTTAGATCCTGTATTAATTTAGTTAAGTTACTGTTGGTTAAAGGTCCCCGTTATCAGACCCTGG
TGTCTGCCTTCTTTGCACCCTATAATGAGGCAGCCCCAACCACGTCAAGGGTTTGACATGGT
ACACAAAAGAAGTGGGGAATGTAATGCAGAACATTATTTTACCATTGGGTAGTAGAACCCTTT
AGGCCATGGAAAACTCCAAATTCCTTAGCAAGACCCTTATCTGGCTGCTCAGGCCACAGCC
CTCAGATCATATCTCTGGAAGCCTCCATTGTGAGAAAGTTTGCAGAGCATTATCATATCTACA
CAGAGGGAGAAGGGTGTCCTTGACCAGCCCCCCCCAAAAAAACCTCTTCCTCTTGCCAGA
CTTCAAAGATCCATCCCCCTCCCTGTCCTGTATACTCCCTATATAAACCCTAGCTCAACCCC
TGATGGGGGCTGCTTGATTTGGATGTGAATATCCCTGGCAGCCGCGCGCTAATAGCTAATAA
AGTCCACAATTTAAAATTAGCTTGGGTCGTCAACTCGCCTATAGGGATGAGAGTGGCCCCAG
GAGACTCAAGGAAAGGTTAGATAAGAGGCAGTTAGAGCACCAAAAGAATTTAAGTTGGTACG
AGGGATGGTTCAACCGTTCCCCCTGGCTTACTACTTTACTGTCTGCCCTTGCTGGTCCCCTG
CTACTCCTCCTTCTGTTACTCACCCTCGGGCCTTGTGTCATCAATAAGTTAGTGCAATTCATC

AATGATAGGGTTAGTGCAGTAAGGATTCTGGTTCTCAGGCACAAGTACCAGACCCTAGACAA
CGAGGATAACCTTTAATTCTGCTCTATGATTAGAGCTACCCTCAAAGAAAATGGGGGAATGAA
GGAGGCAGAAATCATGAGGCAGAAATCATTCCGTGGAGTATGGAAACTACCCGGAGGGCC
CAAGGTTTAGGGACAGGTGCAGCCAGGCACAGTAAAAGGTCAGAGCAAGAAAAACAAGGA
AGATTTGGAGTGCCAAACGGGATATCTGTGGTCATGCACCTGAGTCCCCACCCCGGACTTA
TGCAAACAATTCCCAGAAATAGCTGAGCTCATAACAGTTTCTAGGGTGCCCCTCAGCAGTTT
CTAGAACCCTCTCGTGACCGGAGTTTTTATTCAAACTAACCAATGATCTTGCTCCTCGCTTCT
GTACCCGCGCTTTTTTGCTATAAAATGAGACCAAAGAATCCACCCGGCGCGCCAGTCCCTC
TAGGTGACTGAGTCGCCCGAGTACTCGTAAGTTCAATAAACCTCTTGCTATTTGCATCCGGA
GTTGTGTTCGCGTTGATCCTGGGAGGGTTTCTCAAGGTCGGAGGACTACCCGAACATTGGG
GTCTTTCAG
>RecKoRV1-30
TTTGAAGGAGGCAGAAATCATGAGGCAGAAATCATTCCGTGGAGTATGGAAACTACCCGGA
GGGCCCAAGGTTTAGGGACAGGTGCAGCCAGGCACAGTAAAAGGTCAGAGCAAGAAAAA
CAAGGAAGATTTGGAGTGCCAAACGGGATATCTGTGGTCATGCACCTGAGTCCCCACCCC
GGACTTATGCAAACAATTCCCAGAAATAGCTGAGCTCATAACAGTTTCTAGGGTGCCCCTCA
GCAGTTTCTAGAACCCTCTCGTGACCGGAGTTTTTATTCAAACTAACTAATGATCTTGCTCCTC
GCTTCTGTACCCGCGCTTTTTTGCTATAAAATGAGACCAAAGAATCCACCCGGCGCGCCAG
TCCCTCTAGGTGACTGAGTCGCCCGAGTACTCGTAAGTTCAATAAACCTCTTGCTATTTGCAT
CCGGAGTTGTGTTCGCGTTGATCCTGGGAGGGTTTCTCAAGGTCGGAGGACTACCCGAACA
TTGGGGTCTTTCATTTGGGGGCTCGTCCGGGATCTGAGATTCCCACCCAAGGACCGCCGA
ACCACCGACGGGAGGTAAGCTGGCCAGCGATCGCTCTATGTTCTCTCTGTGTCTGACTCCG
AAAACTCTGACTGTCTATTCGGTGTGCGCGCATTTTTGGTTTCAGTTTATTCCGGGTTAATCGA
TCTAAGATCGAGGCGTGAGTAGCGGACAGACGTGTTCGGGGGCTCACCGCCCGGCAATC
CTGGGAGACGTCCCAGGATCAGGGGAGGACCAGGGACGCCTGGTGGATCCCACGGCAA
GGGATAATTCTCTTCTGATCTCACAGTTGCACTGTCTTGAGAGACATACCCTACCTTCTGACT
CTTTTTCCTGTCTTTTTAATATACGTCTACGCCGCCATATAATTTTTTTCCTTTTTATTTCAATAATA
ATCATTATCTGGGTCGTAATTCCACTCTTCGGGACCCCCAGAATGGGACAGGGTGAGTCGA
CCCCTCTCTCTCTTACACTAGATCACTGGAAAGACGTGAAGACAAGGGCTCACAATCTTTCC
GTGGAGATAAGAAAGGGAAAGTGGCAAACCTTCTGTTCCTCCGAGTGGCCCACGTTCGAAG
TGGGATGGCCACCGGAGGGGACTTTTAATCCTTCTATTATTTCTGCAGTCAAAAGGATTGTCT
TCCAGGAGACTGTTGGACCTGGTGAAAGGGTCTAGAAAGAAGAAAGATTTTTTTATTGGGTCA
ATTGTATTAGTTATGTTGCTGGAAGGGCAATTTCTTTTATAATCTGGAATGTTAAATTAATTGTGT
CTTCAAAAAAAAAAAGGGAGTCAATTTGGCTGTTGGGGCATTTTATATGATCCTGTTTTGTAAAA
TTTCCTTTTAAATATTGTAATTCAAGGAAGTATAAGATTTTCAAATTTTGTCTCTATTGAAAGGGAA
TTTTCAGTTGAAATTGTTTTGTTATAATTTATAAAATTTGTGAGATTGTTAACTAAGTTTTGGGGTTA
TTGTTTTAATATCAAAACCTAAGATTGTTAATTGATTACTTGTGTGTGAAAAGGAGGGGCACATG
GCAATTCCCCTTATTTTGTAAACTTGCCAGGATCCCTGGAAACTATGGGGGGGGGGGAAGG
TCATCTGCCCTGAAAAAAAAAAAGTGCAGCTTCCATATGTTTGTTTGACATGTGGCATGGTAA
GGAAATTTTCTTAACTGAATGGGTTGTAAATTCAATAGAGCGGGAGGGTGTCAAAGTGTCCAT
TGAAATTATAGACCAAGATATCTGTGTTTCTTTTACCATTAACATGACATAGCATAGTCTATGTC
CATAAGATACCGAGATAAGAAAAGTCCCATTGTTCTGGAGAGTGTTTGGTCCCAGACATCTTG
GGCTCAGTGATAACAAACAGAAAGAATGTTGGTCAGTTCTAACTGGTCCTGGAAAGGGGTGT
TTTTGGTTCACTTGAAAAAAAAAAAGAATTAGTTCAGGTTTTTGGCCCTTATTGGGGTTCAATTG
ATTATACTCACCTCAAGATGTTCCCATTGTTTAAAGGAATTATGAGAGCAGTAGTATTTTTCTGT
GATCAGTCTCTTACTATTACCAATGTGAGATTAAATTCTTATTATCCAGGATTTGTGATTTTATGG
GACAGACATTTACTATTGTTTTGAGCTTTGATTACCAGGGTACTTGTCTGAGTTGTCATAAAGC

CAATAGTAGAATGGCATGTGCTGCAGGCTGAGAACTGATAAAGGTGGAAGTCTGTGCCTGG
GAAGACTTTTGAGGACAACCTTGGACACAGCCTAGATAGATCTTCCTGACTCTATTTCCCAAT
TGTGCTATTTCCTTTCTGACACGGGAAATCCCCACCTGATTAATCCCAGGCCCTGCTTTTAAC
ATCCAGTGACTATGCGATTACCTATCAGATATGACTCATGCCTGGAATCAAATGGAGCTAAG
GAAGTTCTTTCCTTCTGATATGATATATGACCCTGTCCCTCTGGAACATCCCTGTGTTACCGTA
ATAGGATGCAAGTACATTCATGGCCACATAATAATAATATAGGGATAACATCCCCCTAAGGAG
GAAATGTTTATACACAATAATAGGACAAACGTGTAATGTGATAGATGTCTAATTAAATAGGATAG
CAAATTTTGAGAAAAGCAATAGGATTAGACTGATATGATTGGCTTCCAAGATGTATCTATTATAG
CAATCCAAGCTTTAAGCATGTTTAGGTAGGAAGATCACAGTTTTGGATTTTTACACTAAGCCAG
AGAAGCAGAGCTCCCTTTTTGAACTGCCTATGAATTAAACCGATTGTACATGAGCAAGGAACT
TAATATTAGTTAATATAGGTAATACTGATTGTATTGATTTGTATGATTCCTGTCTAAGGTTTTCTTT
GTTACCCTGATTACATGTAAATCTCTCTTTTCCAAACTAGTCTATTGTGAGCTCTCTGATTGGTT
CAATCTGTACCTGACTTAATGTAATTATGCAATTTTGCAAATTATGAGTAAACTTTGTTGTATTGTT
TGAACCTAGTCCTGTGTGACTGGGAAAACTGAACTATCTCTATGTGCCTTTAACTAAAGACTTA
TGAGGGATTCTGGAATACTTAGGAATTTAGTTTCCTTAATACTACTCTGGAATTTGTGTTATTTAA
GAACTTTTGCACCAATTTAGTGAGGTTTTATGAACAAGAGTAGAAATGCATGCAAGCTTTTTAG
GGCTCATTTTGGAAATGTTCCTTAGGCAATGTGACATCTTAGGGTACTATAGAACTGGGACTT
GTGTTCTGTGTGTGTTCCTATATGAGTTAAGTTCTTTACAATTAGGTCAGGGGTTTGTGAGAATG
TTATTTTGTTATTTGGGTAATATCATACTTGTTTAAAGTTGTGTTACAATTAATAATGTTAAAAGGG
AAATGGCTTGTGGTTTATCTTCTCCATGTCATCAGAGAAACATGGCATGACTGAAACATTTAAG
ATCCTATATGCACTGTGTTAAAATGGATTCTTTCATATATCTATGTATGCATTGGGGCCTGTGGA
TTAATGTAAGGTATGCATATTGTTGTTTGCTCTTGTTTACTATTTAGTGGGGGGTTGGCAGTATG
GGTCCGGCTCTTACGGCAGTCCTCTTCCTTGGATGATTCCTTTGCCAAAAACTAAAAGGGGG
GAGTAGGAACTCAGGGCAGATGGCACTAATTCTTCCTTAACCAAGTTCTGCAGGGACTCCA
GAAAGAAAACACAGAGGCCACTACTGATCATACTTCTCTTGTGTTTGTTTGGTCCATGTTTACT
AAATATTCTTGCCAGATTCCTTTCTTCTAGGGTCGGAGCCATCAACTTCCAGATGACTGTATA
CCAACCCAAGGTCTCTAGTCCACTGAACCTGGCATCAGCCAGGTTTTGAAGTTTGACCCCT
CGAACGGGACCCAGAGAGGCAGGGACAGCTCTACGCCCCTTGCCAGCAGGAAGCAGTTT
CAGAAGCAGAGACCTCCGCCCCTTTTCACCAAAAGAATTTGGTTCCTATTTGTTTGAGGGGG
GGATGATGGGGGCGGGGCCCAAACCAAGATCTTCGCTCTTTGCCTCAAAGGGAATCTGAC
TCACAGGCCCCAGACCCTCATCTCCCTTTGAAAGCCTTAGCCTTACCTTTTAATCTTATTCAG
ATCTGGCTCCCTTTTGTCTATAGGCCCCAGAAAGGCCTTCCTTTATCTCCTTTTGAAAGTCCT
AGCCCCACCTTGATTTTGTGCGATGCCTTTCAAACTTCTGCCTTCCTGTTCCCTCCATTATCTA
TTCCCACTGTCTATTCAATCTCCCTTGACCCTCCCTGCATTTCCCTATTCTCCACTACCCCTC
CCTGTTTCACATGCCCCCACCTACTTGCCCATCCCCCCTTTTCCTTGGTTCTCCTTCCCTTTT
CCTCTGGCTCCTAGTAGGACCCCTCAGGCAGATCCTCCTCACCCCTTGATTATGAGACTGC
TCCGAGCCATCCATAGCATTGTCAACTCCACCCGGCCTGACCTAGGACCTAGCTGCTGGC
TGTGTATGGATGCACAACCCCCATACTATGTGGGAGTGGCAGTTAATGGTTCTGTTCCCATA
GCCCAGACTCGGAAAATTGTCGGTGGGAACAACCTAAGTTGACCCTGGGGGATGTCCAAG
GAAAAGGTACCTGTCTTACCTCATCCACCACATCCCTGAAAACTTCTCCTTACTCTCCTGTCT
GTAATAGTACTGTGGTTGTTCCCCAATCCTCCTCCTCTTTTTATTCAGGGCCATCCGGCACTG
GTGGGCCTGTCTGGATGGGATCACACAGTGTGTCTCCGCTGCAGTGTTCCTAAAAGAATCT
GAGCCTCTATGTATATTAGTCTCATTATCCCCAGGGTCTCCCTTCTTCAGGGCCTGGAGGGT
TGGGAGTACTTCAGTCATGAGGGCAGTGTGTCCTACTGAAGCAAGCGAGCCGTTCCCCTC
CTCATCCCTATCCTCAGTGGGGTTGGGGTTAGTGGGATCAACTGCTGTGGGTACCGCTGCC
CTAGTCCGAGGAGAGGCCAGTTACCAGGAACTTAGTGCTCAGGTCGATGCAGACCTCAGT
CAGTCATCTGGAGCACTCTACCCAACTGGAGAGACAGGTCAACTCCTTGGCAGAGATGGTT

CTGCAGAACCAGAGGGGTTTAGGCTTATTGTTTCTCAGGCAAGGTGGCCTTTGTGCCGCCC
TAGGGGAGGCCTGCTGTTTCTATGCCAATAACTCAGGTGTAGTTCAGGAGAGCCTTTCCCTG
GCACGGAAAAATATTGCAGACAGGCAGAGGGAACGAGAAGGGAACGAAAATTGGTACCAC
AGCCTGTTTTGGACTTCCCCATGGTTAACCATTCTTATTTCTGCCTTAGCCGGGTCTTTGCTC
CTCCTTACTATAGCCCTAATTCTTGGTCCTTGTCTAGTTAATCGGCTCCTTGACTTTGTTAGATC
CTGTATTAATTTAGTTAAGTTACTGTTGGTTAAAGGTCCCCGTTATCAGACCCTGGTGTCTGCC
TTCTTTGCACCCTATAATGAGGCAGCCCCCACCATGTCAAGGGTTTGACATGGTACACAAAA
GAAGTGGGGAATGTAATGCAGAACATTATTTTACCATTTGGTAGTAGAACCCTTTAGGCCATG
GAAAACTCCAAATTCCTTAGCAAGACCCTTATCTGGCTGCTCAGGCCACAGCCCTCAGATC
ATATCTCTGGAAGCCTCCATTGTGAGAAAGTTTGCAGAGCATTATCATATCTACACAGAGGGA
GAAGGGTGTCCTTGACCAGCCCCCCCAAAAAAACCTCTTCCTCTTGCCAGACTTCAAAGAT
CCATCCCCCTCCCTGTCCTGTATACTCCCTATATAAACCCTAGCTCAACCCCTGATGGGGG
CTGCTTGATTTGGATGTGAATATCCCTGGCAGCCGCGCGCTAATAGCTAATAAAGTCCACAA
TTTAAAATTAGCTTGGGTCGTCAACTCGCCTATAGGGATGAGAGTGGCCCCAGGAGACTCAA
GGAAAGGTTAGATAAGAGGCAGTTAGAGCACCAAAAGAATTTAAGTTGGTACGAGGGATGGT
TCAACCGTTCCCCCTGGCTTACTACTTTACTGTCTGCCCTTGCTGGTCCCCTGCTACTCCTC
CTTCTGTTACTCACCCTCGGGCCTTGTGTCATCAATAAGTTAGTGCAATTCATCAATGATAGG
GTTAGTGCAGTAAGGATTCTGGTTCTCAGGCACAAGTACCAGACCCTAGACAACGAGGATA
ACCTTTAATTCTGCTCTATGATTAGAGCTACCCTCAAAGAAAATGGGGGAATGAAGGAGGCA
GAAATCATGAGGCAGAAATCATTCCGTGGAGTATGGAAACTACCCGGAGGGCCCAAGGTTT
AGGGACAGGTGCAGCCAGGCACAGTAAAAGGTCAGAGCAAGAAAAACAAGGAAGATTTGG
AGTGCCAAACGGGATATCTGTGGTCATGCACCTGAGTCCCCACCCCGGACTTATGCAAACA
ATTCCCAGAAATAGCTGAGCTCATAACAGTTTCTAGGGTGCCCCTCAGCAGTTTCTAGAACC
CTCTCGTGACCGGAGTTTTTATTCAAACTAACCAATGATCTTGCTCCTCGCTTCTGTACCCGC
GCTTTTTTGCTATAAAATGAGACCAAAGAATCCACCCGGCGCGCCAGTCCCTCTAGGTGACT
GAGTCGCCCGAGTACTCGTAAGTTCAATAAACCTCTTGCTATTTGCATCCGGAGTTGTGTTCG
CGTTGATCCTGGGAGGGTTTCTCAAGGTCGGAGGACTACCCGAACATTGGGGTCTTTCAC
>RecKoRV2-01
AATGAAGGAGGCAGAAATCATGAGGCAGAAATCATTCCGTGGAGTATGGAAACTACCCGGA
GGGCCCAAGGTTTAGGGACAGGTGCAGCCAGGCACAGTAAAAGGTCAGAGCAAGAAAAA
CAAGGAAGATTTGGAGTGCCAAACGGGATATCTGTGGTCATGCACCTGAGTCCCCACCCC
GGACTTATGCAAACAATTCCCAGAAATAGCTGAGCTCATAACAGTTTCTAGGGTGCCCCTCA
GCAGTTTCTAGAACCCTCTCGTGACCGGAGTTTTTATTCAAACTAACCAATGATCTTGCTCCT
CGCTTCTGTACCCGCGCTTTTTTGCTATAAAATGAGACCAAAGAATCCACCCGGCGCGCCA
GTCCCTCTAGGTGACTGAGTCGCCCGAGTACTCGTAAGTTCAATAAACCTCTTGCTATTTGC
ATCCGGAGTTGTGTTCGCGTTGATCCTGGGAGGGTTTCTCAAGGTCGGAGGACTACCCGAA
CATTGGGGTCTTTCATTTGGGGGCTCGTCCGGGATCTGAGATTCCCACCCAAGGACCGCC
GAACCACCGACGGGAGGTAAGCTGGCCAGCGATCGCTCTATGTTCTCTCTGTGTCTGACTC
CGAAAACTCTGACTGTCTATTCGGTGTGCGCGCATTTTTGGTTTCAGTTTATTCCGGGTTAATC
GATCTAAGATCGAGGCGTGAGTAGCGGACAGACGTGTTCGGGGGCTCACCGCCCGGCAA
TCCTGGGAGACGTCCCAGGATCAGGGGAGGACCAGGGACGCCTGGTGGATCCCACGGC
AAGGGATAATTCTCTTCTGATCTCACAGTTGCACTGTCTTGAGAGACATACCCTACCTTCTGA
CTCTTTTTCCTGTCTTTTTAATATACGTCTACGCCGCCATATAATTTTTTTCCTTTTTATTTCAATAA
TAATCATTATCTGGGTCGTAATTCCACTCTTCGGGACCCCCAGAATGGGACAGGGTGAGTC
GACCCCTCTCTCTCTTACACTAGATCACTGGAAAGACGTGAAGACAAGGGCTCACAATCTTT
CCGTGGAGATAAGAAAGGGAAAGTGGCAAACCTTCTGTTCCTCCGAGTGGCCCACGTTCG
AAGTGGGATGGCCACCGGAGGGGACTTTTAATCCTTCTATTATTTCTGCAGTCAAAAGGATTG

TCTTCCAGGAGACTGTTGGACCTGGTGAAAGGGTCTAGAAAGAAGAAAGATTTTTTTATTGGG
TCAATTGTATTAGTTATGTTGCTGGAAGGGCAATTTCTTTTATAATCTGGAATGTTAAATTAATTGT
GTCTTCAAAAAAAAAAAAGGGAGTCAATTTGGCTGTTGGGGCATTTTATATGATCCTGTTTTGTAA
CATTTCCTTTTAAATATTGTAATTCAAGGAAGTATAAGATTTTCAAATTTTGTCTCTATTGAAAGG
GAATTTTCAGTTGAAATTGTTTTGTTATAATTTATAAAATTTGTGAGATTGTTAACTAAGTTTTGGG
GTTATTGTTTTAATATCAAAACCTAAGATTGTTAATTGATTACTTGTGTGTGAAAAGGAGGGGCA
CATGGCAATTCCCCTTATTTTGTAAACTTGCCAGGATCCCTGGAAACTATGGGGGGGGGGG
GAGAAAGGTCATCTGCCCTGAAAAAAAAAAAAGTGCAGCTTCCATATGTTTGTTTGACATGTGG
CATGGTAAGGAAATTTTCTTAACTGAATGGGTTGTAAATTCAATAGAGCGGGAGGGTGTCAAA
GTGTCCATTGAAATTATAGACCAAGATATCTGTGTTTCTTTTACCATTAACATGACATAGCATAG
TCTATGTCCATAAGATACCGAGATAAGAAAAGTCCCATTGTTCTGGAGAGTGTTTGGTCCCAG
ACATCTTGGGCTCAGTGATAACAAACAGAAAGAATGTTGGTCAGTTCTAACTGGTCCTGGAAA
GGGGTGTTTTTGGTTCACTTGAAAAAAAAAAAAAGAATTAGTTCAGGTTTTTGGCCCTTATTGGG
GTTCAATTGATTATACTCACCTCAAGATGTTCCCATTGTTTAAAGGAATTATGAGAGCAGTAGT
ATTTTTCTGTGATCAGTCTCTTACTATTACCAATGTGAGATTAAATTCTTATTATCCAGGATTTGT
GATTTTATGGGACAGACATTTACTATTGTTTTGAGCTTTGATTACCAGGGTACTTGTCTGAGTTG
TCATAAAGCCAATAGTAGAATGGCATGTGCTGCAGGCTGAGAACTGATAAAGGTGGAAGTCT
GTGCCTGGGAAGACTTTTGAGGACAACCTTGGACACAGCCTAGATAAGATCTTCCTGACTCT
ATTTCCCAATTGTGCTATTTCCTTTCTGACACGGGAAATCCCCACCTGATTAATCCCAGGCCC
TGCTTTTAACATCCAGTGACTATGCGATTACCTATCAGATATGACTCATGCCTGGAATCAAATG
GAGCTAAGGAAGTTCTTTCCTTCTGATATGATATATGACCCTGTCCCTCTGGAACATCCCTGT
GTTACCGTAATAGGATGCAAGTACATTCATGGCCACATAATAATAATATAGGGATAACATCCC
CCTAAGGAGGAAATGTTTATACACAATAATAGGACAAACGTGTAATGTGATAGATGTCTAATTA
AATAGGATAGCAAATTTTGAGAAAAGCAATAGGATTAGACTGATATGATTGGCTTCCAAGATGT
ATCTATTATAGCAATCCAAGCTTTAAGCATGTTTAGGTAGGAAGATCACAGTTTTGGATTTTTAC
ACTAAGCCAGAGAAGCAGAGCTCCCTTTTTGAACTGCCTATGATTAAACCGATTGTACATGA
GCAAGGAACTTAATATTAGTTAATATAGGTAATACTGATTGTATTGATTTGTATGATTCCTGTCTA
AGGTTTTCTTTGTTACCCTGATTACATGTAAATCTCTCTTTTCCAAACTAGTCTATTGTGAGCTCT
CTGATTGGTTCAATCTGTACCTGACTTAATGTAATTATGCAATTTTGCAAATTATGAGTAAACTTT
GTTGTATTGTTTGTTTGAACCTAGTCCTGTGTGACTGGGAAAACTGAACTATCTCTATGTGCCT
TTAACTAAAGACTTATGAGGGATTCTGGATACTTAGGAATTTAGTTTCCTTAATACTACTCTGGA
ATTTGTGTTATTTAAGAACTTTTGCACCAATTTAGTGAGGTTTTATGAACAAGAGTAGAAATGCA
TGCAAGCTTTTTAGGGCTCATTTTGGAAATGTTCCTTAGGCAATGTGACATCTTAGGGTACTAT
AGAACTGGGACTTGTGTTCTGTGTGTGTTCCTATATGAGTTAAAGTTCTTTACAATTAGGTCAG
GGGTTTGTGAGAATGTTATTTTGTTATTTGGGTAATATCATACTTGTTTAAAGTTGTGTTACAATTA
ATAATGTTAAAAGGGAAATGGCTTGTGGTTTATCTTCTCCATGTCATCAGAGAAACATGGCATG
ACTGAAACATTTAAGATCCTATATGCACTGTGTTAAAATGGATTCTTTCATATATCTATGTATGCA
TTGGGGCCTGTGGATTAATGTAAGGTATGCATATTGTTGTTTGCTCTTGTTTACTATTTAGTGGG
GGTTGGCAGTATGGGTCCGGCTCTTACGGCAGTCCTCTTCCTTGGATGATTCCTTTGCCAAA
AACTAAAAGGGGGAGTAGGAACTCAGGGCAGATGGCACTAATTCTTCCTTAACCAAGTTCTG
CAGGGACTCCAGAAAGAAAACACAGAGGCCACTACTGATCATACTTCTCTTGTGTTTGTTTG
GTCCATGTTTACTAAATATTCTTGCCAGATTCCTTTCTTCTAGGGTCGGAGCCATCAACTTCCA
GATGACTGTATACCAACCCAAGGTCTCTAGTCCACTGAACCTGGCATCAGCCAGGTTTTGAA
GTTTGACCCCTCGAACGGGACCCAGAGAGGCAGGGACAGCTCTACGCCCCTTGCCAGCA
GGAAGCAGTTTCAGAAGCAGAGACCTCCGCCCCTTTTCACCAAAAGAATTTGGTTCCTATTT
GTTTGAGGGGGGGATGATGGGGGCGGGGCCCAAACCAAGATCTTCGCTCTTTGCCTCAAA
GGGAATCTGACTCACAGGCCCCAGACCCTCATCTCCCTTTGAAAGCCTTAGCCTTACCTTTT

AATCTTATTCAGATCTGGCTCCCTTTTGTCTATAGGCCCCAGAAAGGCCTTCCTTTATCTCCTT
TTGAAAGTCCTAGCCCCACCTTGATTTTGTGCGGATGCCTTTCAAACTTCTGCCTTCCTGTTC
CCTCCATTATCTATTCCCACTGTCTATTCAATCTCCCTTGACCCTCCCTGCATTTCCCTATTCT
CCACTACCCCTCCCTGTTTCACATGCCCCCACCTACTTGCCCATCCCCCTTTTCCTTGGTTC
TCCTTCCCTTTTCCTCTGGCTCCTAGTAGGACCCCTCAGGCAGATCCTCCTCACCCCTTGAT
TATGAGACTGCTCCGAGCCATCCATAGCATTGTCAACTCCACCCGGCCTGACCTAGGACCT
AGCTGCTGGCTGTGTATGGATGCACAACCCCCATACTATGTGGGAGTGGCAGTTAATGGTTC
TGGTTCCCATAGCCCAGACTCGGAAAATTGTCGGTGGGAACAACCTAAGTTGACCCTGGGG
ATGTCCAAGGAAAAGGTACCTGTCTTACCTCATCCACCACATCCCTGAAAACTTCTCCTTACT
CTCCTGTCTGTAATAGTACTGTGGTTGTTCCCCAATCCTCCTCCTCTTTTTATTCAGGGCCATC
CGGCACGTGGTGGGCCTGTCTGGATGGGATCACACAGTGTGTCTCCGCTGCAGTGTTCCT
AAAAGAATCTGAGCCTCTATGTATATTAGTCTTCATTATCCCCAGGGTCTCCCTTCTTCAGGG
CCTGGAGGGTTGGGAGTACTTCAGTCATGAGGGCAGTGTGTCCTACTGAAGCAAGCGAGC
CGTTCCCCTCCTCATCCCTATCCTCAGTGGGGTTGGGGTTAGTGGGATCAACTGCTGTGGG
TACCGCTGCCCTAGTCCGAGGAGAGGCCAGTTACCAGGAACTTAGTGCTCAGGTCGATGC
AGACCTCAGTCAGTCATCTGGAGCACTCTACATCCCAACTGGAGAGACAGGTCAACTCCTT
GGCAGAGATGGTTCTGCAGAACCAGAGGGGTTTAGGCTTATTGTTTCTCAGGCAAGGTGGC
CTTTGTGCCGCCCTAGGGGAGGCCTGCTGTTTCTATGCCAATAACTCAGGTGTAGTTCAGGA
GAGCCTTTCCCTGGCACGGAAAAATATTGCAGACAGGCAGAGGGAACGAGAAGGGAACGA
AAATTGGTACCACAGCTGTTTTGGACTTCCCCATGGTTAACCATTCTTATTTCTGCCTTAGCCG
GGTCTTTGCTCCTCCTTACTATAGCCCTAATTCTTGGTCCTTGTCTAGTTAATCGGCTCCTTGA
CTTTGTTAGATCCTGTATTAATTTAGTTAAGTTACTGTTGGTTAAAGGTCCCCGTTATCAGACCC
TGGTGTCTGCCTTCTTTGCACCCTATAATGAGGCAGCCCCAACCACGTCAAGGGTTTGACG
TGGTACACAAAAGAAGTGGGGAATGTAATGCAGAACATTATTTTACCATTTGGGTAGTAGAAC
CCTTTAGGCCATGGAAAACTCCAAATTCCTTAGCAAGACCCTTATCTGGCTGCTCAGGCCAC
AGCCCTCAGATCATATCTCTGGAAGCCTCCATTGTGAGAAAGTTTGCAGAGCATTATCATATC
TACACAGAGGGAGAAGGGTGTCCTTGACCAGCCCCCCCCAAAAAACCTCTTCCTCTTGCC
AGACTTCAAAGATCCATCCCCCTCCCTGTCCTGTATACTCCCTATATAAACCCTAGCTCAAC
CCCTGATGGGGGCTGCTTGATTTGGATGTGAATATCCCTGGCAGCCGCGCGCTAATAGCTA
ATAAAGTCCACAATTTAAAATTAGCTTGGGTCGTCAACTCGCCTATAGGGATGAGAGTGGCC
CCAGGAGACTCAAGGAAAGGTTAGATAAGAGGCAGTTAGAGCACCAAAAGAATTTAAGTTGG
TACGAGGGATGGTTCAACCGTTCCCCCTGGCTTACTACTTTACTGTCTGCCCTTGCTGGTCC
CCTGCTACTCCTCCTTCTGTTACTCACCCTCGGGCCTTGTGTCATCAATAAGTTAGTGCAATT
CATCAATGATAGGGTTAGTGCAGTAAGGATTCTGGTTCTCAGGCACAAGTACCAGACCCTAG
ACAACGAGGATAACCTTTAATTCTGCTCTATGATTAGAGCTACCCTCAAAGAAAATGGGGGAA
TGAAGGAGGCAGAAATCATGAGGCAGAAATCATTCCGTGGAGTATGGAAACTACCCGGAGG
GCCCAAGGTTTAGGGACAGGTGCAGCCAGGCACAGTAAAAGGTCAGAGCAAGAAAAACAA
GGAAGATTTGGAGTGCCAAACGGGATATCTGTGGTCATGCACCTGAGTCCCCACCCCGGA
CTTATGCAAACAATTCCCAGAAATAGCTGAGCTCATAACAGTTTCTAGGGTGCCCCTCAGCA
GTTTCTAGAACCCTCTCGTGACCGGAGTTTTTATTCAAACTAACCAATGATCTTGCTCCTCGC
TTCTGTACCCGCGCTTTTTTGCTATAAAATGAGACCAAAGAATCCACCCGGCGCGCCAGTC
CCTCTAGGTGACTGAGTCGCCCGAGTACTCGTAAGTTCAATAAACCTCTTGCTATTTGCATC
CGGAGTTGTGTTCGCGTTGATCCTGGGAGGGTTTCTCAAGGTCGGAGGACTACCCGAACAT
TGGGGTCTTTCAA
>RecKoRV3-02-indels
CCACAATTTAAAATTAGCTCGGGTCGTCAACTCGCTCCGTGGAGTATGGAAACTACCCGGA
GGGCCCCAAGGTTTAGGGACAGGTGCAGCCAGGCACAGTAAAAGGTCAGAGCAAGAAAA

ACAAGGAAGATTTGGAGTGCCAAACGGGATATCTGTGGTCATGCACCTGAGTCCCCACCCC
GGACTTATGCAAACAATTCCCAGAAATAGCTGAGCTCATAACAGTTTCTAGGGTGCCCCTCA
GCAGTTTCTAGAACCCTCTCGTGACCGGAGTTTTTATTCAAACTAACCAATGATCTTGCTCCT
CGCTTCTGTACCCGCGCTTTTTTGCTATAAAATGAGACCAAAGAATCCACCCGGCGCGCCA
GTCCCTCTAGGTGACTGAGTCGCCCGAGTACTCGTAAGTTCAATAAACCTCTTGCTATTTGC
ATCCGGAGTTGTGTTCGCGTTGATCCTGGGAGGGTTTCTCAAGGTCGGAGGACTACCCGAA
CATTGGGGTCTTTCATTTGGGGGCTCGTCCGGGATCTGAGATTCCCACCCAAGGACCGCC
GAACCACCGACGGGAGGTAAGCTGGCCAGCGATCGCTCTATGTTCTCTCTGTGTCTGACTC
CGAAAACTCTGACTGTCTATTCGGTGTGCGCGCATTTTTGGTTTCAGTTTATTCCGGGTTAATC
GATCTAAGATCGAGGCGTGAGTAGCGGACAGACGTGTTCGGGGGCTCACCGCCCGGCAA
TCCTGGGAGACGTCCCAGGATCAGGGGAGGACCAGGGACGCCTGGTGGATCCCACGGC
AAGGGATAATTCTCTTCTGATCTCACAGTTGCACTGTCTTGAGAGACATACCCTACCTTCTGA
CTCTTTTTCCTGTCTTTTTAATATACGTCTACGCCGCCATATAATTTTTTTCCTTTTTATTTCAATAA
TAATCATTATCTGGGTCGTAATTCCACTCTTCGGGACCCCCAGAATGGGACAGGGTGAGTC
GACCCCTCTCTCTCTTACACTAGATCACTGGAAAGACGTGAAGACAAGGGCTCACAATCTTT
CCGTGGAGATAAGAAAGGGAAAGTGGCAAACCTTCTGTTCCTCCGAGTGGCCCACGTTCG
AAGTGGGATGGCCACCGGAGGGGACTTTTAATCCTTCTATTATTTCTGCAGTCAAAAGGATTG
TCTTCCAGGAGACTGTTGGACCTGGTGAAAGGGTCTAGAAAGAAGAAAGATTTTTTTATTGGG
TCAATTGTATTAGTTATGTTGCTGGAAGGGCAATTTCTTTTATAATCTGGAATGTTAAATTAATTGT
GTCTTCAAAAAAAAAAAAAAAAGGGAGTCAATTTGGCTGTTGGGGCATTTTATATGATCCTGTTTT
GTAACATTTCCTTTTAAATATTGTAATTCAAGGAAGTATAAGATTTTCAAATTTTGTCTCTATTGAA
AGGGAATTTTCAGTTGAAATTGTTTTGTTATAATTTATAAAATTTGTGAGATTGTTAACTAAGTTTTG
GGGTTATTGTTTTAATATCAAAACCTAAGATTGTTAATTGATTACTTGTGTGTGAAAAGGAGGGG
CACATGGCAATTCCCCTTATTTTGTAAACTTGCCAGGATCCCTGAAAACTTCTCCTTACTCTC
CTGTCTGTAATAGTACTGTGGTTGTTCCCCAATCCTCCTCCTCTTTTTATTCAGGGCCATCCG
GCACTTGGTGGGCCTGTCTGGATGGGATCACACAGTGTGTCTCCGCTGCAGTGTTCCTAAA
AGAATCTGAGCCTCTATGTATATTAGTCTCCATTATCCCCAGGGTCTCCCTTCTTCAGGGCCC
GGAGGGTTGGGAGTACTTCAGTCATGAGGGCAGTGTGTCCTACTGAAGCAAGCGAGCTGTT
CCCCTCCTCATCCCTATCCTCAGTGGGGTTGGGGTTAGTGGGATCACCTGCTGTGGGTACC
GCTGCCCTAGTCCGAGGAGAGGCCAGTTACCAGGAACTTAGTGCTCAGGTCGACGTAGAC
CTCAGTCATCTGGAGCACTCTATATCCCAACTGGAGAGACAGGTCAACTCCTTGGCGGAGA
TGGTTCTGCAGAACCGGAGGGGTTTGGACTTATTGTTTCTCAGGCAAGGCGGCCTTTGTGCT
GCCCTAGGGGAGGCCTGCTGTTTCTATGCCAATAATTCAGGTGTGGTTCAGGAGAGCCTTTC
CCTAGTACGGAAAAATATTGCAGACAGGCAGAGGGAACGGGAAGGGAACGAAAATTGGTA
CCACAGCCTGTTTTGGACTTCCCCATGGTTAACCATTCTTATTTCTGCCTTAGCCGGGTCTTT
GCTCCTCCTTACTATAGCCCTAATTCTTGGTCCTTGTCTAGTTAATCGGCTCCTTGACTTTGTT
AGATCCTGTATTAATTTAGTTAAGTTACTGTTGGTTAAAGGTCCCCGTTATCAGACCCTGGTGT
CTGCCTTCTTTGCACCCTATAATGAGGCAGCCCCAACCACGTCAAGGGTTTGACGTGGTAC
ACAAAAGAAGTGGGGAATGTAATGCAGAACATTATTTTACCATTTGGGTAGTAGAACCCTTTA
GGCCATGGAAAACTCCAAATTCCTTAGCAAGACCCTTATCTGGCTGCTCAGGCCACAGCCC
TCAGATCATATCTCTGGAAGCCTCCATTGTGAGAAAGTTTGCAGAGCATTATCATATCTACAC
AGAGGGAGAAGGGTGTCCTTGACCAGCCCCCCCCAAAAAACCTCTTCCTCTTGCCAGACT
TCAAAGATCCATCCCCCTCCCTGTCCTGTATACTCCCTATATAAACCCTAGCTCAACCCCTG
ATGGGGGCTGCTTGATTTGGATGTGAATATCCCTGGCAGCCGCGCGCTAATAGCTAATAAAG
TCCACAATTTAAAATTAGCTCGGGTCGTCAACTCGCTCCGTGGAGTATGGAAACTACCCGGA
GGGCCCAAGGTTTAGGGACAGGTGCAGCCAGGCACAGTAAAAGGTCAGAGCAAGAAAAA
CAAGGAAGATTTGGAGTGCCAAACGGGATATCTGTGGTCATGCACCTGAGTCCCCACCCC

GGACTTATGCAAACAATTCCCAGAAATAGCTGAGCTCATAACAGTTTCTAGGGTGCCCCTCA
GCAGTTTCTAGAACCCTCTCGTGACCGGAGTTTTTATTCAAACTAACCAATGATCTTGCTCCT
CGCTTCTGTACCCGCGCTTTTTTGCTATAAAATGAGACCAAAGAATCCACCCGGCGCGCCA
GTCCCTCTAGGTGACTGAGTCGCCCGAGTACTCGTAAGTTCAATAAACCTCTTGCTATTTGC
ATCCGGAGTTGTGTTCGCGTTGATCCTGGGAGGGTTTCTCAAGGTCGGTGGACTACCCGAA
CACTGGGGTCTTTCAC
>Koala-01-SA1_01
AGTGAAGGAGGCAGAAATCATGAGGCAGAAATCATTCCGTGAAGTATGGAAACTACCCGAG
GGCCCAAGGTTTAGGGACAGGTGCAGCCAGGCACAGTAAAAGGTCAGAGCAAGAAAAACA
AGGAAGATTTGGATTGCCAAACAGAATATCTGTGGTCATGCACCTGAGTCCCCACCCCGGA
CTTATGCAAACAATTCCCAGAAATAGCTGAGCTCATAACAGTTTCTAGGGTGCCCTCAGCAG
TTTCTAGAACCCTCTCGTGACCGGAGTTTTTATTCAAACTCAACCAATGATCTTGCTCCTCGC
TTCTGTACCTCGCGCTTTTGCCATAAATGAGACCATAAGAACCACCCGGCGCGCCAGTCCT
CTCAGGTGACTGAGTCGCCCGAGTACTCGTAAGTTCAATAAACCTCTTGCTATTTGCATCCG
GAGTTGTGTTCGCGTTGATCCTGGGAGGGTTTCTCGAGGTCGGTGGACTACCCGAACATCG
GGGTCTTTTCATTTGGGGCTCGTCCGGGATCTGAGATTCCCACCCGCGACCGCCGAACCA
CCGACGGGAGGTAAGCTGGCCAGCGATCGCTCTATGTTCTCTCTCTGTGTCTGACTCCGAA
AACTCTGACTGTCTATTCGGTGTGCGCGCATTTTTGGTTTCCAGTTTATTCCGGGTTAACCGAT
CTAAGATCGAGGCGTGAGTAGCGGACAGACGTGTCTCGGGGCTCACCGCCCGGCAATCC
TGGGAGACGTCCCAGGATCAGGGAGGACCAGGGACGCCTGGTGGACCCCACGGCAAGG
GATAATTCTCTTCTGATCTCACAGTTGCACTGACTTGAGAGACATACCCTACCTTCTGACTCTT
TTCCTGTCTTTAATATACGTCTACGCTGCCATATAATTTTTTTTTTTTCCTTTTATTTCACATAATAA
TCATTATCTGGGTCGTAATTCCACTCTTCGGGACCCCAGAATGGGACAGGGTGAGTCGACC
CCTCTCTCTCATCACTAGATCACTGGAAAGACGTAGATGACAAGGGCTCACAATCTTTCCGT
GGAGATAAGAAAGGGAAAGTGGCAAACCTTCTGTTCCTCCGAGTGGCCCACGGACATCGG
CCTCGGGAGGGGGAATTGTTCTGTTCCTGACTTCTAGGGAGCCGGCCCCTGATGATGTTCC
AGGGCCCCAGGACTCAGGACCTAGGAGAAGCCTCCTCCCCGACTCCGACTGTACCTCATT
TCGGCCATCGTGGACCTGGTAACCCTCTGGAGGTAAATGTTTGCAAACCTGTTTTCTGGTGG
GGGAACGAGTGCCAGACGTGGCAGAGCACCTAATTAGTGCCTCAACCCCCAGGCACCCC
GGGAGACGTCGGGACTATGCCCTGTATCTGGTTCCTGGTACCCGGTTTCTGGTTCCTGGTTT
CTGTCTTAGTTCTGTTGGGCCCGTGTCTCGTTCCTGGTTTCTGTCCCAGTGTTGGTCTATTAGT
TTATATGTGTAGAGATTGTGTTTTTAAGAGATTCTTGTTTGCAATCATTCTGCCTGAAGCCACCT
CTGTTTCATACTGAAAGACAGGTGTCAGAGTAGATTGCAGACAGTTTTCTCCAGGGCAGTTC
CTTGACTTCCATTGGGAGGATGTGTTAGTGTTCAGGCTCTTATTGTTTGTCTGTGTCTGTGTTCT
GCAATAATGGGGGATACGACCAGTACATCATTTCCTCCTTTGGGTTGTTTGCATAGCCAGAC
AGCCAAAGGCTGCAGCAGGACTCCTGAAAAGTTGCCTTCATTCTCCCCTCTTCCTCCCCAG
GTAACAGCGGCTTCAAAGCAGTGCGTGGATGGGAGAGGGAAAGAGAGAAGAATACTTTATT
ATTTAGGTTATATGGTTGGTAGAGGGCAAGTTGGGAAGGATTTTTGAAGTGTCAGGTCAGAATT
AGAAAGGGATGAGATATGTACAGGTTTTAAGGGGATTTTAAATCATTAGAAAGAAAGGCAAGG
AAGTTTAAGCATGTAGGAGAGAGGGAGTGTGTGTAGAAAATTTTGAAGGAATATGATTTAGTTG
CATGTGTGTGACCTGGAGGTTTTGGAAAGTGAGGTTTTGGGACTTTTGCATGAGTAATAGTTTC
TCTCTCTCTCTGCATTCTAACTCCTGGCCAAGTTTGGAGTGTGGAGACAAAGAGAAAAGTTCT
TGTGTACCAGAACATAGCAAATGGTTTATGTGCTGAGGACTGCGGCTTGCTGGGTTTAGTTGA
TATTTTCCCACGAGCCCCGGCTCATCCAAATTGGTAAATATGTGAGGTTTTCAACTTCAACATT
TGTTACACTGTTTTGCACTTAGGTACATTGAAGGAAACCTCAGACAAGTGGATTGTTTTGAGTG
CACAGTAAGGGCAGTTTGAGGAGAGATGGAGAGAATTATGTCCACCCACCTTTCAGGCTAG
ATCCCCGGGTGGGTTCTTTTAAAGACTGGTCATTATGGGAAAGAAAAAGCAATCAGAATACT

GGTCCAGTACATTTCTAAGCTTAACAAGGGTATTGCATTTGGGAATATATTCTTGTCTACTCCT
CCAAAAACTAAATAAATAAATAGGGGAGAATGAATGGGTTAGAATAAACTTCCAAATTTTATGT
GCTTTTAAGAGGAAGTTTCAACCTATAATTTTATCAAATCTGTGTAAACGTTTTCGTGTTGTGAAA
TTCTGAGTAAATGAGAATTCTTGAGAGATAAAATTGAGTAAACTTTTAAAAGTTTGATAATATTTG
TTATTTGGGTAATATCATACTTGTTTAAAGTTGTGTTACAATTAATAATGTTAAAAGGGAAATGGC
TTTGTGGTTTATCTTCTCCATGTCATCAGAGAAACATGGCATGACTGAAACATTTAAGATCCTA
TATGCACTGTGTTAAAATGGATTCTTTCATATATCTATGTATGTATTGGGGCCTGTGGATTAATG
TAAGGTATGCATATTGTTGTTTGCTCTTGTTTACTATTTAGTGGGGGTTGGCAGTATGGGTCCG
GCTCTTATGGCAGTCCTCTTCCTTGGATGATCCCTTTGCCAAAAACTAAAAGGGGGAGTAGG
AACTCAGGCAGATGGCACTAATTCTTCCCTAACCAAGTTCCTGCAGGGACTCCAGAAAGAA
AACACAGAGGCCACTACTGATCATACTTCTCTTGTGTTTGTTTGGTCCATATGTTTACTAAATAT
TCTTGCCAGATTCCTTTCTTCTAGGGTCGGAGCCATCAACTTCCAGATGACTGTATACCAACC
CAAGGTCTCTAGTCCACTGAACCTGGCATCAGCCAGGTTTTGAAGTTTGACCCCTCGAATG
GGGACCCAGAGAGGCAGGGACAGCTCTACGCCCCTTGCCAGCAGGAAGCAGTTTCAGAA
GCAGAGACCTCTCGCCCCTTTTCACCAAAAGAATTTGGTTCCTATTTGTTTGAGGGGGATGAT
GGGGCGGGGCCCAAACCAAGATCTTCGCTCTTTGCCTCAAAGGGAATCTGACTCACAGGC
CCCAGACCCTCATCTCCCTTTGAAAGCCTTAGCCTTACCTTTTAATCTTATTCAGATCTGGTTC
TTTGTCTATAGGCCCCAGAAAGGCCTTCCTTTATCTCCTTTGAAAGTCCTAGCCCCACCTTTG
ATTTTGTGCAGATGCCTTCAAACTTCTGCCTTCCTGTTCCCTCCTCCATTATCTATTCCCACTG
TCTATTCAATCCCCTTGACCCCCTGCATTTCCCTATTTTCCACTACCCCTCCCTGTTTCACAT
GCCCCCACCTACTTGCCCATCCCCCTTTCCTTGGTTCTCCTTCCTTTTCCTCTGGCTCCTAG
TAGGACCCTCAGGCAGATCCTCCTCACCCCTCGATTATGAGACTGCTCCGAGCCATCCATA
GCGTTGTCAACTCCACCTCGGCCTGACCTAGGACCTAGCTGCTGGCTGTGTATGGATGCAC
AACCCCACACTATGTGGGAGTGGCATGTTAATGGTTCTGGTTCCCATAGCCCAGACTCGGA
AAATTGTCGGTGGAACAACCTAAGTTGACCGTGGGGGATGTCCAAGGAAAAGGTACCTGTC
TTACCTCATCCACCACATCCCTGAAAACTTCTCCTTACTCTCCTGTCTGTAATAGTACTGTGGT
TGTTCCCCAATCCTCCTCCTCTTTATTCAGGCCATCCGGCACTTGGTGGGCCTGTCTGGATG
GGGATCACACAGTGTGTCTCCGCTGCAGTGTTCCTAAAAGAATCTGGAGCCTCTATGTATATT
AGTCTCCATTATCCCCAGGGTCTCCCCTTCTTCAGGGCCCGGAGGGTTGGGGAGTACTTCA
GTCATGAGAGGCAGTGTGTCCTACTGAAGCAAGCTGAGCTGTTCCTCTCCTCATCCCTATCC
TCAGTGGGGTTGGGGTTAGTGGGATCACCTGCTGTGGGTACCGCTGCCCTAGTCCGAGGG
AGAGGCCAGTTACCAGGAACTTAGTGCTCAGGTCGACGTAGACCTCAGTCATCTGGAGCA
CTCTATATCCCAACTGGAGAGACAGGTCAACTCCTTACGGAGATGGTTCTGCAGAACCGGA
GGGGTTTGGACTTATTGTTTCTCAGGCAAGGCGGCCTTTGTGCTGCCCTAGGGAGGCCTGC
TGTTTCTATGCCAATAATTCAGGTGTGGTTCAGGAGAGCCTTCCCTAGTACGGAAAAAAATAT
TGCAGACAGGCAGAGGGAACGGAAGGGAACGAAAATTGGTACCAGAGCCTGTTTCGGACT
TCCCCATGGTTAACTCACTCTTATTTCTGCCTTAGCCGGGCCTTTCCTCTTCACCATAGCCCT
AATTCTTGGTCCTTGTCTAGTTAATCAGCTCCTTTGACTTTGTTAGATCCCGTATTAATTCAGGT
AAGTTACTGTTGGTTAGAGATCCCCGCTATCAGCCCCCCTGGCGTCTCGCCTCCTTTACAC
CCTATGATGAGATAGCCCCCACCACGTCAAGGGTTTGACGTGGTACACAAAAGAAGTGGG
GAATGTAATGCAGAACATTATTTTACCATTTAGGTAGTAGGGCCCTTTAGGCAATGGAAAGCA
GAAGTTGCTGCTTAATCTTGCCCCAGACCTCTTTTCCTCCTAAATCTAAACAATAGTGAAATGT
ACTCCTGCATGGCTTAAGCACAGCCCATACCTCCAAAGCAAATGGATAAGAAAAACTTGCAA
TACAATGGCTTTAGATAATTATGTTTATACCAGAAACCAGGACACCTGGTCTGGTTTGTGTGAT
CCCTGACCTGTAATGATGGGGTGCTCGGGTGCTTGTGCTTATACTTTTGCCCCCAAAATGCC
TCCCTAGTCTCAGAAACACTATCTCTGCAACCTGACTATACCCTTAAACGCCTCACATACTC
ACAAGTACCTCCTCCTCCAACCACAGCTGGTGGGCTTGTAATTCTGGCCTCACCCCTGCCT

CTCTACATCAGTCTTCAACCAGTTCTAACGATTTCTGTATCCAGATCCAGCTTGTCCCTCGCA
TCTACTATCACCCAGACGGTACCTTGCTACAGGCCTATGAGTCCCCCACCCTAGAAACAAA
GAGAGAGCCTGTCTCACTCACCCTGGCTGTCCTTTCTCGGATTAGGGTCGCAGCAGGTATA
GGTACCGGCTCGACCGCTCCTAATAAAAGGGCCCATAGACCTCCAAACAAGGTTTGACTAG
CCTCCAGATTGCCATGGATACAGACCTTAGGGCCCTTCAAGACTCCATAGGCAAACTAGAG
GATTCCTTAACCCTCCCTGTCTGAAGTAGTGCTCCAGAATAGGGAGAGGCCTTGATCTGCTA
TTTTTGAAGGAAGGGGGCCTTTGAGCAGCCCTAAAAGAGGAATGCTGTTTCTATGTTTGACCA
CTCCAGGCGCGGTGCGAGGAACTCCATGAGGGAGACTCAAGGAAAGGTTGAGATAAGAG
GCAGTTAGAGCACCAAAAAGAATTTAGGTTGGTTCTGAGGATGGTTCAACCGTTCCCCCTGG
CTTACTACTTTACTGTCTGCCCTTGCTGGTCCCCTGCTACTCCTCCTTCTGTTACTCACCCTC
GGGCCTTGTGTCATCAATAAGTTAGTGCAATTCATCAATGATAGGGTTAGTGCAGTAAGGATT
CTGGTTCTCAGGCACAAGTACCAGACCTAGACAACGAGGATAACCTTTAATTCTGCTCTATG
ATTAGAGCTACCCTCAAAGAAAATGGGGAATGAAGGAGGCAGAAATCATGAGGCAGAAATC
ATTCCGTGAAGTATGGAAACTACCCGGAGGGCCCAAGGTTTAGGGACAGGTGCAGCCAGG
CACAGTAAAAGGTCAGAGCAAGAAAAACCAAGGAAGATTTGGATTGCCAAACAGAATATCTG
TGGTCATGCACCTGAGTCCCCACCCGGACTTATGCAAACAATTCCCAGAAATAGCTGAGCT
CATAACAGTTTCTAGGGTGCCCCTCAGCAGTTTCTAGAACCCTCTCGTGACCGGAGTTTTTAT
TCAAACTAACCAATGATCTTGCTCCTCGCTTCTGTACCCGCGCTTTTGCTATAAAATGAGACC
AAAGAATCCACCCGGCGCGCCAGTCCCTCTAGGTGACTGAGTCGCCCGAGTACTCGTAAG
TTCAATAAACCTCTTGCTATTTGCATCCGGGTTGTGTTCGCGTTGATCCTGGGAGGGTTTCTC
AAGGTCGGTGGACTACCCGAACATCGGGGTCTTTCAT
>Koala-01-SA1_02
AAATCATGAGGCAGAAATCATGAGGCAGAAATCATTCCGTGAAGTATGGAAACTACCCGGA
GGGCCCAAGGTTTAGGGACAGGTGCAGCCAGGCACAGTAAAAGGTCAGAGCAAGAAAAC
AAGGAAGATTTGGAGTGCCAAACAGAATATCTGTGGTCATGCACCTGAGTCCCCACCCCGG
ACTTATGCAAACAATTCCCAGAAATAGCTGAGCTCATAACAGTTTCTAGGGTGCCCTCAGCA
GTTTCTAGAACCCTCTCGTGACCGGAGTTTTTATTCAAACTAACCAATGATCTTGCTCCTCGC
TTCTGTACCCGCGCTTTTTGCTATAAAATGAGACCAAAGAATCCACCCGGCGCGCCAGTCC
CTCTAGGTGACTGGAGTCGCCCGAGTACTCGTAAGTTCAATAAACCTCTTGCTATTTGCATC
CGGAGTTGTGTTCGCGTTGATCCTGGGAGGGTTTCTCAAGGTCGGTGGACTACCCGAACAT
CGGGGTCTTTCATTTGGGGGCTCGTCCGGGATCTGAGATTCCCACCCAAGCGACCGCCGA
ACCACCGACGGGAGGTAAGCTGGCCAGCGATCGCTCTATGTTCTCTCTGTGTCTGACTCCG
AAAACTCTGACTGTCTATTCGGTGTGCGCGCATTTTTGGTTTCAGTTTATTCCGGGTTAATCGA
TCTAAGATCGAGGCGTGAGTAGCGGACAGACGTGTTCGGGGCTCACCGCCCGGCAATCCT
GGGAGACGTCCCAGGATCAGGGAGGACCAGGGACGCCTGGTGGACCCCACGGCAAGG
GATAATTCTCTTCTGATCTCACAGTTGCACTGTCTTGAGAGACATACCCTACCTTCTGACTCTT
TTTCCTGTCTTTTAATATACGTCTACGCCGCCATATAATTTTTTTTTTTTTTCCTTTTTATTTCAATAA
TAATCATTATCTGGATCGTAATTCCACTCTTCGGGACCCCCAGAATGGGACAGGGTGAGTCG
ACCCTCTCTCTTACACTAGATCACTGGAAAGACGTGAAGACAAGGGCTCACAATCTTTCCGT
GGAGATAAGAAAGGGAAAGTGGCAAACCTTCTGTTCCTCCGAGTGGCCCACGTTCGAAGTG
GGATGGCCACCGGAGGACTTTAATCCTTCTATTATTTCTGCAGTCAAAAGGATTGTCTTCCAG
GAGACTGGAGGACACCCGGACCAGGTTCCCTACATCATAGTTTGGCAGGACCTCTCCAAC
AGCCCCCCATGGGTGCCACCCTTAGCCAAGATCGCCGCTGCCTCTGGTCAAGATAACGG
GCGAAAGTCGGCGGGGGAGGCCGTCCGCTCCTTCCCGGCTCCCCATCTACCCGGAGAC
GGACAGCCTGTTCCTCCTCTCAGAACCCCCGCCCTATCCAACATCCCCTCCCTGTCCTGT
ATACCCCTATATAAACCCTAGCTCAACCCCTGATGGGGGCTGCTTGATTTGGATGTGAATAT
CCCTGGCAGCCGCGCGGCTAATAGCTAATAAAGTCCACAATTTAAAATTAGCTCGGGTCGT

CAACTCGCTCATTTCTCGGTATAACATTTTGGAGGCCCCAGCGAGATAGCAGCGTACCACG
TCGCTGCAGAGACTCCACCTGGAGAGGACATCGGCCTCGGGAGGGGGAATTGTTCTGTTC
TGACTTCTAGGGAGCCGGCCCCTGATGATGTTCCAGGGCCCCAGGACTCAGGACTAGGAG
AAGCCTCCTCCCCGACTCCGACTGTACCTCATTTCGGCCCTCGTGGACCTGGTAACCCTCT
GGAGGTAAATGTTTGCAAACCTGTTTTCTGGTGGGGAACGAGTGCCAGACGTGGCAGAGCA
CCTAATTAGTGCCTCAATCTCCCCAGGCACCCCGAGACGTTGGGACTATGCCTGTATCTGG
TTCCTGGTACCCGGTTTCTGGTTCCTGGTTTCTGTCTTAGTTCTGTTGGGCCCGTGTCTTCGTT
CCTGGTTTCTGTCTCAGTGTTGGTCTATTAGTTTATATGTGTAGAGATTGTGTTTTTAAGAGATTC
TGTTTTGCAATCATCTGCCTGAAGCCACCTCTGTTTCATACTGAAAGACAGGTGTCAGAGTAG
ATTGCAGACAGTTTTCTCCAGGGCAGTTCCTTTGACTTCCATTGGGAGGGGATGTGTTAGTGT
TCAGGCTCTTATTGTTTGTCTGTGTCTATGTTCTGCAATAATGGGGGATACGACCAGTACATCA
TTTTCCCTCCTTTTGGGTTGTTTGCAGCCAGACAGCCAAAGGCTGCAGCAGGACTCTGAAAA
GTTGCCTTCATTCTCCCCTCTTCCTCCCCAGGTAACAGCGGCTTCAAAGCAGTGCGTGGAT
GGGAGAGGGAAAGAGAGAAGAATACTTTATTATTTAGGTTATATGGTTGGTAGAGGGCAAGTT
GGGGAAGGATTTTTTGAAGTGTCAGGTCAGAATTAGAAAGGAATGAGATATGTACAGGTTTTA
AGGGGATTTTAAATCATTAGAAAGAAAGGCAAGGAAGTTTAAGCATGTAGGAGAGAGAGTGT
GTGTAGAAAATTTGAAGGGAATATGATTTAGTTGCATGGTGTGACTGGAGGTTTTGAAAGTGAG
GTTTTGGGACTTTTGCATGAGTAATAGTTTCTCTCTCTCTGCATTCTAACTCTGGCCAAGTTTGA
GTGTGGAGACAAAGAGAAAACTTCTTGCGTACCAGAACATAGCAAATGGTTTATGTGCTGAG
GACTGCGGCTTGCTGGGTTTAGTTGATATTTTCCCACGAGCCCCAGCTCATCCAAATTGGTA
AATATGTGAGGTTTTCAATTTCAACATTTGTTACACTGTTTTGCACTTAGGTACATTGGAAGGAA
ACTCAGACAAGTGGATTGTTTTGAGTGCACAGTAAGGGCAGTTTGAGGAGAGATGGAGAGAA
TTATGTCCACCCACCTTTCAGGCTAGATCCCGGGTGGGTTCTTTTAAAGACTGGTCATTATGG
GAAAGAAAAAGCAATCAGAATACTGGTCCAGTACATTTCTAAGATTAACAAGGGTATTGCATTT
GGGAATATATTCTTGTCTACTCCTCCAAAAACTAAATAAATAAATAGGGGAGAATGAATGGGTT
AGAATAAACTTCCAAATTTTATGTGCTTTTAAGAGGAAGTTTCACCTATAATTCTTATCAAATCTG
TGTAAACGTTTTCTGTGTTGTGAAATTCCTGAGTAAATGAGAATTCTTGAGAGATAAAATTGAGT
AAACTTTTAAAGTTTGATAATATTTGTTATTTGGGTAATATCATATTTGTTTAAAGTTGTGTTACAAT
TAATAATGTTAAAAGGAAATGGCTTGTGGTTTATCTTCTCCATGTCATCAGAGAAACATGGCAT
GACTGAAACATTTAAGATCCTATATGCACTGTGTTAAAATGGATTCTTTCATATATCTATGTATGT
ATTGGGGCCTGTGGATTAATGTAAGGTATGCATATTGTTGTTTGCTCTTGTTTACTATTTAGTGG
GGGTTGGCAGTATGGGTCCGGCTCTTATGGCAGTCCTCTTCCTTGGATGATTCCTTGCCAAA
AACTAAAAAGGGGGAGTAGGAACTCAGGGCAGATGGCACTAATTCTTCCTTAACCAAGTTCC
TGCAGGGACTCCAGAAAGAAAACACAGAGGCCAACTACTGATCATACTTCTCTTGTGTTTGTT
TGGTCCATGTTTACTAAATATTCTTGCCAGATTCCTTTCTTCTAGGGTCGGAGCCATCAACTTC
AGATGACTGAATACCAACCCAAGGTCTCTAGTCCACTGAACCTGGCATCAGCCAGGTTTTGA
AGTTTGACCCCTCGAATGGGACCCAGAGAGGCAGGGACAGCTCTACGCCCCTTGCCAGC
AGGAAGCAGTTTCAGAAGCAGAGACCTCCGCCTTTCTCACCAAAAGAATTTGGTTCCTATTT
GTTTGAGGGGGATGATGGGGGCGGGGCCCAAACCAAGATCTTCGCTCTTGCCTCAAAGGG
AATCTGACTCACAGGCCCCAGACCCTCATCTCCCTTTTGAAAGCCTTAGCCTTACCTTTTAAT
CTTATTCAGATCTGGCTCCCTTTGTCTATAGGCCCCAGAAAGGCCTTCCTTTATCTCCTTTTGA
AAGTCCTAGCCCCACCTTGATTTTGTGCAGATGCCTTTCAAACTTCTGCCTTCCTGTTCCCTC
CATTATCTATTCCCACTGTCTATTCAATCTCCCTTGACCCCCTGCATTTCCCTATTTTCCACTA
CCCCTCCCTGTTTCACATGCCCCCACCTACTCTGCCCATCCCCCTTTTCCTTGGTTCTCCTT
CCCTTTTCCTCTGGCTCCTGGTAGGACCCCTCAGGTCAGATCCTCCTCACCCTCGATTATG
AGACTGCTCCGAGCCATCCATAGCGTTGTCAACTCCACCCGGCCTGACCTAGGACCTAGC
TGCTGGCTGTGTATGGATGCACAACCCCACACTAGTGTGGGAGTGGCAGTTAATGGTTCTG

GTCTCCCATAGCCCAGACTCGGAAAATTGTCGGTGGGAACAACCTAAGTTGACCGTGGGG
ATGTCCAAGGAAAAGGTACCTGTCTTACCTCATCCACCACATCCCTGAAACTTCTCCCTACT
CTCCTGTCTGTAATAGTACTGTGGTTGTTCCCCAATCCTCCTCCTCTTTTATTCAGGGCCATC
CAGGCACTTGGTGGGCCTGTCTGGATGGGATCACACAGTGTGTCTCCGCTGCAGTGTTCCT
AAAAGAATCTGAGCCTCTATGTATATTAGTCTCCATTATCCCCAGGGTCTCCCTTCTTCAGGG
CCCGGAGGGTTGGGAGTACTTCAGTCATGAGGGCAGTGTGTCCCTACTGAAGCAAGCGAG
CTGTTCCCCTCCTCATCCCTATCCTCAGTGGGGTTGGGGTTAGTGGGATCACCTGCTGTGG
GTACCGCTGCCCTAGTCCGAGGAGAGGCCAGTTACCAGGAACTTAGTGCTCAGGTCGACG
TAGACCTCAGTCATCTGGAGCACTCTATATCCCAACTGGAGAGACAGGTCAACTCCTTGGC
GGAGATGGTTCTGCAGAACCGGAGGGGTTTGGACTTATTGTTTTTCTCAGGCAAGGCGGCC
TTTGTGCTGCCCTAGGGGAGGCCTGCTGTTTCTATGCCAATAACTCAGGTGTAGTTCTGGAG
AGCTCCTTTCCCTGGCACGGAAAAATATTGCAGACAGGCAGAGGGAACGAGAAGGGAACG
AAAATTGGTACCAGAGCCTGTTTCGACTTCCCCATGGTTAACCACTCTTATTTCTGCCTTAGC
CGGGCCTTTCCTCTTCACCATAGCCCTAATTCTTGGTCCTTGTCCAGTTAATCAGCTCCTTGA
CTTTGTTAGATCCCGTATTAATTCAGGTAAGTTACTGTTGGTTAGAGATCCCTCGCTTATCAGC
CTCCCTGGCGTCTGCCTCCTTTACACCCTATGATGAGATAGCCCCCACCACGTCAAGGGTT
TGACGTGGTACACAAAAGAAGTGGGGAATGTAATGCAGAACATTATTTTACCATTTAGGTAGT
AGGGCCCTTTAGGCAATGGAAAGCAGAAGTTGCTGCTTAATCTTGCCCCAGACCTCTTTCCT
CCTAAATCTAAACAATAGTGAAATGTACTCCTGCATGGCTTAAGCACAGCCCATACCTCCAA
AGCAAATGGATAAGAAAACTTGCAATACAATGGCTTTTAGATAATTATGTTTATACTACCAGAAA
CCAGGACACCTGGTCTGGTTTGTGTGATCCCTGACCTGTAATGATGGGGTGCTCGGGTGCT
TGTGCTTATACTTTTGCCCCAAAATGCCTCCCTAGTCTCAGAAACACTATCTCTGCAACCTGA
CTATACCCTTAACGCCTCACATACTCACAAGTACCTCCTCCCCTCCAACCACAGCTGGTGG
AGCTTGTAATTCTGGCCTCACCCCTGCTCTCTACATCAGTCTTCAACCAGTCTAACGATTTCT
GTATCCAGATCCAGCTTTGTCCCTCGCATCTACTATCACCCAGACGGTACCTTGCTACAGG
CCTATGAGTCCCCCCACCCTCAGAAACAAGAGAGAGCCTGTCTCACTCACCCTGGCTGTC
CTTCTCGGATTAGGGGTCGCAGCAGGTAGTAGGTACCGGCTCGACCGCCCTAATAAAAGG
GCCCATAGACCTCCAACAAGGTTTGACTAGCCTCCAGATTGCCAATGGATACAGACCTTAG
GGCCCTTCAAGACTCCATAAGTAAACTAGAGGATTCCTTAACCTCCCTGTCTGAAGTAGTGC
TCCCAGAATAGGAGAGGCCTTGATCTGCTTATTTTTTGAAGGAAGGGGGCGCTTTGTTGCAG
CTCCCTAAAAGAGGAATGCTGTTTCTATGTTGACCACTCAGGCGCGGTGCGAGACTCCATG
AGAGACTCAAGGAAAGGTTAGATAAGAGGCAGTTAGAGCACCAAAAAATTTAAGTTGAGTAC
GAGGGATGGTTCAACCGTTCCCCTGGCTTACTACTTTACTGTCTGCCCTTGCTGGTCCCCTG
CTACTCCTCCTTCTGTTACTCACCCTCGGGCCTTGTGTCATCAATAAGTTAGTGCAATTCATC
AATGATAGGGTTAGTGCAGTAAGGATTCTGGTTCTCAGGCACAAGTACCAGACCCTAGACAA
CGAGGATAACCTTTAATTCTGCTCTATGATTAGAGCTACCCTCAAAGAAAATGGGGAATGAAG
GAGGCAGAAATCATGAGGCAGAAATCATGAGGCAGAAATCATGAGGCAGAAATCATTCCGT
GAAGTATGGAAACTACCGGAGGGCCCAAGGTTTAGGGACAGGTGCAGCCAGGCACAGTAA
AGGTCAGAGCAAGAAAAACAAGGAAGATTTGGAGTGCCAAACAGAATATCTGTGGTCATGCA
CCTGAGTCCCCACCCCGGACTTATGCAAACAATTCCCAGAAATAGCTGAGCTCATAACAGT
TTCTAGGGTGCCCCTCAGCAGTTTCTAGAACCCTCTCGTGACCGGGTTTTTATTCAAACTAAC
CAATGATCTTGCTCCTCGCTTCTGTACCCGCGCTTTTTGCTATAAAATGAGACCAAAGAATCC
ACCCGGCGCGCCAGTCCCTCTAGGTGACTGAGTCGCCCGAGTACTCGTAAGTTCAATAAA
CCTCTTGCTATTTGCATCCGGAGTTGTGTTCGCGTTGATCCTGGGAGGGTTTCTCAAGGTCG
GTGGACTACCCGAACATCAGGGGTCTTTCAC
>Koala-01-SA1_03

GGTGAAGGAGGCAGAAATCATGAGGGCAGAAATCATTCCGTGAAGTATGGAAACTACCCGG
AGGGCCCAAGGTTTAGGGACAGGTGCAGCCAGGCACAGTAAAAGGTCAGAGCAAGAAAA
CAAGGAAGATTTGGAGTGCCAAACAGAATATCTGTGGTCATGCACCTGAGTCCCCACCCCT
GGACTTATGCAAACAATTCCCAGAAATAGCTGAGCTCATAACAGTTTCTAGGGTGCCCTCTA
GCAGTTTCTAGAACCCTCTCGTGACCGGAGTTTTTATTCAAACTAACCAATGATCTTGCTCCT
CGCTTCTGTACCCGCGCTTTTATGCTATAAAATGAGACCAAAGAACCACTCTGGCGCGCCA
GTCCTCTAGGTGACTGAGTCGCCCGAGTACTCGTAAGTTCAATAAACCTCTTGCTATTTGCAT
CCGGAGTTGTGTTCGCGTTGATCCTGGGAGGGTTTCTCAAGGTCGGTGGACTACCCGAACA
TCGGGGTCTTTCATTTGGGGGCTCGTCCGGGATCTGAGATTCCCACCCAGCGACCGCCGA
ACCACCGACGGGAGGTAAGCTGGCCAGCGATCGCTCTATGTTCTCTCTGTGTCTGACTCCG
GAAAACTCTGACTGTCTATCTCGGTGTGCGCGCATTTTTGCTCTCAGCTTGTGTCCCGCGCT
AACCGACTAAGATCGAGGCGTGAGTAGCGGACAGACGTGTTCGGGGCTTCACCGCCCGG
CAATCCTGGGGACGTCCCAGGATCAGGGAGGACCAGGGACGCCTCGGTGGACCCCACG
GCAAGGGATAATTCTCTTCTGATCTCACAGTTGCACTGACTTGAGAGACATACCCTACCCTC
TGACTCTTTTTCCTGTCTTTTAATATACGTCTACGCCGCCATATAATTTTTTTTTTTTTATTTCAAT
AATAATCATTATCTGGGTCAATTCCACTCTTCGGGACCCCAGAATGGGACAGGGTGAGTCTG
ACCCTCTCTCTCTTACGACTAGATCACTGAAAGACGTGAAGACAAGGGCTCACAATCTTTCC
GTGGAGATAAGAAAGGGAAAGTGGCAAATCCTTCTGTTCCTCCGGAGTGGCCCACGTTCGA
AGTGGGATGGCCACCGGAGGGGACTTTAATCCTTCTATTATTTCTGCAGTCAAAAGGATTGTC
TTCCAGGAGACTGGAGTGGAGGCCCTCTAGCGAGATAGCAGCGTACCGTGTCGCTGCAGA
GACCCACCTGGAGAGGACATCGGCCTCGAGGTGGGAATTGTTCTGTTCTGACTTCTAGGGA
GCCGGCCCCGATGATGTTCCAGGGCCCCAGGACTCAGGACTAGGAGAAGCCTCCTCCCC
GACTCCGACTGTACCTCATTTCGGCCATCGTGGGACCTGGTAACCCTCTGGAGGTAAATGT
TTGCAAACCTGTTTTCTGGTGGGGAACGAGTGCCAGACGTGGCAGAGCACCTAATTAGTGC
CTCAATCCCCCAGGCACCCTGAGACGTCGGGACTATGCCTGTATCTGGTTCCTGGTACCC
GGTTTCTGGTTCCTGGTTTCTGTCTTAGTTCTGTTGGGCCCGTGTCTCGTTCCTGGTTTCTGTC
TCAGTGTTGGTCTATTAGTTTATATGTGTAGAGATTGTGTTTTTAAGAGATTCTGTTTTGCAATCAT
CTGCCTGAAGCCACCTCTGTTTCATACTGAAAGACAGGTGTCAGAGTAGATTGCAGACAGTT
TTCTCCAGGGCAGTTCCTTTGACTTCCATTGGGAGGGATGTGTTAGTGTTCAGGCTCTTATTG
TTTGTCTGTGTCTGTGTTCTGCAATAATGGGGGATACGACCAGTACATCATTTCCTCCCTTTGG
GTTGTTTACAGCCAGACAGCCAAAGGCTGCAAGCAGGACTCTGAAAGTTGCTTCGATTCTC
CCCTCTTCCTCCCCAGGTAACAGCGGCTTCAAAAGCAGTGCGTGGATGGGAGAGGGAAGA
GAGAAGAATACTTTATTATTTAGGTTATATGGTTGGTAGAGGGCAAGTTGGGAAGGATCTTTGA
AGTGTCAGGTCAGGAATTAGAAAGGGATGAGATATGTACAGGTTTTAAGGGATTTTAAATCATT
AGAAAGAAAGGCAAGGAAGTTTAAGCATGTAGGAGAGAGGAGTGTGTGTAGAAAATTTGAAG
GGAATATGATTTAGCTGCATGGTGTGACTGAGGTTTTGAAAGTGAGGTTTTGGGACTTTTGCAA
ATGAGTAATAGTTTCTCTCTCTCTGCATTCTAACTCTGGCCAAGTTTGAGTGTGGAGACAAAG
AGAAAGTTCTTGTGTACCAGAACATAGCAAATGGTTTATGTGCTGAGGACCGCGGCTTCTGC
TGGGTTTAGTTGATATTTTCCCACGAGCCCCAGCTCATCCAAATTGGTAAATATGTGAGGTTTT
CAATTTCAACATTTGTTACACTGTTTTGCACTTAGGTACATTGAAGGAAACTCAGACAAGTGGA
TTGTTTTGAGTGCACAGTAAGGGCAGTTTGAGGAGAGATGGAGAGAATTATGTCCACCCACC
TTTCAGGCTAGATCCCCGGGTGGGTTCTTTTAAAGACTGGTCATTATGGGAAAGAAAAAGCA
ATCAGAATACTGGTCCAGTACATTTCTAAGCTTAACAAGGGTATTGCATTTGGGAATATATTCTT
GTCTACTCCTCCAAAAACTAAATAAATAAATAGGGGAGAATGAATGGGTTAGGAATAAACTTC
TCAAATTTTATATGTGCTTTTAAGAGGAAGTTTCACCTATAATTTTATCAAATCTGTGTAAACGTTT
TCTGTGTTGTGAAATTCCTGAGTAAATGAGAATTCTTGAGAGATAAAAAATTGAGTAAACTTTTAA
AAGTTTGATAATATTTGTTATTTGGGTAATATCATACTTGTTTAAAGTTGTGTTGCAATTAATAATGT

TAAAAGGAAATGGCTTGTGGTTTATCTTCTCCATGTCATCAGAGAAACATGGCATGACTGAAA
CATTTAAGATCCTATATGCACTGTGTTAAAATGGATTCTTTCATATATCTATGTATGTATTGGGGC
CTGTGGATTAAATGTAAGGTATGCATATTGTTGTTTGCTCTTGTTTACTATTTAGTGGGGGTTGG
CAGTATGGGTCCGGCTCTTATGGCAGTCCTCTTCCTTGGATGATTCCTTGCCAAAAACTAAAA
GGGGAGTAGGAACTCAGGGCAGATGGCACTAATTCTTCCTTAACCAAGTTCCTGCAGGACT
CCATGAAAGAAAACACAGAGGCCACTACTGATCATACTTCTCTTGTGTTTGTTTGGTCCATGTT
TACTAAATATTCTTGCCAGATTCCTTTCTTCTAGGTCTGGAGCCATCAACTCCAGATGACTGTA
TACCAACCCAAGGTCTCTAGTCCACTGAACCTGAGCATCAGCCAGGTTTTGAAGTTTGACCC
TCGAATGGGACCCAGAGAGGCAGGGACAGCTCTACGCCCCTTGCCAGCAGGAAGCAGTT
TCAGAAGCAGAGACCTCCGCCCCTTTTCACCAAAAGAATTTGGTTCCTATTTGTTTGAGAGG
GGATGATGGGGCGGGCCCAAACCAAGATCTTCGCTCTTTGCCTCAAAGGGAATCTGACTCA
CAGGCCCCAGACCCTCATCTCCTTTGAAAGCCTTAGCCTTACCTTTTTAATCTTATTCAGATCT
GGCCCCTTTCGTCTATAGGCCCCAGAAAGGCCTTCCTTTATCTCCTTTGAAAGTCCTAGCC
CCACCTTGATTTTGTGCAGATGCTCTTTCAAACTTCTGCCTTCCTGTTCCCTCCATTATCTATT
CCCACTGTCTATTCAATCTCCCTTGACCCCCTGCATTTCCCCTATTTTCCACTACCCCTCCCT
GTTTCACATGCCCCCACCTACTTGCCCATCCCCCCTTTTCCTTGGTTCTCCTTCCTTTTCCTC
TGGCTCCTAGTAGGACCCCTCAGGCAGATCCTCCTCACCCCTCGATTATGAGACTGCTCC
GGCCATCTCATAGCGCTGTCTAACTCCACCCGGCCTGACCTAGGACCTAGCTGCTGGCTG
TGTATGGATGCACAACCCCACACTATGTGGAGTGGCAGTTAATGGTTCTGGTTCCCACAGC
CCAGACTCGGAAGAATTGTCGGTGGGAACAACCTAAGCTGACCGTGGGGATGTCCAATGG
AAAAGGTACTCTGTCTTACCTCATCCACCACATCCCTGAAAGCTTCTCCTTACTCTCCTGTCT
GTAATAGTACTGTGGTTGTTCCCCAATCCTCCTCCTCTTTTATTCAGGGCCATCCGGCACTTA
GCGGGCCTGTCTGGATGGGATCACACAGTGTAGTCTCCGCTGCAGTGTTCCTAAAAGAATC
TGAGCCTCTATGTATATTAGTCTCCATTATCCCCAGGGTCTCCCTTCTTCAGGGCCCGGAGG
GCTGGGAGTACTTCAGTCATGAGGGCAGTGTGTCCTACTGAAGCAAGCGAAGCTGTTCCCT
CCTCATCCCTTATCCTCAGTGGGGTTGGGGTTAGTGGGATCACCTGCTGTGGGTACCGCTG
CCCTAGTCCGAGGAGAGGCCAGTTACCAGGAACTTAGTGCTCAGGTCGACGTAGACCTCT
AGTCATCTGGAGCACTCTATATCCCAACTGGAGAGACAGGTCAACTCCTTGGCGGAGATGG
TTCTGCAGAACCTGAGGGGTTTGGACTTATTGTTTCTCAGGCAAGGCGGCCTTTGTGCTGCC
CTAGGGAGGCCTAGCTGTTTCTATGCCAATAATTCAGGTGTGGTTCAGGAGAGCCTTTCCTA
GTACGGAAAAATATTAGCAGACAGGCAGAGGGAACGGGAAGGAACGAAAATTGGTACCAG
AGCCTGTTTCGGACTTCCCCATGGTTAACCACTCTTATTTCTGCCTTAGCCGGGCTCTTTCCT
CTTCACCATAGCCCCAATCTTGGTCCTTGTCTAGTTAATCAGCTCCTTGACTTTGTTAGATCC
CAGCATTAATTCAGGTGTTACTGTTGGTTAGAGATCCCCGCTATCAGCTCCCTGGCGCCTGC
CTCCTTTACACCCTATGATGAGACAGTCCCCCACCACGTCAAGTGGTTTGACGCGGTACAC
AAAAGAAGTGGGAATGTAATGCAGAACATTATTTTACCATTTAGGTAGTAGGGCCCTTTAGGC
AATGGAAAGCAGAAGCTGCTGCTTAATCTTGCCCCAGACCTCTTTCCTCCTAAATCTAAACAA
TAGTGAAATGTACTCCTGCATGGCTTAAGCTAACAGCCCATACCTCCAAAGCAAATGGATAA
GAAAACTTGCAATACAATGGCTTTAGATAATTATGTTTATGCCCAGAAACCAGGACACCTGGT
CTGGTTTGTGTGATCCCTGACCTGTAATGATGGGGTGCTCGGGTGCTTGTGCTTATACTTTTG
TCCCCAAAATGCCTCCCTAGTCTCAGAAACACTATCTCTGCAACCTGACTATACCCTTTAAA
CGCCCCACATACTCACAAGTACCTCCTCCCTCCAACCACAGCTGGTGGAGCTTGTAATTCT
GGCCTCACCCCTGCCTCTCTACATCAGTCTTCAACCAGTCTAACGATTTCTGTATCCAGATC
CAGCTTGTCCTCGCATCTACTATCACCCAGACGGTACCTTGCTACAGGCCTACGAGTCCCC
CACCCTAGAAACAAGAGAGAGCCTGTCTCACTCACCCTGGCTGTCCTTTCTCGGATTAGGG
GTCGCAGCAGGTATAGGTACCGGCTCGACCGCTCCTAATAAAGGGCCCCATAGACCTCCA
ACAAGGTTTGACTAGCCTCCAGATTGCCATGGATACAGACCTTAGGGCCCTTCAAGACTCC

ATAAGTAAACTAGAGATTCCTTAACCTCCCTGTCTGAAGTAGTGCTCCAGAATAGGAGAGGC
CTTGATCTGCTATTTTTGAAGGAAGGGGCATTTGTACAGCCCTAAAAGAGAATGCTGTTTCTAT
GTTGACCACTCAGGCGCGTTGCGAGACTCCATGAGGAGACTCAAGGAAAGGTTAGATAAGA
AACAGTTCAACGCAAAAGAATTTAAGTTGAATGCGAAGGGATGGTTCAACCGTTCCCCCCTG
AGCACTACTTTACTGTCTGCCCTTGCTGGTCCCCTGCTACTCCTCCTTCTGTTACTCACCCT
CGGGCCTTGTGTCATCAATAAGTTAGTGCAATTCATCAATGATAGGGTTAGTGCAGTAAGGAT
TCTGGTTCTCAGGCACAAGTACCAGACCCTAGACAACGAGGATAACCTTTAATTCTGCTCTA
TGATTAGAGCTACCCTCCAAGAAAATGGGGAATGAAGGAGGCAGAAATCATGAGGCAGAAA
TCATTCCGTGAAGTATGGAAACTACCCGGAGGGCCCAAGGTTTAGGGACAGGTGCAGCCA
GGCACAGTAAAAGGTCAGAGCAAGAAAACAAGGGAAGATTTGGAGTGCAAACAGAATATCT
GTGGTCATGCACCTGAGTCCCCACCCGGACTTATGCAAACAATTCCCGAAATAGCTGAGCT
CATAACAGTTTCTAGGGTGCCCCTCAGCAGTTTCTAGAACCCTCTCGTGACCGGAGTTTTTAT
TCAAACTAACCAATGATCTTGCTCCTCGCTTCTGTACCCGCGCTTTTTGCTATAAAATGAGAC
CAAGAATCCACCCGGCGCGCCAGTCCCTCTAGGTGACTGAGTCGCCCGAGTACTCGTAA
GTTCAATAAACCTCTTGCTATTTGCATCCGGAGTTGTGTTCGCGTTGATCCTGGGAGGGTTTC
TCAAGGTCGGTGGACTACCCGAACATCGGGGTCTTTCAC
>Koala-01-SA1_04
ACTGAAGGAGGCAGAAATCATGAGGCAGAAATCATTCCGTGAAGTATGGAAACTACCCGGA
GGGCCCAAGGTTTAGGACAGGTGCAGCCAGGCACAGTAAAAGGTCAGAGCAAGAAAAACA
AGGAAGATTTGGAGTGCCAAACAGAATATCTGTGGTCATGCACCTGAGTCCCCACCCCGGA
CTTATGCAAACAATTCCCAGAAGTAGCTGAGCTCATAACAGTTTCTAGGGTGCCCCTCAGCA
GTTTCTAGAACCCTCTCGTGACCGGAGTTTTTATTCAAACTAACCAATGATCTTGCTCCTCGC
TTCTGTACCCGCGCTTTTTGCTATAAAATGAGACCAAAGAATCCACCCGGCGCGCCAGTCC
CTCTAGGTGACTGAGTCGCCCGGAGTACTCGTAAGTTCAATAAACCTCTTGCTATTTGCATC
CGGAGTTGTGTTCGCGTTGATCCTGGGAGGGTTTCTCAAGGTCGGTGGACTACCCGAACAT
CGGGGTCTTTCATTTGGGGGCTCGTCCGGGATCTGAGATTCCCACCCAAGGACCGCCGAA
CCACCGACGGGAGGTAAGCTGGCCAGCGATCGCTCTATGTTCTCTCTGTGTCTGACTCTGA
AAACTCTGACTGTCTATTCGGTGTGCGCGCATTTTTGGTTTCAGTTTATTCCGGGTTAATCGAT
CTAAGATCGAGGCGTGAGTAGCGGACAGACGTGTTCGGGGCTCACCGCCCGGCAATCCT
GGGAGACGTCCCAGGATCAGGGGAGGACCAGGGACGCCTGGTGGACCCCACGGCAAG
GGATAATCCTCTTCTGATCTCACAGTTGCACTGTCTTGAGAGACATACTCTACCTTCTGACTC
TTTTCCTGTCTTTTTAATACACGTCTACGCTGCCATATAATTTTTTTTTCCTTTTATTTCAATAATAA
TCATTATCTGGGTCGTAATTCCACTCTTCGGGACCCCCAGAATGGGACAGGGTGAGTCGAC
CCTCTCTCTCTTACACTAGATCACTGGAAAGACGTGAAGACAAGGGCTCACAATCTTTCCGT
GGAGATAAGAAAGGGAAAGTGGCAAACCTTCTGTTCCTCCCGAGTGGCCCACGTTCGAAGT
GGGGATGGCCACCGGAGGGGACTTTAATCCTTCTATTATTTCTGCAGTCAAAAGGATTGTCTT
CCAGGAGACTGGAGGACACCCTGACCAGGTTCCCTACATCATAGTTTGGCAGGACCTCTC
CAACAGCCCCCCATGGGTGCCACCCTTAGCCAAGATCGCTGCTGCCTCTGGTCAAGATAA
CGGGCGAAAGTCGGCGGGGGAGGCCGTCCGCTCCTTCGGCTCCCCCATCTACCCGGAG
ACGGACAGCCTGTTCCTCCTCTCAGAACCCCCGCCTATCCAACATCCCCTCCCTGTCCTG
TATACTCCCTATATAAACCTAGCTCAACCCCTGATGGAGGCTGCTTGATTTGGATGTGAATAT
CCCTGGCAGCCGCGACTAATAGCTAATAAAGTCCACAATTTAAAATTAGCTCAGGTCGTCAA
CTCGCTCATTTCTCGGTATGAGGCCCCAGCAGAGATAGCAGCGTACCGTGTCGCTGCAGA
GACCCACCTGGAGAGGACATCGGCCTCAGGAGAGGTGTTCTGTTCTGACTTCTGGGGAGC
CGGCCCTGATGATGTTCCAGGGCCCCAGGACTCAGGACTGGGAGAAGCCTCCCTCCCGA
CTCCGACTGCCATACCTCATTTCGGCCATCGTGGACCTGGTAACCCTCTGGAGGTAAATGT
CTGCAAACCTGTTTTCTGGTGGGGAACGGAGTGCCGGACGTGGCAGAGCACCTAATTAGTG

CCTCAATCCCCAGGCACCCCGAGACGTCGGGACTATGCCTGTATCTGGTTCCTGGTACCC
GGTTTCTGGTTCCGGTTTCTGTCTTAGTTCTGTTGGGCCCGTGTCTCGTTCCTGGTTTCTGTCT
CAGTGTTGGTCTATTAGTTTATATGTCGTAGAGATTGTGTTTTTAAGAGATTCTGTTTTGCAATCA
TCTGCCTGAAGCCACCTCTGTTTCATACTGAAAGACAGGTGTCAGAGTAGATTGCAGACAGT
TTTCTCAGAGCAGTTCCTTTGAAGCTTCCATTGGGAGGATGTGTTAGTGTTCAGAGCTCTTATT
GTTTGTCTGTGTCTGTGTTCTGCAATAGTGGGGATACGACCAGTACATCATTTCCCTCCCTTTT
GGGTTGTTTGCAGCCAGACAGCCAAAGGCTGCAGCAGGACTCTGAAAGAGTTGCCTTCATT
CTCCCCTTTTTCCTCCCCAGGTAACAGCGGCTTCAAAGCAGTGCGTGGATGGGAGAGGAAA
GAGAGAAATACTTTATTATTTAGGTTATATGGTTGGTAGAGGGCAAGTTGGGAAGGATTTTTGA
AGTGTCAGGTCAGAATTAGAAAGGGATGAGATATGTACAGGTTTTAAGGGATTTTTAAATCATT
AGAAAGAAAAGGCAAGGAAGTTTAAGCATGTAGGAGAGAGAGTGTGTGTAGAAAATTTGAAG
GAATATGATTTAGTTGCATGGTGTGACTGGAGGCTTTGAAAGTGAGGTTTGGGACTTTGCATG
AGGTAATAGTTTCTCTCTCTCTCTGCATTCTAACTCTAGCCAAGATTTGAGTGTGGAGACAAGA
GAAAAGTTCTTGTGTACCAGAACATAGCAAATGGTTTATGATGCTGAGGACTGCGGCTTGCT
GGGTTTAGTTGATATTTTCCCACAGCCCCAGCTCATCCAAATTAGTAAATATGTGAGGTTTTCA
ATTTCAACATTTGTTACACTGTTTTGCACTTAGGTACATTGAAGGAAACTCAGACAAGTGGATT
GTTTCTTGAGTGCACAGTAAGGGCAGTTTGAGGGAGAGATGGAAGAATTATGTCCACCCACC
TTTCAGGCTAGATCCCGGGTGGGTTCTTTTAAAGACTGGTCATTATGGGAAAGAAAAAAGCAA
TCAAGAATACTGGTCCAGTACATTTCTAAGCTTAACAAGGGTATTGCATTTGGGAATATATTCTT
GTCTACTCACTCCAAAAACTAAATAAATAAATAGGGGAGAATGAATGGGTTAGAATAAACTTC
CAAATTTTATGTGCTTTTAAGAGGAAGTTTCACCTATAATTCTTATCAAATCTGTGTAAGCGTTTT
CTGTGTTGTGAAATTCCTGAGTAAATGAGAATTCTTGAGAGATAAAAATTGAGTAAACTTTTAAA
AGTTTGATAATATTTGTTATTTGGGTAATATCATACTTGTTTAAAGTTGTGTTACAATTAATAATGTT
AAAGGAAATGGCTTGTGGTTTATCTTCTCCATTGTCATCAGAGAAACATGGCATGACTGAAAC
ATTTAAGATCCTATATGCACTGTGTTAAAATGGATTCTTTCATATATCTATGTATGTATTGGGGCC
TGTGGATTAATGTAAGGTATGCATATTGTTGATTGCTCTTGTTTACTATTTAGTGGGGGTTGGCA
GTATGGGTCCAGCTCTTATGGCAGTCCTCTTCCTGGATGATTCCTTTGCCAAAAACTAAAGG
GGGTGTAGGAACTCAGGGCAGATGGCACTAATTGCTTCCTTAACCAAGTTCCTGCAGGGAC
TCCAGAAAAGAAAACACAGAGGCCACTACTGATCATCACTTCTCTTGTGTTTGTTTGGTCCAT
GTTTACTAAATATTCTTGCCGAATTCCTTTCTTCTAGAGTCGGAGCCAATCAACTTCCAGATGA
CTGTATACCAACCCAGAGTCTCTAGTCCACTGAACCTGGCATCAGCCAGGTTTTGAAGTTTG
ACCCCTCGAATGGGACCTAGAGAGGCAGGGACAGCTCTACGCCCTTGCCAGCAGGAAGC
AGTTTCAGAAGCAGAGACCTCCGCCCTTTTCACCAAAGAATTTGGTTCCTATTTGTTTTGAGG
GGATGATGGGGCAGGGCCCAAACCAAGATCTTCGCTCTTTGCCTCAAGGGAATCTGACTCA
CAGACCCCAGACCCTCATCTCCCTTTGAAAGCCTTAGCCTTACCTTTTAATCTTATTCAGATC
TGGCTCCCTTTTGTCTATAGGCCCCAGAAAGGCCTTCCTTTATCTCCTTGAAAGTCCTAGCC
CCACCTTGATTTTGTGCAGATGCCTTTCAAACTCTCTGCCTTCCTGTTCCCTCCATTATCTATT
CCCACTGTCTATTCAATCTCCACTTGACCCCCTGCATTTCCCTATTTCCACTACCCCTCCCT
GTTCACATACCCCCACCTACTTGCCCATCCCCCTTTTCCTTGGTTTCTCCTTCCCTTTTCCTC
TGACTCCTAGTAGGACCCCTCAGAGTCAGATCCTCCTCACCCCTCGATTATGAGACTGCTC
CGAGCCATCCATAGCGTTGTCAACTCCTACCCGGCCTGACCTAGGACCTAGCTGCTGGCT
GTGTATGGATGCACAACCCCACACTATGTGGGAGTGGCAGTTAATGGTTCTGGTTCCCATAG
CCCAGACTCGGAAAATTGTCGGTGGGAACAACCTAAGTTGACCGTGGGGGGATGTCCAAG
GAAAAAGGTACCTGTCTTACCTCATCCACCACATCCCTGAAAACTTCTCCCTTACTCTCCTGT
CTGTAATAGTACTGTGGTTTGTTCCCCAATCCTCCTCCTCTTTTTATTCAGGGCCATCCGGCA
CTTGGTGGGCCTGTCTGGATGGGATCACACAGTGTGTCTCCGCTGCAGTGTTCCTAAAAGA
ATCTGAGCCTCTATGTATATTAGTCTCCATTATCCCCAGGGTCTCCCTTCTTCAGGGCCCGG

AGGGGTTGGGAGTACTTCAGTCATGAGGGCAGTGTGTCCTACTGAAGCAAGCTGAGCTGTT
CCCCTCCTCATCCCTATCCTCAGTGGGGTTGGGGTTAGTGGGATCACCTGCTGTAGGGTAC
CGCTGCCCTAGTCGAGAGAGGCCAGTTACCAGGAACTTAGTGCTCAGGTCGACGTAGACC
TCCAGTCATCTGGAGCACTCTATATCCCAACTGGAGAGACAGGTCAACTCCTTAGCGGAGA
TGGTTCTGCAGAACCGGAGGGGTTTGGACTTATTGTTTCTCAGAGCAAGGCGGCCTTTGTGC
TGCCCTAGGGAGGCCTGCTGTTTCTATAGCTCAGTAATTCAGGTGTGGTTCAGGAGAGCCTT
TCCCTAGTACGGAAAAATATTGCAGACAGGCAGAGGAGCGAGGAAGGGAACGAAAATTGGT
ACTCAGAGCCTGTTTCGGACTTCCCCATGGTTAACCACTCTTATTTCTGCCTTAGCCGGACC
TTTCCTCTTCACCATAGCCCTAATTCTTGGTCCTTGTCTAGTTAATCAGCTCCTTGACTTTGTTA
GATCCCGTATTAATTCAGGTAAGTTACTGTTGGTTTAGAGATCCCGCTATCAGCCCCTGGCGT
CTGCCTCCTTACACCCTATGATGAGACTAGCCCCCACCACGTCAAGGGTTTGACGTGGTAC
ACAAAGAAGTGGGAATGTAATGCAGAACATTATTTTACCATTTGGGTAGTAGGACCCTTGTAG
GCAATGGAAAGCAGAAGTTGCTGCTTAATCTTGCCCCAGACCTCTTTCCTCCTAAATCTAAAC
AATAGTGAAATGTACTCCTGCATGGCTTAAGCACAGCCCATACCTCCAAAGCAAATGGATAA
GAAAACTTTGCAATACAATGGCTTTTAGATAATTATGTTTTATATACCAGAAACCAGGACACCT
GGTCTGGTTTGTGTGATCCCCTGACCTGTAATGATGGGGTGCTCGAGGTGCTTGTGCTTATA
CTTTTGCCCCAAAATGCCTCCCTAGTCTCAGAAACACTATCTCTGCAACCTGACTATACCCTT
AAACGCCTCACATACTCACAAGTACCTCCTCCCTCCAACCACAGCTGGTGGGCTTGTAATT
CTGGCCTCACCCCCTGCCTCTCTACATCAGTCTTCTAACCAGTCTAACGATTTCTGTATCCA
GATCCAGCTTGTCCCTCGCATCTACTATCACCCAGACGGTACCTTGCTACAGGCCTATGAG
TCCCCCACCCTAGAAACAAGAGAGCCTGTCTCACTCACCCTGGCTGTCCTTCTCGGATTAG
GGGTCGCAGCAGGTATAGGTACCGACTCGACCGCCTAATAAAGGGCCCATAGACCTCAA
CAAGGTTTGACTAGCCTCCAGATTGCCATGGATACAGACCTTAGGGCCCTTCAAGACTCCAT
AAGTAAACTAGAGGATTCCTTAACCTCCCTGTCTGAAGTAGTGCTCCAGAATAGGAGAGGCC
TTGATCTGCTATTTTTGAAGGAAGGGGGCCTTTGTGCAGCCCTAAAGAGGAATGCTGTTTCTA
TGTTGACCACTCAGGCGCGGTGCGAGGCACTCCATGAGGAGACTCAAGGAAGGGTTAGAT
AAGAGGCAGTTAGAGCACCAAAAAATTTAAGTTGGAGTACGAGGGATGGTTCAACCCGTTCC
CCCTGACTTACTACTTTACTGTCTGCCCTTGCTGGTCCCCTGCTACTCCTCCTTCTGTTACTC
ACCCTCGGGCCTTGTGTCATCAATAAGTTAGTGCAATTCATCAATGATAGGGTTAGTGCAGTA
AGGATTCTGGTTCTCAGGACAACGAGGATAACCTTTAATTCTGCTCTATGATTAGAGCTACCC
TCAGAAAATGGGGAATGAAGGAGGCAGAAATCATGAGGCAGAAATCATTCCGTGAAGTATG
GAAACTACCGGAGGGCCCAAGGTTTAGGGACAGGTGCAGCCAGGCACAGTAAAGGTCAG
AGCAAGAAAAACAAGGAAGATTTGGGTGCCAAACAGAATATCTGTGGTCATGCACCTGAGTC
CCCACCCCGGACTTATGCAAACAATTCCCAGAAGTAGCTGAGCTCATAACAGTTTCTAGGGT
GCCCCTCAGCAGTTTCTAGAACCCTCTCGTGACCGGGTTTTTATTCAAACTAACCAATGATCT
TGCTCCTCGCTTCTGTACCCGCGCTTTTTGCTATAAAATGAGACCAAAGAATCCACCCGGCG
CGCCAGTCCCTCTAGGTGACTGAGTCGCCCGAGTACTCGTAAGTTCAATAAACCTCTTGCTA
TTTGCATCCGGAGTTGTGTTCGCGTTGATCCTGGGAGGGTTTCTCAAGGTCGGTGGACTACC
CGAACATCAGGGTCTTTCAT
>Koala-01-SA1_05
GTTGAAGGAGGCAGAAATCATGAGGCAGAAATCATTCCGTGAAGTATGGAAACTACCCGGA
GGGCCCAAGGTTTAGGACAGGTGCAGCCAGGCACAGTAAAAGGTCAGAGCAAGAAAACAA
GGAAGATTTGGAGTGCCAAACAGAATATCTGTGGTCATGCACCTGAGTCCCCACCCCGGAC
TTATGCAAACAATTCTCTGGTGAAATAGCTGAGCTCATAACAGTTTCTAGGTGCCCTCAGCAG
TTTCTAGAACCCTCTCGCGACCGGAGTTTTTATTCAAACTAACCAATGATCTTGCTCCTCGCT
TCTGTACCCGCGCTTTGCTATAAAATGAGACCAAAGATCCACCCGGCGCGCCAGTCCTCTA
GGTGACTGGAGTCGCCCGAGTACTCGTAAGTTCAATAAACCTCTTGCTATTTGCATCCGGAG

TTGTGTTCGCGTTGATCCTGGGAGGGTTTCTCAGGGTCGGTGGACTACCCGAACATCGGGG
TCTTTCATTTGGGGCTCGTCCGGGATCTGAGATTCTCGACCCAAGGACCGCTGAACCACCG
ACGGGAGAGGTAAGCTGGCCAGCGATCGCTCTATGTTCTCTCTGTGTCTGACTCCGAAAAC
TCTGACTGTCTATTCGGTGTGCGCGCATTTTTGGTTTCAGTTTATTCCGGTTAATCGATCTAAG
ATCGAGGCGTGAGTAGCGGACAGACGTGTTCGGGGGCTCACCGCCCGGCAATCCTGGGA
GACGTCCCAGGATCAGGGGAGGACCAGGGACGCCTGGTGGACCCTACGGCAAGGTAATT
CTCCTTCTGATCTCACAGTTGCACTGACTTGAGAGACATACCCTACCCTCTGACTCTTTTCCT
GTCTTTTAATATACGTCTACGCCGCCATATAATTTTTTTTTTTTTTATTTCAATAATAATCATTATCT
GGGTCGTAATTCCACTCTTCGGGACCCCCAGAATGGGACAGGGTGAGTCGACCCCCTCTC
TCTTACACTAGATCACTGGAAAGACGTGAAGACAAGGGCTCACAATCTTTCCGTGGAGATAA
GAGGGGAAAGTGGCAAACTTTTCTGTTCCTCCGGAGTGGCCCACGTTCGAAGTGGGATGG
CCACCGGAGGGGACTTTTTAATCCTTCTATTATTTCTGCAGTCAAAAGGATTGTCTTCCAGGA
GACTGGAGTGGAGGCCCTAGCGAGATAGCAGCGTACCGTGTCGCTGCAGAGACCCACCT
GGAGAGTGACATCGGCCTCGGGAGGGGAATTGTTCTGTTCTGACTTCTAGGGGAGCCGGC
CCCTGATGATGTTCCAGGGCCCCAGGACTCAGGACTAGGAGAAGCCTCCCTCCCGACTC
CGACTGTACCTCATTTCGGCCATCGTGGACCTGGTAACCCTCTGGAGGTAAATGTTTGCAAA
CCTGTTTTCTGGTGGGGAACGAGTGCCATGACGTGGCAGAGCACCTAATTAGTGCCTCAAT
CCCCAGGCACCCCGAGACGTCGGGACTATGCCTGTATCTTGGTTCCTGGTACCCGGTTTCT
GGTTCCTGGTTTCTGTCTTAGTTCTGCTGGGCCCGTGTCTCGTTCCTGGTTTCCTGTCTCAGT
GTTGGTCTATTAGTTTATATGTGTAGAGATTGTGTTTTTAAGAGATTCTGTTTTGCAATCATCTGC
CTGAAGCCACCTCTGTTTCATACTGAAAGACAGGTGTCAGAGTAGATTGCAGACAGTTTTCTC
CAGGGCAGTTCCTTTGACTTCCATTGGGAGGATGTGTTAGTGTTCAGGCTCTTATTGTTTGTCT
GTGTCTGTGTTCTGCAATAATGGGGGATACGACCAGTACATCATTTCCCTTTTGGGTTGTTTG
CAGCCAGACAGCCAAAGGCTGCAGCAGGACTCTGAAAAGTTGCCTTCATTCTCCCCTCTTC
CTCCCCAGGTAACAGCGGCTTCAAAGCAGTGCGTGGATGGGAGAGGGAAAGAGAGAAGA
ATACTTTATTATTTAGGTTATATGGTTGGTAGAGGGCAAGTTGGGAAGGATTTTTGAAGTGTCAG
GTCAGAATTAGAAAGGGATGAGATATGTAACAGGTTTTAAGGGGATTTTAAATCATTAGAAAGA
AAGGCAAGGAAGTTTAAGCATGTAGGAGAGAGGAGTGTGTGTAGAAAATTTGAAGGGAATAT
GATTTAGTTGCATGAGTGTGACTGGAGGTTTTGAAAGTGAGGTTTTGGGACTTTTGCATGAGTA
ATAGTTTCTCTCTCTGCATTCTAACTCTGGGCCAAGTTTGAGTGTGGAGACAAAGAGAAAAGT
TCTTGTGTACCAGTATACATAGCAAATGGTTTATGTGCTGAGGACTGCGGCTTGCTGGGTTTA
GTTGATATTTTCCCACGAGCCCCAGCTCATCCAAATTGGTAAATATGTGAGGTTTTCACTTTCA
ACATTTGTTACACTGTTTTGCACTTAGGTACATTGAAGGAAACTCAGACAAGTGGATTGTTTTG
AGTGCACAGTAAGGGCAGTTTGAGGAGAGATGGAGAGAATTATGTCCACCCACCTTTCAGG
CTAGATCCCCGGGTGGGTTCTTTTAAAGACTGGTCATTATGGGAAAGAAAAAGCAATCAGAA
TACTGGTCCAGTACATTTCTAAGCTTAACAAGGGTAATTGCATTTGGGAATATATTCTGTCTACT
CCTCCAAAAAACTAAATAAATAAATAGGGGAGAATGAATGGGTTAGAATAAACTTCCAAATTTT
ATGTGCTTTTAGAGGAAGTTTCACCTATAATTCTTATCAAATCTGTGTAAACGTTTTCTGTGTTGT
GAAATTCCTGAGTAAATGAGAATTTTGAGAGATAAAAATTGAGTAAACTTTTAAAAGTTTGATAAT
ATTTGTTATTTGGGTAATATCATATTTGTTTAAAGTTGTGTTACAATTAATAATGTTAAAAGGAAAT
GGCTTGTGGTTTTATCTTCTCCATGTCATCAGAGAAACATGGGCATGACTGAAACATTTAAGAT
CCTATATGCACTGTGTTAAAATGGATTCTTTCATATATCATATGTATGTATTGGGGCCTGTGATT
AATGTAAGGTATGCATATTGTTGTTTGGCTCTTGTTTACTATTTAGTGGGGCTGGCAGTATGGG
TCCGGGCTCTTATGGCAGTCCTCTTCCTTGGATGATTCCTTTGCCAAAAACTAAAAGGGGGA
GTAGGAACTCAGGGCAGATGGCACTAATTCTTCCTTTAACCGAAAGTTCCTGCAGGGACTC
CAGAAAGAAAACACAGAGGCCACTACTGATCATACTTCTCTTGTGTTTGTTTGGTCCATGTTTA
CTAAATATTCTTGCCAGATTCCTCTCTTCTAGGGTCGGAGCCATCAACTTCCAGATGACTGTA

TACCAAACCCAAGGTCTCTAAGTCCACTGAACCTGGCATCAGCCAGGTTTTGAAGTTTGACC
CTCGAATGGGACTCCAGAGAGGCAGGGACAGCTCTACGCCCCTTGCCAGCAGGAAGCAG
TTTCAGTAAAGCAGAGACCTCCGCCCCTTTTCACCAAAAGAATTTGGTTCCTATTTGTTTGAG
GGGGATGATGGGGACAGGGCCCAAACCAAGATCTTCGCTCTTTTGCCTCAAAGGGAATCTG
ACTCCACAGGCCCCAGACCCTCATCTCCCTTTGAAAGCCTTAGCCTTACCTTTTAATCTTATT
CAGATCTGGCTCTCTTTGTCTATAGGCCCCAGAAAAGGCCTTCCTTTATCTCCTTTTGAAAGT
CCTAGCCCCACCTTGATTTTGTGCAGATGCCTTTCAAACTTCTGCCTTCCTGTTTCCTCCATTA
TCTATTCCCACTGTCTATTCAATCTCCCTTGACCCCCTGCATTTCCCTATTTTCCACTACCCTC
CCTGTTTCACATGCCCCCACCTACTTGCCCATCCCCCTTTCCTTGGTTCTCCTTCCCTTTCCT
CTGGCTCCTAGTAGGACCCCTCAGGCAGATCCTCCTCACCCTCGATTATGAGACTGCTCC
GAGCCATCCATAGCGTTGTCAACTCCACCCGGCCTGACCTAGGACTCCAGCTGCTGGCTG
TGTATGGATGCACAAACCCCCACACTATGGTGGGAGTGGCAGTTAATGGTTCTCGGTTCCC
ATAGCCCAGACTCGGAAAATTGTCGGTGGGAACAACCTAAGTTGACCGTGGGGGATGTCCA
AGGAAAAGGTACCTGTCTTACCTCATCCACCACATCCCTGAAAACTTCTCCTTACTCTCCTGT
CTGTAATAGTACTGTAGTTGTTCCCCAATCCTCCTCCTCTTTATTCAGGGCCATCCGGTACCT
GGTGGGCCTGTCTGGATGGGGATCACACAGTGTGTCTCCGCTGCAGTGTTCCTAAAAGAAT
CTGAGCCTCTATGTATATTAGTCTCCATTATCCCCAGGGTCTCCCTTCTTCAGGGCCCGGAG
GGTTGGGAGTACTTCAGTCATGAGGGCATGTGTGTCCTACTGAAGCAAGCGAGCTGTTCCC
CTCCTCATCCCTATCCTCAGTGGGGTTGGGGTTAGTGGATCACCTGCTGTGGGTACCGCTG
CCCTAGTCCGAGGAGAGGCCAGTTACCAGGAACTTAGTGCTCAGGTCGACGAGACCTCAG
TCATCTGGAGCACTCTATATCCCAACTGAGAGACAGGTCAACTCCTTGGCGGAGATGGTTCT
GCAGAACCGGAGGGGTTTGGACTTATTGTTTCTCAGGCAAGGCGGCCTTTGTGCTGCCCTA
GGGAGGCCTGCTGTTTCTAGTGCCAATAATTCAGGTGTGGTTCAGGAGAGCCTTCTCCTCAG
TACGGAAAAAATATTGCAGACAGGCAGAGGGAACGTGGAAGGGAACCAAAATTGGTACCAG
AGCCTGTTTCGACTTCCTCATGGTTAACCACTCTTATTTCTGGCCTTAGCCGGGCCTTTCCTC
TTCACCATAGCCCTAATTCTTGGTCCTTGTCTAGTTAATCAGCTCCTTGACTTTGTTAGATCCC
GTATTAATTCAGTGTAAGTTACTGTTGGTTAGAGATCCCCGCGTATCAGCCCCTGGCGCCTG
CCTCCTTTACACCCTATGATGAGATAGTCCCCACCACGTCAAGGGTTTGACGTGGTACACAA
AAGAAGTGGGGAATGTAATGCAGAACATTATTTTACCATTTAGGTAGTAGGGCCCTTTAGGCA
ATGGAAAGCAGAAGTTGCTGCTTAATCTTGCCCCCAGACCTCTTTCCTCCTAAATCTAAAACA
ATAGTGAAATGTACTCCTGCATGGCTTAAGCACAGCCCATACCTCCAAAGCAAATGGATAAG
AAAACTTGCAATATCAATGGCTTTAGATAATTATGTTTATACCAGAAACCAGGACACCTGGTCT
GGTTTGTGTGATCCCTGACCTGTAATGATGGGGTGCTCGGGTGCTTGTGCTTATACTTTTGCC
CCAAAATGCCTCCCTAGTCTCAGAAACACTATCTCTGCAACCTGACTATACCCTTAAACGCC
TCACATACTCACAAGTACCTCCTCCCTCCAAACCACAGCTGGTGGGCTTGTAACTCTGGCC
TCACCCCTGCCTCTCTACATCAGTCTTCAACCAGTCTAACGATTTCTGTATCCAGATCCAGCT
TGTCCTCTGCATCTACTATCACCCAGACGGTACCTTGCTACAGGCCTATGAGTCCCCCACC
CTAGAAACAAGAGAGAGCCTGTCTCACTCACCCTGGCTGTCCTTCTCTTCGGATTAGGGGT
CGCAGCAGGTATAGGTACCGGCCTCGACCGCCTTCTAATAAAAGGGCCCATAGACCTCCA
ACAAGGTTTGACTAGCCTCCAGATTGCCATGGATACAGACCTTAGGGCCCTTCAAGACTCC
ATAAGTAAACTAGAGGATTCTCTTAAACTCCCTGTCTGGAAGTAGTGCTCCAGAATAGGAGAG
GCCTTGATCTGCTATTTTTGAAGGAAGGGGCCTTTGAGCAGCCTAAAGAGGAATGCTGTTTCT
ATGATATTGACCACTCCCAGGCGCGGTCGCGGGGGAGCTCCGTGAGGAGACTCAAGGAA
AGGTTAGATAAGAGGCAGTTAGAGCACCAAAAGAATTTAAGTTGGTACGAGGATGGTTCAAC
CGTTCCCCCTGGCTTACTACTTTACTGTCTGCCCTTGCTGGTCCCCTGCTACTCCTCCTTCT
GTTACTCACCCTCGGGCCTTGTGTCATCAATAAGTTAGTGCAATTCATCAATGATAGGGTTAG
TGCAGTAAGGATTCTGGTTCTCAGGCACAAGTACCAGACCCTAGACAACGAGGATAACCTTT

AATTCTGCTCTATGATTAGAGCTACCCTCCAAGAAAATGGGGAATGAAGGAGGCAGAAATCA
TGAGGCAGAAATCATTCCGTGAAGTATGGAAACTACCCGGAGAGCCCAAGGTTTAGGGACA
GGTGCAGCCAGGCACAGTAAAAGGTCAGAGCAAGAAAAACAAGGAAGATTTGGGTGCCAA
ACAGAATATCTGTGGTCATGCACCTGAGTCCCCACCCGGACTTATGCAAACAATTCCCAGA
AATAGCTGAGCTCATAACAGTTTCTAGGGTGCCCCTCAGCGGTTTCTAGAACCCTCTCGTGA
CCAGGTTTTTATTCAAACTAACCAATGATCTTGCTCCTCGCTTCTGTACCCGCGCTTTTTGCTA
TAAAATGAGACCAAAGAATCCACCCGGCGCGCCAGTCCCTCTAGGTGACTGAGTCGCCCA
GTACTCGTAAGTTCAATAAACCTCTTGCTATTTGCATCCGGAGTTGTGTTCGCGTTGATCCTG
GGAGGGTTTCTCAAGGTCGGTGGACTACCCGAACATCAGGGTCTTTCAT
>Koala-01-SA1_06
ATTGAAGGAGGCAGAAATCATGAGGCAGAAATCATTCCGTGAAGTATGGAAACTACCCGGA
GGGCCCAAGGTTTAGGGACAGGTGCAGCCAGGCACAGTAAAAGGTCAGAGCAAGAAAAC
AAGGAAGATTTGGAGTGCCAAACAGAATATCTGTGGTCATGCACCTGAGTCCCCACCCCGG
ACTTATGCAAACAATTCCCAGAAATAGCTGAGCTCATAACAGTTTCTAGGGTGCTCCTCAGC
AGTTTCTCTAGAACCCTCTCGTGACCGGAGTTTTTATTCAAACTAACCAATGATCTTGCTCCTC
GCTTCTGTACCCGCGCTTTTTGCTATAAAATGAGACCAAAGAATCACCCGGCGCGCCAGTC
CCTCTAGGTGACTGAGTCGCCCGAGTACTCGTAAGTTCAATAAACCTCTTGCTATTTGCATC
CGGAGTTGTGTTCGCGTTGATCCTGGGAGGGTTTCTCAAGGTCGGTGGACTACCCGAACAT
CGGGGTCTTTCATTTGGGGGCTCGTCCGGGATCTGAGATTCCTACCCGCGACCGCCGAAC
CACCGACGGGAGGTAAGCTGGCCAGCGATCGCTCTATGTTCTCTCTGTGTCTGACTCCGAA
AACTCTGACTGTCTATTCGGTGTGCGCGCATTTTTGGTTTCAGTTTATTCCAGGTTAATCGATC
TAAGATCGAGGCGTGAGTAGCGGACAGACGTGTTCGGGGCTCACCGCCCGGCAATCCTG
GGAGACGTCCCAGGATCAGGGAGGACCAGGGACGCCTGGTGGACCCCACGGCAAGGGA
TAATTCTCTTCTGATCTCACAGTTGCACTGTCTTGAGAGACATACCCTACCTTCTGACTCTTTTT
CCTGTCTTTAATACATACGCCTACGCCGCCATATAATTTTTTTTCCTTTTATTTCAATAATAATCA
TTATCTGGGTCGTAATTCCACTCTCCTTCGGGACCCCCCAGAATGGGGACAGGGTGAGTCG
ACCTCTCTCTCTCTACACTAGATCACTGGAAAGACGTGAAGACAAGGGCTCACAATCTTTCC
GTGGAGATAAGAAAGGAAAGTGGCAAACCTTTCTGTTCCTCCGAGTGGCCCACGGACATCG
GCCTCGGAGGGGGAATTGTTCTGTTCTGACTTCTAGGGGAGCCGGCCCCGATGATGTTCCA
GGGCCCCAGGACTCAGGACTAGGAGAAGCCTCCCTCCCCGACTCCGACTGTACCTCATTT
CGGCCATCGTGGACCTGGTAAACCTCTGAGGTAAATGTTTGCAAACCTGTTTTCTGGTGGGG
AACGAGTGCCAGACGTGGCAGAGCACCTAATTAGTGCCTCAATCCCCAGGCACCCGGAG
ACGTCGGGACTATGCCTGTATCTGGTTCCTGGTACCCGGTTTCTGGTTCCTGGTTTCTGTCTT
AGTTCTGTTGGGCCCGTGTCTCGTTCCTGGTTTCTGTCTCAGTGTTGGTCTATTAGTTTATATGT
GTAGAGATTGTGTTTTTAAGAGATTCTGTTTTGCAATCATCTGCCTGAAGCCACCTCTGTTTCAT
ACTGAAAGACAGGTGTCAGACTGGATTGCAGACAGTTTTCTCCAGACTTCCATTGGGGAGGA
TGTGTTTGTGTTCAGGCTTTTATTGTTTGTCTGTGTCTATGTTCTGCAATAATGGGGGATACGAC
CAGTACATCATTTCCTCCCTTTTGGGTTGTTTGCAGCCAGACAGCCAAAGGCTGCAGCAGG
ACTTCTGAAAAGTTGCCTTCATTCTCCCTCTTCCTCCCCAGGTAACAGCGGCTTCAAAGCAG
TGCGTGGATGGGAGAGGGAAAGAGAGAAGAATACTTTATTATTTAGGTTATATGGTTGGTAGA
GGGCTAGTTGGGGAAGGATTTTTGAAGTGTCAGGTCAGAATTAGAAAGGAATGAAATATGTAC
AGGTTTTTAAGGGATTTTAAATCATTAGGAAAGAAAGGCAAGGAAGTTTAAGCATGTAGGAGA
GAGGAGTGTGTGTGTAGAAAATTTGAAGGGAATATGATTTAGTTGCATGGTGTGACTGGAGGT
TTTGAAAGTGAGGTTTTGGGACCTTTGCATGAGTAATAGTTTCTCTCTCTGCATTCTAACTCTG
GCCAAGTTTGGAGTGTGGAGACAAAGAGAAAAGTTCTTGAGTACCAGAACATAGCAAATGGT
TTATGTGCTGAGGACTGCGGCTTGCTGGGTTTAGTTGATATTTTCCCCACGAGCCCCAGCTC
ATCCAAATTGGTAAATATGTGAGGTTTTCAATTTCAACATTTGTTACACTGTTTTGCACTTAGGTA

CATTGAAGGAAACTCAGACAAGTGGATTGTTTTGAGTGCACAGTAAGGGCAGTTTGAGGAGA
GATGGAGAGAATTATGTCCACTCCACCTTTCAGGCTAGATCCCCGGGTGGGTTCTTTTTTAAA
GACTGGTCATTATGGGAAAGAAAAAGCAATCAGAATACTGGTCCAGTACATTTCTAAGCTTAA
CAAGGGTATTGCATTTGGGAATATATTCTTGTCTACTCCTCCAAAAACTAAATAAATAAATAGG
GGAGAATGAATGGGTTAGAATAAACTTCCAAATTTTATGTGCTTTTAAGAGGAAGTTTCACCTAT
AATTTTTATCAAATCTGTGTAAACGTTTTCTGTGTTGTGAAATTCCTGAGTAAATGAGAGAATTCT
TGAGAGATAAAAATTGGAGTAAACTTTTAAAAAGTTTGATAATATTTGTTATTTGGGTAATATCATA
CTTGTTTAAAGTTGTGTTACAATTAATAATGTTAAAAGGAAATGGCTTGTGGTTAATCTTCTCCAT
GTCATCAGAGAAACATGGCATGACTGGAAACATTTAAGATCCTATATGCACTGTGTTAAAATG
GATTCTTTCATATATCTATGTATGTATTGGGGCCTGTGGATTAATGTAAGGTATGCATATTGTTGT
TTGCTCTTGTTTACTATTTAGTGGGGTTGGCAGTATGGGTCCGGCTCTTATGGCAGTCCTCTT
CCTTGATGATTCCTTTGCCAAAAACTAAAAGGGGGAGTAGGAACTCAGGGCAGATGGCACT
AATTTTCCCTAACCAAGTTCCTGCAGGGACTCCAGAAAGAAAACACAGAGGCCACTACTGAT
CATACTTCTCTTGTGTTTGTTTGGTCCATGTTTACTAAATATTCTTGCCAGATTCCTTTCTTCTAG
GGTCGGAGCCATCAACTCCAGATGACTGTATACCAACCCGAGGTCTCTAGTCCACTGAACC
TGGCATCAGCCAGGTTTTGAAGTTTGACCCCTCGAATGGGACCCAGAGAGGCAGGGACAG
CTCTACGCCCCTTGCCAGCAGGAAGCAGTTTCAGAAGCAGAGACCTCCGCCCCTTTTCAC
CAAAAAGAATTTGGGTTCCTATTTGTTTGAGGGGGATGATGGGGCGGGGCCCAAACCAAGA
TCTTCGCTCTTTGCCTCAAAGGGAATCTGACTCACAGGCCCCAGACCCCTCATCTCCCTTTT
GAAAGCCTTAGCCTTACCTTTTTAATCTTATTCAGATCTGGCTCCTTTTTGTCTATAGGCCCCA
GAAAGGCCTTCCTTTATCTCCTTTTGAAAGTCCTAGCCCCACCTTGATTTTGTGCAGATGCCT
TTCAAACTTCTGCCTTCCTGTTCCTCCATTATCTATTCCCACTGTCTATTCAATCTCCCTTGAC
CCCCCTGCATTTCCCTATTTTCCACTACCCCTCCCTGTTTCACATGCCCCCACCTACTTGCC
CATCCCCCTTTCTCTTGGTTCTCCTTTCCCTTTTCCTCCTGGCTCCTAGTAGGACCCCTCAG
GCAGATCCTCCTCACCCCTCGATTATGAGACTGCTCCGAGCCATCCATAGCGTTGTCAACT
CCACCCGGCCTGACCTAGGACCTAGCTGCTGGCTGTGTATGGATGCACAACCCCACACTA
TGTGGGAGTGGCAGTTAATGGTTCTGGTTCCCATAGCCCAGACTCGGAAAATTGTCGGTGG
GAACAACCTAAGTTGACCGTGGGGATGTCCATGGAAAAGGTACCTGTCTTACCTCATCCAC
CACATCCCTGAAAACTTCTCCTTACTCTCCTGTCTGTAATAGTACTGTGGTTGTTCCCCCAAT
CCTCCTCCTCTTTTTATTCAGGGCCATCCGGCACTTGGTGGGCCTGTCTGGATGGGATCAC
ACAGTGTGTCTCCGCTGCAGTGTTCCTAAAAGAATCTGAGCCTCTATGTATATTAGTCTCCATT
ATCCCCAGGGTCTCCTCTTCTTCAGGGCCCGGGAGGGTTGGGGAGTACTTCAGTCATGAG
GGCAGTGTGTCCTACTGAAGCAAGCGAGCTGTTCCCTCCCTCATCCCTATCCTCAGTGGGG
TTGGGGTTAGTGGGATCACCTGCTGTGGGTACCGCTGCCCTAGTCCGGAGGAGAGGCCAG
TTACCAGGAACTTAGTGCTCAGGTCGACGTAGACCTCAGTCATCTGGAGCACTCTATATCCC
AACTGGAGAGACAGGTCAACTCCTTATGGAGATGGTTCTGCAGAACCGGAGGGTCTTGGAC
TTATTGTTTCTCAGGCAAGGCGGCCTTTGTGCTGCCCTAGGGAGGCCTGCTGTTTCTATGCC
AATAATTCAGGTGTGGTTCAGGAGAGCCTTTCCCTAGTACGGAAAAATATTGCAGACAGGCA
GAGGGAACGGGAAGGGAACGAAAATTGGTACCAGAGCCTGTTTCGGACTTCCCCATGGTTA
ACCACTCTTATTTCTGCCTTAGCTGGCCTTTCCTCCTTACTATAGCCCTAATTCTTGGTCCTGT
CTAGTTAATCGGCTCCTGACTTTGTTAGATCCTGTATTAATTTAGTTAAGTTACTGTTGGTTAAAG
GTCCTCGTTATCAGACCCTGGTGTCTGCCTTCTTTGCACCCTATAATGAGGTAGTCTCCAAA
CCACGTCAAGGGTTTGACGTGGTACACAAAAGAAGTGGGGAATGTAATGCAGAACATTATTT
TACCATTTAGGTAGTAGGGCCCTTTAGGCAATGGGAAAGCAGAAGTTGCTGCTTAATCTTGC
CTCGCACCTCTTTCCTCCTAAATCTAAACAATAGTGAAATGTACCCTGCATGGCTTAAGCACA
GCCCATACCTCCAAAGCAAAATGGATAAGAAAACTTGCAATACAATGGCTTTAGATAATTATG
TTTATACCAGAAACCAGGACACCTGGTCTGGTTTGTGTGATCCCTGACCTGTAATGATGGGG

TGCTTCGGGTGCTTGTGCTTATACTTTTGCCCCAAAATGCCTCCCTAGTCTCAGAAACACTAT
CTCTGCAACCTGACTATACCCTTAAACGCCTCACATACTCACAAGTACCTCCTCCCTCCAAC
CAACAGCTGGTGGGCTTGTAATTCTGGCCTCACCTCCTGCCTCTCTACATCAGTCTTCAACC
AGTCTAACGATTTCTGTATCCAGATCCAGCTTGTCCCTCGCATCTACTATCACCCAGACGGT
ACCTTGCTACAGGCCTATGAGTCCCCCACCCTAGAAACAAGAGAGAGCCTGTCTCACTCA
CCCTGGTTGTCCTTCTCGGATTAGGGGTCGCAGCAGGTATAGGTACCGGCTCGACCGCCC
TAATAAAAGGGCCCATAGACCTCCAACAAGGTTTGACTAGCCTCCAGATTGCCATGGATACA
GACCTTAGGGCCCTTCAAGACTCCATAAGTAAACTAGAGGGATTCCTTAACCTCCCTGTCTG
AAGTAGTGCTCCAGAATAGGAGAGGCCTTGATCTGCTATTTTTTGAAGGAAGGGGGCCTTTG
TGTGCAGCCCAAAAGAGGAATGCTGCTTCTATGTTGACCACTCAGGCGCGGTGCGAAGAC
CCATAGGGGCGACTCCAAGGAAAGGTTAGATAAGAGGCGGTTAGAGCACCCAAAAGAATTT
AAGTTGGTACGAGGGATGTGGTTCAAACCGTTCCCCTGGCTTACTACTTTACTGTCTGCCCTT
GCTGGTCCCCTGCTACTCCTCCTTCTGTTACTCACCCTCGGGCCTTGTGTCATCAATAAGTT
AGTGCAATTCATCAATGATAGGGTTAGTGCAGTAAGGATTCTGGTTCTCAGGCACAAGTACCA
GACCCTAGACAACGAGGATAACCTTTAATTCTGCTCTATGATTAGAGCTACCCTCCAAAGAA
AATGGGGAATGAAGGAGGCAGAAATCATGAGGCAGAAATCATTCCGTGAAGTATGGAAACT
ACCCGGAGGGCCCAAGGTTTAGGGACAGGTGCAGCCAGGCACAGTAAAAGGTCAGAGCA
AGAAGAAAAACAAGGAAGATTTGGAGTGCCAAACAGAATATCTGTGGTCATGCACCTGAGTC
CCCACCCGGACTTATGCAAACAATTCCCAGAAATAGCTGAGCTCATAACAGTTTCTAGGGTG
CCCCTCAGCAGTTTCTAGAACCCTCTCGTGACCGGAGTTTTTATTCAAACTAACCAATGATCT
TGCTCCTCGCTTCTGTACCCGCGCTTTTGCTATAAAATGAGACCAAAGAATCCACCCGGCG
CGCCAGTCCCTCTAGGTGACTGAGTCGCCCGAGTACTCGTAAGTTCAATAAACCTCTTGCTA
TTTGCATCCGGAGTTGTGTTCGCGTTGATCCTGGGAGGGTTTCTCAAGGTCGGTGGACTACC
CGAACATCGGGGTCTTTCAA
>Koala-03-Vic23
ACTGAAGGAGGCAGAAATCATGAGGCAGAAATCATTCCGTGAAGTATGGAAACTACCCGGA
GGGCCCAAGGTTTAGGGACAGGTGCAGCCAGGCACAGTAAAAGGTCAGAGCAAGAAAAA
CAAGGAAGATTTGGAGTGCCAAACAGAATATCTGTGGTCATGCACCTGAGTCCCCACCCCG
GACTTATGCAAACAATTCCCAGAAATAGCTGAGCTCATAACAGTTTCTAGGGTGCCCTCAGC
AGTTTCTAGAACCCTCTCGTGACCGGAGTTTTTATTCAAACTAACCAATGATCTTGCTCCTCG
CTTCTGTACCCGCGCTTTTTGCTATAAAATGAGACCAAAGAATCCACCCGGCGCGCCAGTC
CCTCTAGGTGACTGAGTCGCCCGAGTACTCGTAAGTTCAATAAACCTCTTGCTATTTGCATC
CGGAGTTGTGTTCGCGTTGATCCTGGGAGGGTTTCTCAAGGTCGGTGGACTACCCGAACAT
CGGGGTCTTTCATTTGGGGGCTCGTCCGGGATCTGAGATTCCCACCCAAGGACCGCCGAA
CCACCGACGGGAGGTAAGCTGGCCAGCGATCGCTCTATGTTCTCTCTGTGTCTGACTCCGA
AAACTCTGACTGTCTATTCGGTGTGCGCGCATTTTTGGTTTCAGTTTATTCCGGGTTAATCGAT
CTAAGATCGAGGCGTGAGTAGCGGACAGACGTGTTCGGGGGCTCACCGCCCGGCAATCC
TGGGAGACGTCCCAGGATCAGGGAGGACCAGGGACGCCTGGTGGACCCCACGGCAAGG
GATAATTCTCTTCTGATCTCACAGTTGCACTGACTTGAGAGACATACCCTACCTTCTGACTCTT
TTTCCTGTCTTTTTAATATACGTCTACGCCGCCATATAATTTTTTTTTTTTTTTCCTTTTTATTTCAAT
AATAATCATTATCTGGGTCGTAATTCCACTCTTCGGGACCCCCAGAATGGGACAGGGTGAGT
CGACCCCTCTCTCTCTTACACTAGATCACTGGAAAGACGTGAAGACAAGGGCTCACAATCTT
TCCGTGGAGATAAGAAAGGGAAAGTGGCAAACCTTCTGTTCCTCCGAGTGGCCCACGTTCG
AAGTGGGATGGCCACCGGAGGGGACTTTTAATCCTTCTATTATTTCTGCAGTCAAAAGGATTG
TCTTCCAGGAGACTGGAGGACACCCGGACCAGGTTCCCTACATCATAGTTTGGCAGGACCT
CTCCAACAGCCCCCATGGGTGCCACCCTTAGCCAAGATCGCCGTTGCCTCTGGTCAAGAT
AACGGGCGAAAGTCGGCGGGGGGAGGCCGTCCGCTCCTTCCCCGGCTCCCCATCTACC

CCGGAGACGGACAGCCTGTTCCCTCCTCTCAGAACCCCCGCCCTATCCAACATCCCCTC
CCTGTCCTGTATACTCCTATATAAACCTAGCTCAACCCCTGATGGGGGCTGCTTGATTTGGAT
GTGAATATCCCTGGCAGCCGCGGCTATAGCTAATAAAGTCCACAATTTAAAATTAGCTCGGG
TCGTCAACTCGCTCATTTCTCGGTATAACATTTTGGAGGTTCCACCGAGATAGCAGCGTACC
GTGTCGCTGCAGAGACCCACCTGGAGAGGACATCGGCCTCGGGAGGGAATTGTTCTGTTC
TGACTTCTAGAGGGCCGGCCCCTGATGATGTTCAGGGCCCCAGGACTCAGGACTCAGGAG
AAGCCTCCCTCCCCGACTCCGACTGTACCTCATTTCGGCCATCGTGGACCTGGTAACCCT
CTGGAGGTAAATGTTTGCAAACCTGTTTTCTGGTGGGGAACGAGTGCCAGACGTGGCAGAG
CACCTAATTAGTGCCTCAATCCCCAGGCACCCCGAGACGTCGGGACTATGCCTGTATCTG
GTTCCTGGTACCCGGTTTCTGGTTCCTGGTTTCTGTCTTAGTTCTATTGGGCCCGTGTCCCGT
TCCTGGTTTCTGTCTCAGTGTTGGTCTATTAGTTTATATGTGTAGAGATTGTGTTTTTAAGAGATT
CTGTTTTGCAATCATCTGCCTGAAGCCACCTCTGTTTCATACTGAAAGACAGGTGTCAGAGTA
GATTGCAGACAGTTTTCTCCAGGGCAGTTCCTTTGACTTCCATTGGGAGGATGTGTTAGTGTT
CAGGCTCTTATTGTTTGTCTGTGTCTGTGTTCTGCAATAATGGGGGATACGACCAGTACATCA
TTTCCTCCCTTTTGGGTTGTTTGCAGCCAGACAGCCAAAGGCTGCAGCAGGACTCTGAAGA
GTTGCCTTCATTCTCCCCCTCTTCCTCCCCAGGTAACAGCGGCTTCAAAGCAGTGCGTGGA
TGGGAGAGGGAAAGAGAGAAGAATACTTTATTATTTAGGTTATATGGTTGGTAGAGGGCAAGT
GTGGGAAGGATTTTTGAAGTGTCAGGTCAGAATTAGAAAGGGATGAGATATGTACAGGTTTTA
AGGGGATTTTAAATCATTAGAAAGAAAGGCAAGGAAGTTTAAGCATGTAGGAGAGAGGAGTG
TGTAGAAAATTTGAAGGGAATATGATTTAGTTGCATGGTGTGACTGGAGGTTTTGAAAGTGAGG
TTTGGGACTTTGCATGAGTAATAGTTTCTCTCTCTCTCTGCATTCTAACTCTGGCCAAGTTTGA
GTGTGGAGACAAAGAAAAGTTCTTGTGTACCAGAACATAGCAAATGGTTTATGTGCTGAGGA
CTGCGGCTTGCTGGGTTTGATTGATATTTTCCCACGAGCCCCAGCTCATCAGAATTGGTAAAT
ATGTGAGGTTTTCAATTTCAACATTTGTTACACTGTTTTGCATAGGTACATTGAAGGAAACTCAG
ACAAGTGGATTGTTTTGAGTGCACAGTAAGGGCAGTTTGAGGAGAGATGGAGAGAATTATGT
CCACCCACCTTTCAGGCTAGATCCCCGGGTGGGTTCTTTTAAAGACTGGTCATTATGGGAAA
GAAAAAGCAATCAGAATACTGGTCCAGTACATTTCTAAGCTTAACAAGGTGTGTGCATTTGGG
AATATATTCTTGTCTACTCCTCCAAAAACTAAATAAATAAATAGGGAGAATGAATGGGTTAGAAT
AAACTTCCAAATTTTATGTGCTTTTAAGAGAGCTTCACCTATAATTCTTATCAAATCTGTGTAAAC
GTTTTCTGTGTTGTGAAATTCCTGAGTAAATGAGAATTCTTGAGAGATAAAAATTGAGTAAACTT
TTAAAAGTTTGATAATATTTGTTATTTGGGTAATATCATACTTGTTTAAAGTTGTGTTACAATTAATA
ATGTTAAAAGGAAATGGCTTGTGGTTTATCTTCTCCATGTCATCAGAGAAACATGGCATGACT
GAAACATTTAAGATCCTATATGCACTGTGTTAAATGGATTCTTTCATATATCTATGTATGTATTGG
GGCCTGTGGATTAATGTAAGGTATGCATATTGTTGTTTGCTCTTGTTTACTATTTAGTGGGGGTT
GGCAGTATGGGTCTGGCTCTTATGGCAGTCCTCTTCCTTGGATGATTCCTTTGCCAAAAACTA
AAAGGGAGTAGGAACTCAGGGCAGATGGCACTAATTCTTCCTTAACCAAGTTCCTGCAGGG
ACTCCAGAAAGAAAACACAGAGGCCACTACTGATCATACTTCTCTTGTGTTTGTTTGGTCCAT
GTTTACTAAATATTCTTGCCAGATTCCTTTCTTCTAGGTCGGAGCCATCAACTTCCAGATGACT
GTATACCAACCCAAGGTCTCTAGTCCACTGAACCTGGCATCAGCCAGGCTTTGAAGTTGAC
CCCTCGAATGGGACCCAGAGAGGCAGGGACAGCTCTACGCCCCTTGCCAGCAGGAAGC
AGTTTCAGAAGCAGAGACCTCCGCCCCTTTTCACCAAAAGAATTTGGTTCCTATTTGTTTGAG
GGGGGATGATGGGGGGCGGGGCCCAAACCAAGATCTTCGCTCTTTGCCTCCAAGGGAAT
CTGACTCACAGGCCCCAGACCCTCATCTCCCTTTGAAAGCCTTAGCCTTACCTTTTAATCTTA
TTCAGATCTGGCTCCCTTTTGTCTATAGGCCCCAGAAAGGCCTTCCTTTATCTCCTTTTGAAA
GTCCTAGCCCCCACCTTGATTTTGTGCAGATGCCTTTCAAACTTCTGCCTTCCTGTTCCCTCC
ATTATCTATTCCCACTGTCTATTCAATCTCCCTTGACCCCCCTGCATTTCCCTATTTTCCGCTA
CCCCTCCCTGTTTCACATGCCCCCACCTACTTGCCCATCCCCCCTTTTCCTTGGTTCTCCCT

TCCCTTTTCCTCTGGCTCTAGTAGGACCCCTCAGGTCAGATCCTCCTCACCCCTCGATTATG
AGACTGCTCCGAGCCATCCATGGCGTTGTCAACTCCACCCGGCCTGACCTAGGACCTAGC
TGCTGGCTGTGTATGGATGCACAACCCCACACTATGTGGGAGTGGCAGTTAATGGTTCTGGT
TCCCATAGCCCAGACTCGGAAAATTGTCGGAGTGGGAACAACCTAAGTTGACCGTGGGGG
ATGTCCAGGAAAAAGGTACCTGTCTTACCTCATCCACCACATCCTGAAAACTTCTCCTTACTC
CTGTCTGTAATAGTACTGTGGTTGTTCCCCAATCCTCCTCCTCTTTTATTCAGGGCCATCCGG
CACTTGGTGGGCCTGTCTGGATGGGATCACACAGTGTGTCTCCGCTGCAGTGTTCTAAAAG
AATCTGAGCCTCTATGTATATTAGTCTCCATTATCCCCAGGGTCTCCTTCTTCCAGGGCCCG
GAGGGTTGGGAGTACTTCAGTCATGAGGGCAGTGTGTCCTACTGAAGCAAGCTGAGCTGTT
CCCCTCCTCATCCCTATCACTCAGTGGGGTTGGGGTTAGTGGGATCACCTGCTGTGGTACC
GCTGCCCTAGTCCGAGGAGAGGCCAGTTGCCAGGAACTTAGTGCTCAGGTCGACGTAGAC
CTCAGTCATCTGGAGCACTCTATATCCCAACTGGAGAGACAGGTCAACTCCTTGGCGGAGA
TGGTTCTGCAGAACCGGAGGGTTTGGACTTATTGTTTCTCAGGCAAGGCGGCCCTTGTGCTG
CCCTAGGGAAGCCTGCTGTTTCTATGCCAATAATTCAGGTGTGGTTCAGGAGAGCCTTTCCC
TAATGCGGAAAAATATTGCAGACAGGCAGAGGGAACGGGAAGGGAACGAAAATTGGTACCA
GAGCCTGTTTCGGACTTCCCCATGGTTAACCACTCTTATTTCTGCCTTAGCGGGCCTTTCCTC
TTCACCATAGCCTAATTCTTGGTCCTTGTCTGAGTTAATCAGCTCCTTGACTTTGTTAGATCCC
GTATTAATTCAGGTAAGTTACTGTTGGTTAGAGATCCCCGCTATCAGCCCCTGGCGTCTGCC
TCCTTACACACCCTATGATGAGACAGCCCCCACCACGTCAAGGGTTTGACGTGGTACACAA
AAGAAGTGGGGAATGTAGTGCAGAACATTATTTTACCATTTAGGTAGTAGGGCCCTTTAGGCA
ATGGAAAGCGCAGAAGTTGCTGCTTAATCTTGCTGAGACCTCTTTCCTCCTAAATCTAAACAA
TAGTGAAATGTACTCCTGCATGGCTTAAACACAGCCCATACCTCCAAAGCAAATGGATAAGA
AAACTTGCAATACAATGGCTTTAGATAATTATGTTTATACCAGAAACCAGGACACCCTGGTCT
GGTTTGTGTGATCCCTGACCTGTAATGATGGGGTGCTCGGGTGCTTGTGCTTATACTTTGTGC
CCCTAAATGCCTCCTAGTCTCAGAAACACTATCTCTGCAACCTGACTATACCCTTAAACGCC
TCACATACTCACAAGTACCTCCTCCCCTCCAACCACAGCTGGTGGGCTTGTAATTCTGGCC
TCACCCCTGCCTCTCTACATCAGTCTTCAACCAGTCTAACGATTTCTGTATCCAGATCCAGCT
TGTCCCTGCATCTACTATCACCCAGACGGTACCTTGCTACAGGCCTATGGAGTCCCCCACC
CTAGAAACAGAGAGCCTGTCTCACCCACCTGGCTGTCCTTCGGATTAGGGGGTCGCAGCA
GGTATAGGTACCGGCTCGACCGCCTAATAAAGGGCCCATAGACCTCCAACAAGGTTTGACT
AGCCTCCAGATTGCCATGGATACAGACCTTAGGGCCCCTTCAAGACTCCATAGAGTAAACT
AGAGGATTCCTTAACCTCCCTGTCTGAAGTAGTGCTCCAGAATAGGAGAGGCCTTGATCTGC
TATTTTTTGAAGGAAGGGGGCCTTTGTGCAGCCCTAAAGAGGAATGCTGTTTCTATGTTGACC
ACTCAGGCGCGGTGCGAGGACTCCATGAGGAGACTCAAGAAAGGTTAGATAAGAGGCAGT
TAGAGCACCAAAAGAATTTAAGTTGAGTACGAGGGATGGTTCAACCGTTCCCCTGGCTTACT
ACTTTACTGTCTGCCCTTGCTGGTCCCCTGCTACTCCTCCTTCTGTTACTCACCCTCGGGCC
TTGTGTCATCAATAAGTTAGTGCAATTCATCAATGATAGGGTTAGTGCAGTAAGGATTCTGGTT
CTCAGGCACAAGTACCAGACCCTAGACAACGAGGATAACCTTTAATTCTGCTCTATGATTAG
AGCTACCCTCAAAAGAAAATGGGGGAATGAAGGAGGCAGAAATCATGAGGCAGAAATCATT
CCGTGAAGTATGGAAACTACCCGGAGGGCCCAAGGTTTAGGGACAGGTGCAGCCAGGCA
CAGTAAAAGGTCAGAGCAAGAAAAACAAGGAAGATTTGGAGTGCCAAACAGAATATCTGTGG
TCATGCACCTGAGTCCCCACCCCGGACTTATGCAAACAATTCCCAGAAATAGCTGAGCTCA
TAACAGTTTCTAGGGTGCCCCTCAGCAGTTTCTAGAACCCTCTCGTGACCGGAGTTTTTATTC
AAACTAACCAATGATCTTGCTCCTCGCTTCTGTACCCGCGCTTTTTGCTATAAAATGAGACCA
AAGAATCCACCCGGCGCGCCAGTCCCTCTAGGTGACTGAGTCGCCCGAGTACTCGTAAGT
TCAATAAACCTCTTGCTATTTGCATCCGGAGTTGTGTTCGCGTTGATCCTGGGAGGGTTTCTC
AAGGTCGGTGGACTACCCGAACATCGGGGTCTTTCAA

>Koala-04-Vic31
CTTGAAGGAGGCAGAAATCATGAGGCAGAAATCATTCCGTGAAGTATGGAAACTACCCGGA
GGGCCCAAGGTTTAGGGACAGGTGCAGCCAGGCACAGTAAAAGGTCAGAGCAAGAAAAA
CAAGGAAGATTTGGAGTGCCAAACAGAATATCTGTGGTCATGCACCTGAGTCCCCACCCCG
GACTTATGCAAACAATTCCCAGAAATAGCTGAGCTCATAACAGTTTCTAGGGTGCCCCTCAG
CAGTTTCTAGAACCCTCTCGTGACCGGAGTTTTTATTCAAACTAACCAATGATCTTGCTCCTC
GCTTCTGTACCCGCGCTTTTTGCTATAAAATGAGACCAAAGAATCCACCCGGCGCGCCAGT
CCCTCTAGGTGACTGAGTCGCCCGAGTACTCGTAAGTTCAATAAACCTCTTGCTATTTGCAT
CCGGAGTTGTGTTCGCGTTGATCCTGGGAGGGTTTCTCAAGGTCGGTGGACTACCCGAACA
TCAGGGTCTTTCATTTGGGGCTCGTCCGGGATCTGAGATTCCCACCCAAGGACCGCCGAA
CCACCGACGGGAGGTAAGCTGGCCAGCGATCGCTCTATGTTCTCTCTGTGTCTGACTCCGA
AAACTCTGACTGTCTATTCGGTGTGCGCGCATTTTTGGTTTCAGTTTATTCCGGGTTAATCGAT
CTAAGATCGAGGCGTGAGTAGCGGACAGACGTGTTCGGGGGCTCACCGCCCGGCAATCC
TGGGAGACGTCCCAGGATCAGGGAGGACCAGGGACGCCTGGTGGACCCCACGGCAAGG
GATAATTCTCTTCTGATCTCACAGTTGCACCTGACTTGAGAGACATACCCTACCTTCTGACTC
TTTTTCCTGTCTTTTTAATATACGTCTCACGCCGCCATATACTTTTTTTTTTTTTTTTTTTTCCTTT
TTATTTCAATAATAATCATTATCTGGGTCGTAATTCCACTCTTCGGGACCCCCAGAATGGGAC
AGGGTGAGTCGACCCTCTCTCTCTTACACTAGATCACTGGAAAGACGTGAAGACAAGGGCT
CACAATCTTTCCGTGGAGATAAGAAAGGGAAAGTGGCAAACCTTCTGTTCCTCCGAGTGGC
CCACGTTCGAAGTGGGATGGCCACCGGAGGGGACTTTTTAATCCTTCTATTATTTCTGCAGT
CAAAAGGATTGTCTTCCAGGAGACTGGAGGACACCCGGACCAGGTTCCCTACATCATAGTT
TGGCAGGACCTCTTCAACAGCCCCTCCATGGGTGCCACCCTTAGCCAAGACATCGCCGTT
GCCTCTGGTCAAGATAACGGGCGAAAGTCGGCGGGGGAGGCCGTCCGCTCCTTCCCGG
CTCCCCATCTACCCGGAGACGGACAGCCTGTTCCTCCTCTCAGAACCCCGCCCTATCCAA
CATCCCCTCCCTGTCCTGTATACTCCCTATATAAACCCTAGCTCAACCCTGATGGGGGCTG
CTTGATTTGGATGTGAATATCCCTGGCAGCCGCGCGCACAATAGCTAATAAAGTCCACAATT
TAAAATTTAGCCTCGGGTCGTCAACTCGCTCATTTCTCGGTATAACATTTTGGAGGCCCTAGC
GAGATAGCAGCGTGCCGTGTCGCTGCAGAGACCCACCTGGAGAGGACATCGGCCTCGGG
AGGGGAATTGTTCTGTTCTGACTTCTAGGGAGCCGGCCCCTGATGATGTTCCAGGGCCCCA
GGACTCAGGACTAGGAGAAGCCTCCTCCCCGACTCCGACTGTACCTCATTTCGGCCATCG
TGGACCTGGTAACCCTCTGGAGGTAAATGTTTGCAAACCTGTTTTCTGGTGGGGAACGAGTG
CCAGACGTGGCAGAGCACCTAATTAGTGCCTCAATCCCCCAGGCACCCCGAGACGTCGG
GACTATGCCTGTATCTGGTTCCTGGTACCCGGTTTCTGGTTCCTGGTTTCTGTCTTAGTTCTGT
TGGGCCCGTGTCTCGTTCCTGGTTTCTGTCTCAGTGTTGGTCTATTAGTTTATATGTGTAGAGA
TTGTGTGTTTTAAGAGATTCTGTTTTGCAATCATCTGCCTGAAGCCACCTCTGTTTCATACTGAA
AGGCAGGTGTCAGAGTAGATTGCAGACAGTTTTCTCCAGGGCAGTTCCTTTGACTTCCATTG
GGAGGATGTGTTAGTGTTCAGGCTCTTATTGTTTGTCTGTGTCTGTGTTCCTGCAATAATGGGG
GATACGACCAGTACATCATTTCCCTCCCTTTTTGGGTTGTTTGCATGCCAGACAGCCAAAGG
CTGCAGCAGGACTCTGAAAAGTTGCCTTCATTCTCCCCTCTTCCTCCCCAGGTAACAGCGG
CTTCAAAGCAGTGCGTGGATGGGAGAGGGAAAGAGAGAAGAATACTTTATTATTTAGGTTATA
TGGTTGGTAGAGGGCAAGTTGGGAAGGATTTTTGAAGTGTCAGGTCAGAATTAGAAAGGGAT
GAGATATGTACAGGTTTTAAGGGATTTTAAATCATTAGAAAGAAAGGCAAAGGAAGTTTAAGCA
TGTAGGAGAGAGGAGTGTGTGTAGAAAATTTGAAGGGAATATGATTTAGTTGCATGGTGACTG
GAGGTTTTAGAAAGTGAGGTTTTGGGACTTTTGCATGAGTAATAGTTTCTCTCTCTCTCTGCATT
CTAACTCTGGCCAAGTTTGAGTGTGGAGACAAAGAGAAAAGTTCTTGTGTACCAGAACATAG
CAAATGGTTTATGTGCTGAGGACTGCGGCTTGCTGGGTTTAGTTGATATTTTCCCACGAGCCC
CAGCTCATCCAAATTGGTAAATATGTGAGGTTTTCAATTTCAACATTTGTTACACTGTTTTGCAC

TTAGGTACATTGAAGGAAACTCAGACAAGTGGATTGTTTTGAGTGCACAGTAAGGGCAGTTTG
AGGAGAGATGGAGAGAATTATGTCCACCCACCTTTCAGGCTAGATCCCCGGGTGGGTTCTT
TTAAAGACTGGTCATTATGGGAAAGAAAAAGCAATCAGAATACTGGTCCAGTACATTTCTAAG
CTTAACAAGGGTATTGCATTTGGGAATATATTCTTGTCTACTCCTCCAAAAACTAAATAAATAAA
TAGGGAGAATGAATGGGTTAGAATAAACTTCCAAATTTTATGTGCTTTTAAGAGGAAGTTTCAC
CTATAATTCTTATCAAATCTGTGTAAACGTTTTCTGTGTTGTGAAATTCCTGAGTAAATGAGAATT
CTTGAGAGATAAAAATTGAGTAAACTTTTTAAAAAGTTTGATAATATTTGTTATTTGGGTAATATCA
TACTTGTTTAAAGTTGTGTTACAATTAATAATGTTAAAAAGGGAAATGGCTTGTGGTTTATCTTCT
CATGTCATCAAAGAGAAACATGGCATGACTGAAACATTTAAGATCCTATATGCACTGTGTTAAA
ATGGATTCTTTCATATATCTATGTATGTATTGGGGCCTGTGGATTAATGTAAGGTATGCATATTG
TTGTTTGCTCTTGTTTACTATTTAGTGGGGGGTTGGCAGTATGGGTCCAGCTCTTATGGCAGTC
CTCTTCCTTGGATGATTCCTTTGCCAAAAACTAAAAGGGGGAGTAGGAACTCAGGGCAGATG
GCACTAATTCTTCCTTAACCAAGTTCCTGCAGGGACTCCAGAAAGAAAACACAGAGGCCAC
TACTGATCATACTTCCTTCTCTTGTGTTTGTTTGGTCCATGTTTACTAAATATTCTTGCCAGATTC
CTTTCTTCTAGGGTCGGAGCCATCAACTTCCAGATGACTGTATACCAACCCAAGGTCTCTAG
TCCACTGAACCTGGCATCAGCCAGGTTTTGAAGTTTGACCCCTCGAATGGGACCCAGAGAG
GCAGGGACAGCTCTACGCCCCTTGCCAGCAGGAAGCAGTTTCAGAAGCAGAGACCTCCG
CCCCTTTTCACCAAAAGAATTTGGTTCCTATTTGTTTGAGGGGGGATGATGGGGGCGGGGCC
CAAACCAAGATCTTCGCTCTTTGCCTCAAAGGGAATCTGACTCACAGGCCCCAGACCCTCA
TCTCCCTTTGAGAAGCCTTAGCCTTACCTTTTAATCTTATTCAGATCTGGCTCCTTTTTGTCTAT
AGGCCCCAGAAAGGCCTTCCTCTTATCTCCTTTTGAAAGTCCTAGCCCCACCTTGATTTTGTG
CAGATGCCTTTCAAACTTCTGCCTTCCTGTTCCCTCCATTATCTATTCCCACTGTCTATTCAAT
CTCCCTTGACCCCCCTGCATTTCCCTATTTTCCACTACCCCCTCCCTGTTTCACATGCCCTC
ACCCTACTTGCCCATCCCCTTTTCCTTGGTTCTCCTTCCCTTTTCCTCTGGCTCCTAGTAGGA
CCCCTCAGGCAGATCCTCCTCACCCCTCGATTATGAGACTGCTCCGAGCCATCCATAGCG
TTGTCAACTCCACCCGGCCTGACCTAGGACCTAGCTGCTGGCTGTGTATGGATGCACAACC
CCCACACTATGTGGGAGTGGCAGTTAATGGTTCTGGTTCCCATAGCCCAGACTCGGAAAATT
GTCGGTGGGAACAACCTAAGTTGACCGTGGGGGATGTCCAAGGAAAAGGTACCTGTCTTAC
CTCATCCACCACATCCCTGAAAACTTCTCCTTACTCTCCTGTCTGTAATAGTACTGTGGTTGTT
CCCCAATCCTCCTCCTCTTTTTATTCAGGGCCATCCGGCACTTGGTGGGCCTGTCTGGATG
GGATCACACAGTGTGTCTCCGCTGCAGTGTTCCTAAAAGAATCTGAGCCTCTATGTATATTAG
TCTCCATTATCCCCAGGGTCTCCCTTCTTCAGGGCCCGGAGGGTTGGGAGTACTTCAGTCA
TGAGGGCAGTGTGTCCCTACTGAAGCAAGCGAGCTGTTCCCCTCCTCATCCCTATCCTCAG
TGGGGTTGGGGTTAGTGGGATCACCTGCTGTGGGTACCGCTGCCCTAGTCCGAGGAGAGG
CCAGTTACCAGGAACTTAGTGCTCAGGTCGACGTAGACCTCAGTCATCTGGAGCACTCTAT
ATCCCAACTGGAGAGACAGGTCAACTCCTTGGCGGAGATGGTTCTGCAGAACCGGAGGGG
CTTGGACTTATTGTTTCTCAGGCAAGGCGGCCTTTGTGCTGCCCTAGGGGAGCCTGCTGTTT
CTATGCCAATAATTCAGGTGTGGTTCAGGAGAGGCCTTTCCCTAGTACGGGAAAAATATTGC
AGACAGGCAGAGGGAACGGGAAGGGAACGAAATTGGTACCAGAGCCTGTTTCGGACTTCC
CATGGTTAACCACTCTTATTTCTGCCTTAGCCGGGCCTTTCCCTCTTCACCATAGCCCTAATT
CTTGGTCCTTGTCTAGTTAATCAGCTCCTTGACTTTGTTAGATCCCGTATTAATTCAGGTAAGTT
ACTGTTGGTTAGAGATCCCCGCTATCAGCCCCCTGGCGTCTGCCTCCTTACACCCTATGAT
GAGACAGCCCCCACCACGTCAAGGGGTTTGACGTGGTACACACAAAGAAGTGGGGAATGT
AATGCAGAACATTATTTTACCATTTAGGTAGTAGGGCCCTTTAGGCAATGGAAAGCAGAAGTT
GCTGCTTAATCTTGCCCCAGACCTCTTTCCTCCTAAATCTAAACAATAGTGAAATGTACTCCT
GCATGGCTTAAGCACAGCCCATACCTCCAAAGCAAATGGATAAGAAAACTTGCAATACAATG
GCTTTAGATAATTATGTTTATACCAGAAACCAGGACACCTGGTCTGGTTTGTGTGATCCCTGA

CCTGTAATGATGGGGTGCTCGGGTGCTTGTGCTTATACTTTTGCCCCAAAATGCCTCCCTAG
TCTCAGAAACACTATCTCTGCAACCTGACTATACCCTTAAACGCCTCACTATACTCACAAGTA
CCTCCTCCCCACCAACCACAGCTGGTGGGCTTGTAATTCTGGCCTCACCCCCTGCCTCTC
TACATCAGTCTTCAACCAGTCTAACGATTTCTGTATCCAGATCCAGCTTGTCCCTCGCATCTA
CTATCACCCAGACGGTACCTTGCTACAGGCCTATGAGTCCCCCACCCTAGAAACAAGAGA
GAGCCTGTCTCACTCACCCTGGCTGTCCTTCTCGGATTAGGGGTCGCAGCAGGTATAGGTA
CCGGCTCGACCGCCCTAATAAAAGGGCCCATAGACCTCCAACAAGGTTTGACTAGCCTCC
AGATTGCCAGGTGGATACAGACCTTAGGGCCCTTCAAGACTCCATAAGTAAACTAGAGGATT
CCTTAACCTCCCTGTCTGAAGTAGTGCTCCAGAATAGGAGAGGCCTTGATCTGCTATTTTGAA
GGAAGGGGGCCTTTGTGCAGCCCCTAAAAAAAAAAGAGGAATGCTGTTTCTATGTTTGACCA
CTCAGGCGCGGTGCGGGACTCCATGAGGAGACTCAAGGAAAGGTTAGATAAGAGGCAGTT
AGAGCACCAAAAGAATTTAAGTTGAGTACGAGGGATGGTTCAACCGTTCCCCCTGGCTTACT
ACTTTACTGTCTGCCCTTGCTGGTCCCCTGCTACTCCTCCTTCTGTTACTCACCCTCGGGCC
TTGTGTCATCAATAAGTTAGTGCAATTCATCAATGATAGGGTTAGTGCAGTAAGGATTCTGGTT
CTCAGGCACAAGTACCAGACCCTAGACAACGAGGATAACCTTTAATTCTGCTCTATGATTAG
AGCTACCCTCAAAAGAAAATGGGGGAATGAAGGAGGCAGAAATCATGAGGCAGAAATCATT
CCGTGAAGTATGGAAACTACCCGGAGGGCCCAAGGTTTAGGGACAGGTGCAGCCAGGCA
CAGTAAAAGGTCAGAGCAAGAAAAACAAGGAAGATTTGGAGTGCCAAACAGAATATCTGTGG
TCATGCACCTGAGTCCCCACCCCGGACTTATGCAAACAATTCCCAGAAATAGCTGAGCTCA
TAACAGTTTCTAGGGTGCCCCTCAGCAGTTTCTAGAACCCTCTCGTGACCGGAGTTTTTATTC
AAACTAACCAATGATCTTGCTCCTCGCTTCTGTACCCGCGCTTTTTGCTATAAAATGAGACCA
AAGAATCCACCCGGCGCGCCAGTCCCTCTAGGTGACTGAGTCGCCCGAGTACTCGTAAGT
TCAATAAACCTCTTGCTATTTGCATCCGGAGTTGTGTTCGCGTTGATCCTGGGAGGGTTTCTC
AAGGTCGGTGGACTACCCGAACATCAGGGTCTTTCAG
